# Microstructural and chemical characterization of radiation-induced carious dentin of teeth submitted to ionizing radiation as a head and neck cancer therapy

**DOI:** 10.1371/journal.pone.0337062

**Published:** 2025-12-12

**Authors:** Bruna L. Nascimento, Paulo Soares, Rodrigo N. Rached, Paulo H. Couto Souza, Luciana R. A. Alanis, Evelise M. Souza

**Affiliations:** 1 Graduate Program in Dentistry, School of Medicine and Life Sciences, Pontifícia Universidade Católica do Paraná (PUCPR), Curitiba, Paraná, Brazil; 2 Graduate Program in Mechanical Engineering, Pontifícia Universidade Católica do Paraná (PUCPR), Curitiba, Paraná, Brazil; Indiana University School of Dentistry, UNITED STATES OF AMERICA

## Abstract

Radiation-induced caries is a common side effect of radiotherapy for head and neck cancer caused by pulpal hypovascularization and dentin collagen degradation by the direct action of ionizing radiation. A thorough understanding of radiation-induced changes in dental structure can lead to more effective and durable restorative treatments. The objective of this study was to analyze the microstructure and chemical composition of radiation-induced carious dentin (CD) compared with irradiated (ID) and sound dentin (SD) by using analytical techniques, including scanning electron microscopy (SEM), energy-dispersive spectroscopy (EDS), X-ray diffraction (XRD), and Fourier transform infrared spectroscopy (FTIR). Specimens of CD and ID were obtained from patients who had undergone radiotherapy. SD that had not been irradiated was used as a control group. The specimens were sectioned, fixated, and dehydrated according to each type of analysis and equipment. The SEM analysis revealed varying degrees of microstructural degradation in both CD and ID groups. The EDS analysis showed higher levels of carbon in both ID and CD (p < 0.05) and the levels of calcium and phosphorus were significantly higher in SD (p < 0.05). ID and CD demonstrated the absence of the 1-1-2 and 3-0-0 peaks in the XRD analysis and less pronounced carbonate peaks in FTIR. The results are correlated with changes in the composition of the crystalline and collagen structure found in CD and ID. These morphological and chemical changes suggest that radiation might cause a reduction in the quality of hydroxyapatite and collagen degradation, which could negatively affect the effectiveness and decrease the longevity of adhesive restorative treatments of irradiated teeth.

## Introduction

Head and neck cancer is the sixth most common malignancy worldwide, with approximately 650,000 new cases diagnosed annually [1,2]. For the treatment of head and neck cancer, surgical resection is often combined with radiotherapy and/or chemotherapy [3]. Radiotherapy is one of the most common treatment options for head and neck cancer usually resulting in good rates of tumor control [4]. Despite being a non-invasive treatment option due to the preservation of the tissue structure, some side effects in sites can develop up to 12 months after the completion of treatment [5,6]. These side effects may include mucositis, taste loss, hyposalivation, radiation-induced caries, periodontal disease, osteoradionecrosis, and trismus [6].

Radiation-induced caries has been considered one of the most common side effects of head and neck radiotherapy [7,8] and reported in approximately 29% to 37% of patients [9]. Clinically, these lesions begin in the labial surface at the cervical third of the teeth with superficial enamel changes and brownish pigmentation [8]. With the development of the lesion, areas of enamel demineralization and delamination occur, leading to a possible dental crown amputation [10,11].

Radiation-induced caries have a multifactorial etiology from a combination of direct damage and indirect effects. Hyposalivation impairs enamel remineralization and reduces saliva’s antimicrobial properties. Poor oral hygiene allows cariogenic bacteria to grow, while a carbohydrate-rich diet acts as a substrate for acid production, accelerating tooth demineralization. The direct ionizing radiation affects the hard tissues of the teeth [6,12].

The destruction of collagen found within the dental pulp of teeth located in the irradiation field appears to contribute to fibrosis and a reduction of vascularity within the dental pulp, especially by higher radiation doses (over 50Gy), impairing the metabolism of odontoblasts, which are responsible for dentin production and mineralization [12,13]. Several studies have reported changes in the mechanical and chemical properties of dental hard tissues caused by the direct effect of radiation, such as microhardness, elastic modulus, and chemical composition [14,15].

Collagen degradation in the organic matrix involves the chemical reaction known as decarboxylation, which is based on removing carboxyl groups (COOH). When collagen is exposed to ionizing radiation, decarboxylation of specific amino acid side chains takes place releasing carboxyl groups and simultaneously causing the loss of phosphate groups, directly associated with the collagen matrix. Additionally, during this process, new phosphate groups are formed, which can bridge with calcium ions, changing the structural integrity of collagen and contributing to its breakdown [16].

Studies investigating the properties of dental tissues using natural teeth with radiation-induced caries are very scarce. Most studies use sound-extracted teeth subjected to different radiotherapy protocols [17–21]. These simulation methods have limitations since they do not reproduce the oral environment, such as the presence of saliva, bone, and oral tissues, inherent patient factors, type of diet, and oral hygiene. Therefore, the outcomes of these types of research may not contribute to the complete understanding of the etiopathogenesis of radiation-induced caries and indicate more effective restorative treatments.

Understanding how dental tissue changes after radiation is important for establishing an effective treatment protocol that restores function, aesthetics, and phonetics and improves the quality of life of patients affected by radiation caries. Therefore, this exploratory study aimed to analyze the microstructure and changes in the chemical composition of radiation-induced carious dentin and irradiated dentin from patients who underwent head and neck radiotherapy compared to sound dentin. The null hypothesis posits that there would be no difference in the microstructural and chemical analyses of the substrates after scanning electron microscopy (SEM), energy-dispersive spectroscopy (EDS), X-ray diffraction (XRD), and Fourier transform infrared spectroscopy (FTIR) analysis.

## Materials and methods

### Ethical considerations and patients’ recruitment

Ethical approval for this study involving human teeth was granted by the research ethics committee of Pontifícia Universidade Católica do Paraná (No. 4.615.561). Patients who had undergone head and neck radiotherapy donated teeth affected by radiation-induced caries after reading and signing a written informed consent containing detailed information about the research. Sound teeth were obtained from an institutional tooth bank. All teeth were stored in 0.5% chloramine-T solution at 4°C for up to 3 months before the tests. This solution was used as a disinfectant and preservative media to help inhibit microbial growth and prevent tissue breakdown during storage [22].

The specimens were collected from March to June 2023 during the dental treatment of systemic compromised patients at PUCPR Dental Clinic. Patients who donated the specimens had a history of head and neck tumors and were previously treated with a radiotherapy regimen of 2 Gray per day, five times a week, for 6–7 weeks. The age range of the patients was 35–65 years old, both male and female.

### Specimens’ preparation

Six carious teeth were used to obtain six fragments of radiation-induced carious dentin (CD) and six fragments of adjacent irradiated dentin (ID). For the control group (SD), six sound teeth were also sectioned to produce six fragments of sound dentin, matching the number of specimens obtained from the irradiated teeth. The specimens were sectioned using a diamond disk to obtain 3-mm thick specimens under constant water cooling in a precision cutting machine (IsoMet 1000, Buehler Ltd., Lake Bluff, IL), and then embedded in self-curing acrylic resin (Jet, Classico, São Paulo, Brazil) inside a 3-mm high plastic tube. The surfaces of all specimens were ground flat and polished using a metallographic polisher (Ecomet 30, Buehler Ltd., Lake Bluff, IL) with #600 silicon carbide sandpaper for 30 s, #1200 for 5 min, and #2000 for 10 min. Subsequently, the specimens were polished with 3-, 1-, and 0.25-µm diamond pastes (Metadi II, Buehler Ltd., Lake Bluff, IL) for 15 s each. The specimens were ultrasonically cleaned for 10 min with distilled water between each sandpaper and paste, and for 20 min after the completion of polishing procedure.

**Fig 1:**
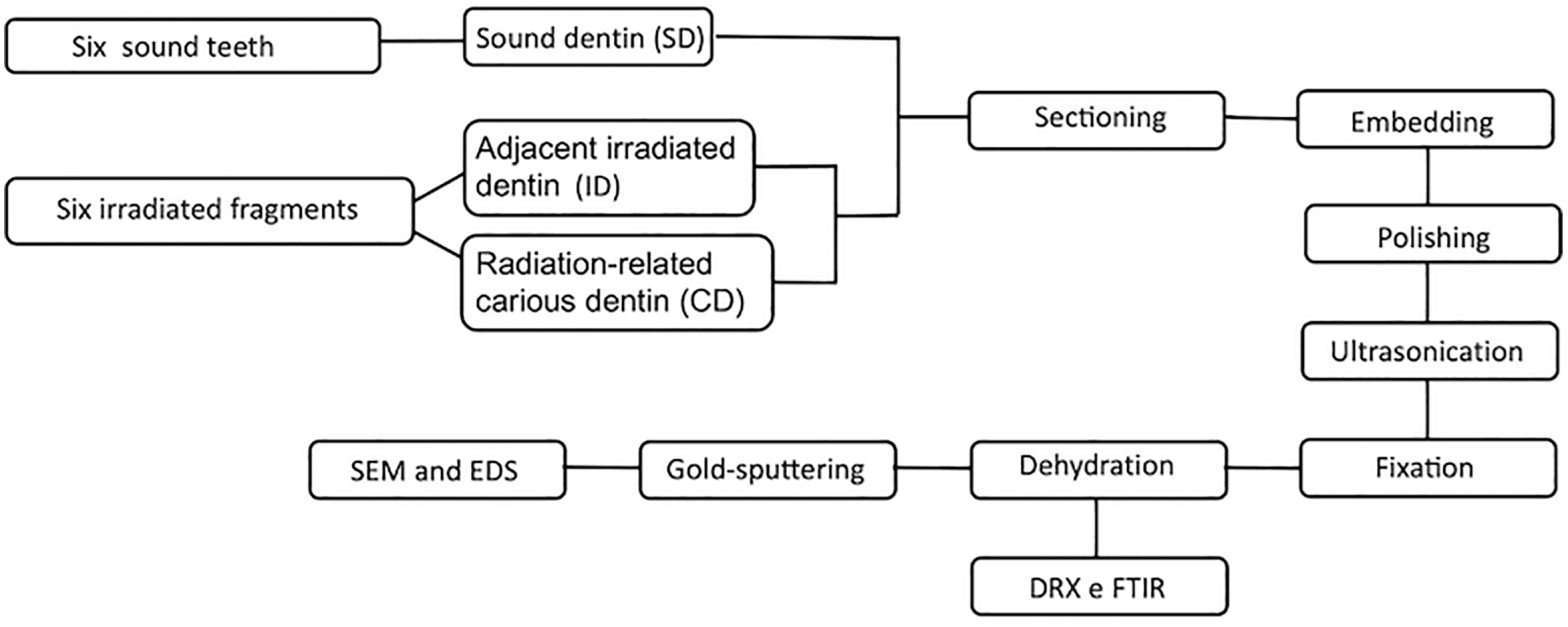
Study design and steps of specimens’ preparation and analyses.

The specimens were prepared for the tests according to a previous study [23]. Initially, the dentin surfaces were etched with 10% phosphoric acid for 5 s and washed for 15 s. This was followed by 10% sodium hypochlorite for 10 min and an ultrasonic bath for 20 min. Specimens were then fixed by immersion in 2.5% glutaraldehyde for 24 h to preserve structural integrity. Dehydration was conducted through sequential immersion in graded alcohol concentrations, beginning with 25%, 50%, and 75% for 20 min each, followed by 95% for 30 min, and concluding with 100% for 1 h. Finally, the specimens were immersed in hexamethyldisilazane (Merck KGaA, Darmstadt, Germany) for 10 min and kept in a desiccator with silica to avoid humidity until testing. Fig 1 illustrates the study design along with the detailed steps involved in the specimens’ preparation and analysis.

### Scanning electron microscopy (SEM) and energy dispersive X-ray spectroscopy (EDS) analysis

SEM was used to obtain high-resolution images of the microstructure characterization and surface morphology of carious, irradiated, and sound dentin, providing details of microcracks, demineralization zones, and other defects. Three specimens from each group were sputter-coated with a 3.3 nm thickness gold alloy using a sputtering machine (Quorum Q150V sputter coating, UK). Electron microscopy (Tescan, Vega3, Czech Republic) was performed using SE mode at 20 kV with 1000 × , 10000 × , and 25000 × magnifications.

EDS was used to analyze the chemical composition and to compare the differences in the dentin substrates, identifying key elements like calcium, phosphorus, and carbon, essential for understanding dentin’s mineral content. For this purpose, an X-ray detector system coupled to an electron microscope (X-act, Oxford Instruments, UK) operated at 20 kV using a 5-nm focal length with a magnification of 1000 × was used. This voltage ensured sufficient energy to detect key elements, providing optimal sample penetration, and minimizing potential damage or artifacts. A short focal length improved spatial resolution, allowing precise localization of elemental composition at a fine scale. Data on the absolute proportion of the elements calcium (Ca), phosphorus (P), carbon (C), and oxygen (O) were obtained, according to their weight content in percentages.

### X-ray diffraction analysis (XRD)

Specimens were prepared following the previously described methodology for X-ray diffraction (XRD7000 diffractometer, Shimadzu Corp., Kyoto, Japan). The analysis was conducted by CuKα radiation, with the X-ray source operating at 40 kV and 30 mA. Data collection was performed over a 2θ range of 10° to 70°, employing a continuous scanning speed of 2°/min to ensure high-resolution diffraction patterns.

### Fourier transform infrared spectroscopy analysis (FTIR)

Attenuated Total Reflectance (ATR) is an FTIR technique in which infrared light is reflected within a crystal, slightly penetrating the sample surface. This causes molecular vibrations that reveal the material’s chemical composition. ATR-FTIR is useful for analyzing dentin and other materials with minimal sample preparation and detecting functional groups and chemical changes. An Attenuated Total Reflectance-Fourier Transform Infrared spectroscopy (ATR-FTIR) (PerkinElmer Frontier, Waltham, MA, USA) with a 10-cm focal length lens, equipped with a GaSe Diamond/ZnSe crystal was used to analyze hydroxyapatite (Hap) vibration bands. The equipment was operated in a temperature- and humidity-controlled room. Each surface tested was carefully positioned against the ATR unit’s diamond crystal, with constant pressure applied to provide contact. For each sample, 32 scans were averaged to improve the signal-to-noise ratio. Spectra were acquired from the flat region of each sample.

### Statistical analysis

Statistical analysis was performed by the *Statistical Package for Social Sciences* software, version 26 (SPSS, IBM Corp., Armonk, NY). The EDS results were analyzed using the nonparametric test of Kruskal–Wallis, due to the lack of normal distribution of the data. The comparison between groups was performed using the Dunn pairwise test. All tests were performed at a 5% significance level, indicating that results were considered statistically significant if p < 0.05.

## Results

### SEM and EDS analyses

**Fig 2:**
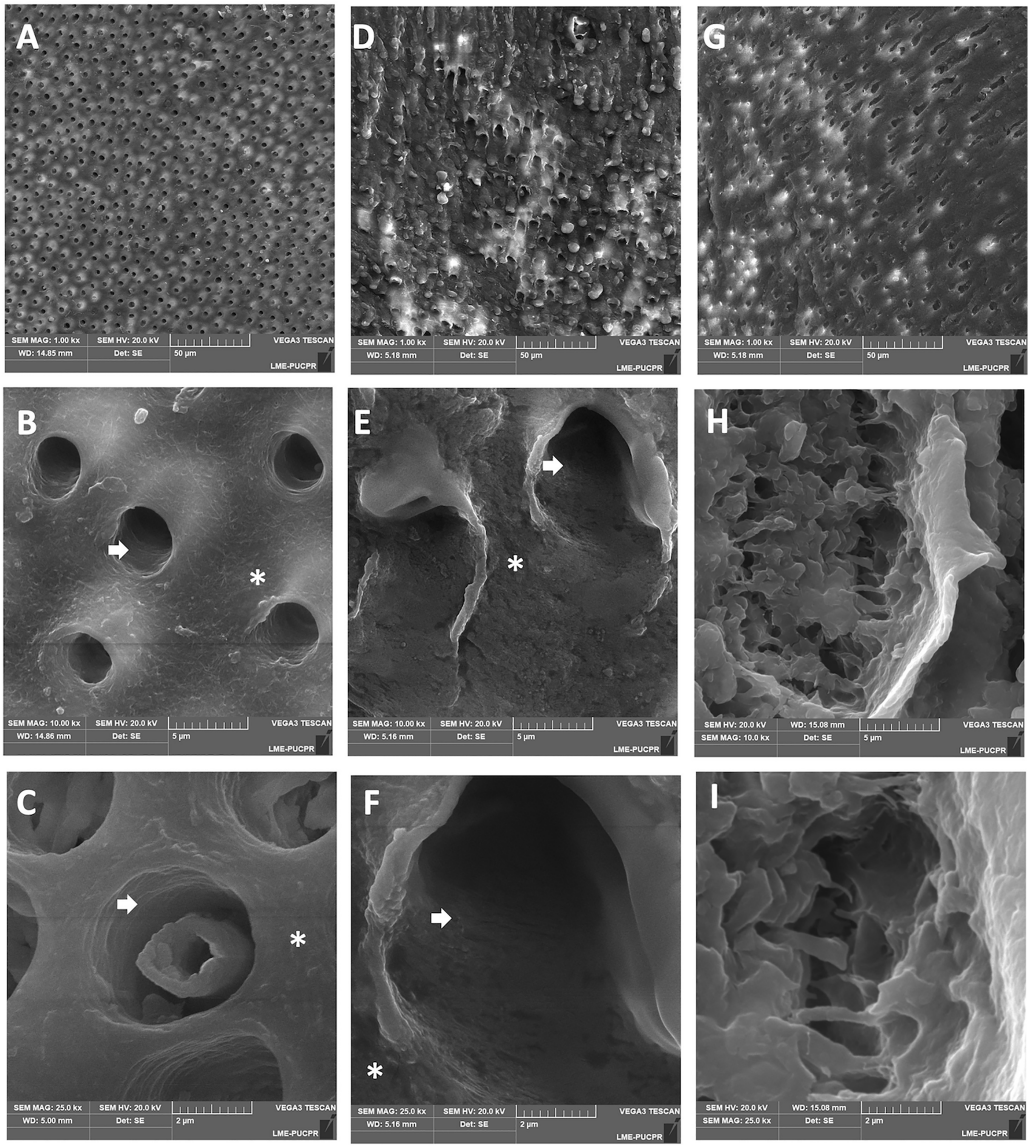
SEM representative images of specimens from the evaluated groups. **(A)** Sound dentin (SD) exhibiting normal organization of dentin with open tubules (1000×). **(B)** Intertubular dentin (asterisk) and peritubular dentin (arrows) (10000×). **(C)** Odontoblast process inside a tubule (25000×). **(D)** Adjacent irradiated dentin (ID) shows irregularities and partially obliterated dentin tubules. **(E)** and **(F)** Peritubular dentin at the tubule internal walls showing signs of degradation (arrows) (10000× and 25000×). **(G)** Radiation-related carious dentin (CD) showing partially obliterated tubules (1000×). **(H)** and **(I)** Radiation-related carious dentin (CD) with numerous craters in the peritubular dentin (10000× and 25000×).

The dentin morphology of specimens from the evaluated groups analyzed by SEM is presented in Fig 2. Sound dentin (SD) exhibiting surface integrity (Fig 2A and 2B) of the intertubular (asterisk) and peritubular dentin (arrows) with the view of the odontoblastic process (Fig 2C) and absence of visible craters and microfractures. Adjacent irradiated dentin (ID) presented craters with dentin tubules partially obliterated (Fig 2D–2F). Radiation-induced carious dentin (CD) with the presence of craters (Fig 2G). Fig 2H and 2I illustrate that CD exhibits numerous craters indicative of dental degradation. However, the distinction between intertubular and peritubular dentin remains unclear.

Table 1 presents the weight percentage (wt%) of calcium (Ca), phosphorus (P), carbon (C), and oxygen (O) as determined by Energy Dispersive Spectroscopy (EDS). The results indicate that the atomic percentages of Ca and P decreased significantly when comparing samples labeled as SD and CD, with p-values of 0.027 and 0.039 respectively. Conversely, the weight percentage of C increased significantly between these two groups (p = 0.027). However, no statistically significant difference was observed in the O percentages (p = 0.066).

**Table 1:** Weight percentage (wt%) ± standard deviation of calcium (Ca), phosphorus (P), carbon (C), and oxygen (O) of SD, ID and CD groups as determined by Energy Dispersive Spectroscopy (EDS).

Elements	SD	ID	CD
**Ca**	22.80 ± 2.45 a	11.80 ± 2.29 ab	5.43 ± 2.68 b
**P**	13.40 ± 1.41 a	4.83 ± 0.86 ab	2.93 ± 1.11 b
**C**	19.90 ± 1.92 a	46.17 ± 2.30 ab	59.23 ± 8.44 b
**O**	43.33 ± 1.40 a	30.30 ± 7.29 a	28.20 ± 8.93 a

Different letters indicate statistically significant differences between the dentin substrates for the same chemical element (p<0.05).

After the statistical analysis, the Ca/P/C ratio was calculated. The Ca/P/C ratio was 0.09 in SD, 0.06 in ID, and 0.03 in CD.

### XRD analysis

**Fig 3:**
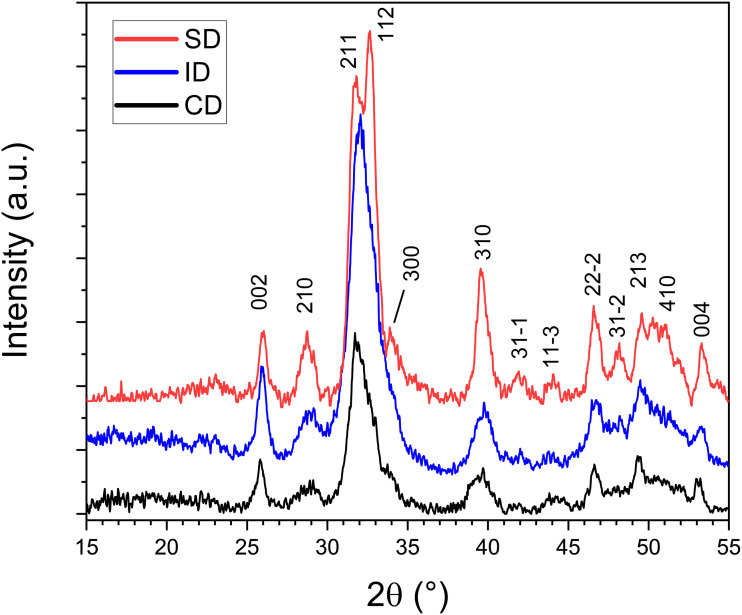
XRD patterns of the dentin specimens of the evaluated groups. HAP was identified using the ICDD [01-072-7552] file.

Fig 3 shows the XRD spectra of the dentin surface of specimens from the SD, ID, and CD groups. Miller’s (hkl) indices shown in the graph were indexed according to the ICDD file number 01-072-7552 for Hydroxyapatite. Most of the identified peaks resulted in a decrease in ID and CD specimens compared to SD. We observed an absence of the 1-1-2 and 3-0-0 peaks in ID and CD specimens. Most of the other denoted peaks presented a reduction in intensity for ID and predominantly for CD.

### FTIR analysis

**Fig 4:**
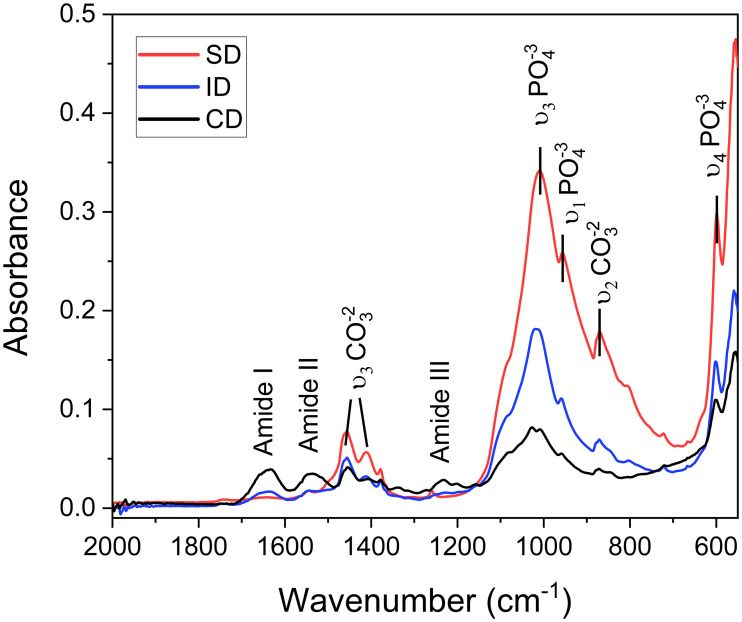
FTIR spectra of specimens from each group showing HAP vibration bands.

The representative spectra of hydroxyapatite in specimens from the evaluated groups are presented in Fig 4. The organic phase of dentin, mainly composed of type I collagen, is represented by specific spectral bands known as Amide I (1600–1700 cm ⁻ ¹), Amide II (1500–1600 cm ⁻ ¹), and Amide III (1200–1350 cm ⁻ ¹). The intensity of peaks was similar for SD and ID samples, while CD exhibited higher peak intensity, especially in the Amide I and II bands. The spectra of ID and CD showed peaks associated with phosphate vibrations (PO_4_^3^), ν₁ 940–980 cm ⁻ ¹, ν₃ 1000–1100 cm ⁻ ¹, ν₄ 500–600 cm ⁻ ¹ bands. While the distinct bands attributed to carbonate vibrations (CO_3_^2-^) ν₂ 850–890 cm ⁻ ¹, ν₃ 1400–1500 cm ⁻ ¹ bands are less pronounced in these groups compared to SD.

## Discussion

This study focused on the chemical characterization and structure analysis of sound, adjacent irradiated dentin, and radiation-induced carious dentin. According to the results, there were changes in the morphological structure and chemical composition of the adjacent irradiated dentin and radiation-induced carious dentin. Therefore, the null hypothesis that there would be no difference in the microstructural and chemical analyses between the substrates was rejected.

The demineralization patterns of radiation-induced caries appear like those of conventional caries, characterized by demineralized dentin, translucent zones, dentin dead tracts, and the deposition of reactionary and intratubular dentin [24]. The presence of the odontoblastic process in sound dentin images and its absence in adjacent irradiated dentin and radiation-induced carious dentin likely reflect the disruption of metabolic activity in odontoblasts, as these processes are essential for maintaining dentin-pulp homeostasis. This absence may indicate impaired nutrient transport and reduced cellular activity, directly correlating with altered pulp vitality and decreased odontoblastic metabolism due to radiation exposure [12,13]. Although some laboratory simulations of radiotherapy studies have observed similarities between sound dentin and radiation-induced carious dentin [25,26], our SEM images showed obliteration, and craters in the adjacent irradiated dentin and more numerous and deeper craters in radiation-induced carious dentin. Our findings suggest that ionized radiation therapy leads to demineralization and degradation of the dentin, compromising its structure mechanically and increasing susceptibility to further damage or decay. A previous study analyzing the effect of radiation therapy on the morphology of enamel and dentin of deciduous teeth in SEM also reported dentin with fissures, tubule obliteration, and fragmentation of collagen fibers observed especially after higher cumulative doses [27].

In laboratory studies on the effects of radiotherapy [18,21,28], extracted sound human teeth are used, and the oral conditions are simulated either with a physiological sodium chloride solution immersion [21], relative humidity [18], or even under dry conditions [28], and the interference of natural tissues is not reproduced at all. Therefore, in the present study teeth from patients who received radiotherapy and developed radiation-induced caries were used. One of the radiotherapy’s most important local effects is demineralization of dental tissues by changing both the organic and inorganic components [18,26]. This effect can be exacerbated by hyposalivation and xerostomia, which has been reported in almost 100% of the patients undergoing head and neck radiotherapy and interferes directly with the mineral balance and pH of the oral environment [29].

The significant decrease in Ca and P content in radiation-induced carious dentin has been associated with the effects of ionizing radiation on hydroxyapatite [28]. Besides that, our results also showed a decrease in Ca and P content in the adjacent irradiated dentin. The Ca/P/C ratio is associated with the mineral composition of the crystalline structure of the teeth, which directly affects the mechanical properties of dental hard tissues [26,30]. The increased carbon (C) content reflects changes in organic components, which may contribute to tissue breakdown**.** A recent study reported a non-significant difference in the Ca/P ratio between irradiated and non-irradiated root dentin. However, the samples were obtained by sectioning impacted third molars and the radiation was performed under dry conditions [31]. Indeed, interactions between water and radiation exposure have been reported, in which ions are released by water, inducing the formation of a distinct nanostructured phase, known as the secondary nanoapatitic calcium phosphate phase, which is primarily composed of nano-sized apatite crystals, leading to an increase in the degradation susceptibility of Hap [32,33].

Magnesium is important in Hap crystal growth, replacing calcium ions in the hydroxyapatite structure. This substitution helps stabilize the crystal lattice and influences Hap crystals’ size, shape, and overall development [34]. However, its level was below the detectable threshold in our study, so we did not include it in the results.

X-ray diffraction (XRD) was used to analyze dentin because it provides detailed information on its mineral composition, crystal structure, and crystallinity, assessing changes in dentin due to demineralization, aging, or treatments [35]. The interaction between the X-rays and the specimen resulted in a diffraction pattern of the inorganic composition of Hap. The most visible differences among the spectra of Fig 3 are the disappearance of the (1 1 2) and (3 0 0) peaks. These changes in the adjacent irradiated dentin indicate preferential damage or dissolution of crystal peaks, likely influenced by their stability or orientation within the dentin structure. Additionally, there was a clear difference in peak intensity among the three dentin substrates, particularly at (0 0 2), (2 1 0), (3 1 0), and (2 2 2) peaks, suggesting damage to Hap crystals and degradation in the adjacent irradiated dentin, and even more in the radiated-induced carious dentin. This reduction may result from a decrease in crystallite size, an increase in lattice strain, or a combination of both, potentially caused by direct radiation-induced damage or demineralization processes [27].

FTIR analysis explored the inorganic and organic parts of the dentin, including collagen. The crystallinity of dentin and the presence of collagen crosslinks are identifiable in FTIR spectra, where the degradation of dentin is characterized by lower crystallinity and higher collagen crosslinks [35]. Collagen cross-linking involves forming covalent or non-covalent bonds between collagen molecules, enhancing tissue strength and stability, which is important for resistance to degradation and dentin bonding durability [36]**,** as well as hardness and resistance to mechanical stress [37]**.** The amide bands I, II, and III, characteristic of collagen’s triple helix structure, are primarily associated with the protein components of materials like collagen, a major organic component in dentin [38]**.** Changes in the amide bands, particularly Amide I and Amide II, can reflect modifications in the collagen structure [39]**.** Qualitative differences can be observed in the amide band regions of the spectra between the sound, irradiated, and carious dentin groups, with higher absorbance in amide for carious dentin.

The combined analysis of phosphate and carbonate vibrations provides a comprehensive understanding of how caries-induced degradation impacts dentin mineral content and structural integrity [33]. Changes in FTIR spectra intensity suggest a potential decrease in the relative quantity of carbonate incorporated within the dentin structure following irradiation and caries development. Carbonate groups are often substituted into hydroxyapatite, affecting its biological and mechanical properties and indicating changes in the mineral composition [38]**.** The apparent reduction in the intensity of carbonate bands aligns with the concept of radiation and caries-induced mineral loss and alteration in dentin composition. Phosphate groups are hydroxyapatite’s primary mineral component, forming dentin’s structural backbone. The presence and intensity of phosphate-specific vibrations are key indicators of the degree of mineralization [40]**.** In our results, the decrease in FTIR spectra intensity associated with phosphate groups (e.g., the region around ~1000−1100 cm-1) suggests alterations in the mineral content of the dentin. This outcome could be related to a demineralization process, where the mineral component of dentin is reduced.

The reduction in the intensity of peaks indicates a decrease in hydroxyapatite content, particularly in carious dentin specimens, which can suggest a weakened dentin structure caused by degradation. These findings are consistent with previous studies showing that radiation therapy causes significant alterations in tooth mineralization, compromising the dentin’s resistance to caries and fractures [18–20]. Due to the nature of our experimental design and the limited sample size, it was not possible to perform statistical analysis on the data. Spectral normalization would be a valuable step in allowing quantitative or comparative analysis of collagen degradation. Since it was not performed in the present study, making these statements could be inappropriate.

A previous study relates the significance of cross-linking in collagen fibers in dentin hardness and elastic modulus [41]. It explains that cross-linking involves the formation of covalent bonds between collagen molecules, with amides playing an important role. Enzymatically modified lysine and hydroxylysine residues form aldehydes, which react with neighboring residues to create stable cross-links, essential for a stable collagen network in dentin [42]. Additionally, hydrogen bonding within the protein backbone stabilizes collagen’s triple helix structure, indirectly supporting dentin resilience [42]. Changes in FTIR spectra intensity associated with phosphate groups indicate variations in hydroxyapatite concentration, reflecting demineralization, while alterations in amide bands signal collagen structure modifications, such as denaturation or degradation, due to factors like radiation or caries. Similarities in the spectral curves of irradiated dentin and non-irradiated dentin were reported previously, with an increase in the irradiated dentin curve primarily in the peaks of the bands, including phosphate [43]. The opposite was found in the results of the present study, with clear differences in the spectral bands for all tested substrates.

Radiation-induced caries is known for its destructive and quick progress when untreated [43]. Therefore, one of the limitations of this study was the difficulty in obtaining numerous and large fragments due to the fragility of the irradiated and carious dentin structure. Therefore, the results should be interpreted as exploratory, warranting further studies with larger samples. The reduction of sample size impaired the inclusion of spectral normalization which would enable a more robust and quantitative assessment of collagen and Hap degradation following irradiation and radiation-induced caries development. Future studies including spectral deconvolution, peak fitting, and specific collagen crosslink ratio calculations might provide more detailed information about the changes in mineral and organic dentin components submitted to irradiation and caries development.

Based on our results, it can be assumed that the adhesion of restorative materials to radiation-affected dentin surfaces is deeply compromised. Collagen degradation impairs the chemical and mechanical interaction of the resin monomers of adhesive materials with the dental structure, which is essential to the retention and integrity of dental restorations [44]. The management and restorative treatment of radiation-induced caries is still challenging for dental professionals [45]. Clinical trials investigating the longevity of restorative treatments in patients with radiation-induced caries reported failures such as marginal integrity, secondary caries, and retention, with different adhesive restorative materials [45–48]. It could be inferred that the restorative treatment is significantly influenced by the complex and still poorly understood nature of the substrate available for adhesion, which directly impacts the longevity of restorations [49]. Therefore, the current study was designed to contribute to a better understanding of the impact of head and neck radiation on dentin tooth structure in both radiation-induced carious dentin and adjacent areas. It is important to comprehend the characteristics of dental substrates affected by radiation to recommend the proper treatment aiming to increase the longevity of restorations, decrease repetitive restorative cycles, and improve the quality of life of patients after oncologic treatment.

## Conclusion

The effect of radiation on the dentin was expressed chemically as a reduction in calcium (Ca) and phosphorus (P) levels in hydroxyapatite and microstructurally by the presence of tubule obliteration, and craters.

According to the XRD results the intensity of most peaks was decreased in irradiated dentin, and predominantly carious dentin, suggesting degradation of hydroxyapatite in these specimens.

FTIR findings showed a reduction of the intensity of carbonate bands leading to caries-induced mineral loss and alteration in dentin composition of both irradiated and carious dentin. The XRD and FTIR results suggest the degradation of collagen and Hap of the irradiated and carious dentin.

## Supporting information

S1 Data Raw data EDS. (ZIP)

S2 Data EDS statistics. (XLSX)

S3 Data Raw data XRD. (ZIP)

S4 Data Raw data FTIR. (ZIP)

S5 Data SEM part 1. (ZIP)

S6 Data SEM part 2. (ZIP)

## References

[1] Fregnani ER, Parahyba CJ, Morais-Faria K, Fonseca FP, Ramos PAM, de Moraes FY, et al. IMRT delivers lower radiation doses to dental structures than 3DRT in head and neck cancer patients. Radiat Oncol. 2016;11(1):116. doi: 10.1186/s13014-016-0694-7 27604995 PMC5015339

[2] Fonsêca J-M, Palmier N-R, Silva W-G, Faria K-M, Vargas P-A, Lopes M-A, et al. Dentin-pulp complex reactions in conventional and radiation-related caries: a comparative study. J Clin Exp Dent. 2019;11(3):e236–43. doi: 10.4317/jced.55370 31001393 PMC6461726

[3] Anderson G, Ebadi M, Vo K, Novak J, Govindarajan A, Amini A. An updated review on head and neck cancer treatment with radiation therapy. Cancers (Basel). 2021;13(19):4912. doi: 10.3390/cancers13194912 34638398 PMC8508236

[4] Huang S-H, O’Sullivan B. Oral cancer: current role of radiotherapy and chemotherapy. Med Oral Patol Oral Cir Bucal. 2013;18(2):e233-40. doi: 10.4317/medoral.18772 23385513 PMC3613874

[5] Specht L. Oral complications in the head and neck radiation patient. Introduction and scope of the problem. Support Care Cancer. 2002;10(1):36–9. doi: 10.1007/s005200100283 11777186

[6] Vissink A, Burlage FR, Spijkervet FKL, Jansma J, Coppes RP. Prevention and treatment of the consequences of head and neck radiotherapy. Crit Rev Oral Biol Med. 2003;14(3):213–25. doi: 10.1177/154411130301400306 12799324

[7] Eliasson L, Carlén A, Almståhl A, Wikström M, Lingström P. Dental plaque pH and micro-organisms during hyposalivation. J Dent Res. 2006;85(4):334–8. doi: 10.1177/154405910608500410 16567554

[8] Kielbassa AM, Hinkelbein W, Hellwig E, Meyer-Lückel H. Radiation-related damage to dentition. Lancet Oncol. 2006;7(4):326–35. doi: 10.1016/S1470-2045(06)70658-1 16574548

[9] Moore C, McLister C, Cardwell C, O’Neill C, Donnelly M, McKenna G. Dental caries following radiotherapy for head and neck cancer: a systematic review. Oral Oncol. 2020;100:104484. doi: 10.1016/j.oraloncology.2019.104484 31786391

[10] Palmier NR, Ribeiro ACP, Fonsêca JM, Salvajoli JV, Vargas PA, Lopes MA, et al. Radiation-related caries assessment through the International Caries Detection and Assessment System and the Post-Radiation Dental Index. Oral Surg Oral Med Oral Pathol Oral Radiol. 2017;124(6):542–7. doi: 10.1016/j.oooo.2017.08.019 29169512

[11] Fonseca J-M, Troconis C-C, Palmier N-R, Gomes-Silva W, Paglioni M-D, Araújo A-L, et al. The impact of head and neck radiotherapy on the dentine-enamel junction: a systematic review. Med Oral Patol Oral Cir Bucal. 2020;25(1):e96–105. doi: 10.4317/medoral.23212 31880287 PMC6982993

[12] Springer IN, Niehoff P, Warnke PH, Böcek G, Kovács G, Suhr M, et al. Radiation caries--radiogenic destruction of dental collagen. Oral Oncol. 2005;41(7):723–8. doi: 10.1016/j.oraloncology.2005.03.011 15979926

[13] Antic S, Markovic-Vasiljkovic B, Dzeletovic B, Jelovac DB, Kuzmanovic-Pficer J. Assesment of radiotherapy effects on the blood flow in gingiva and dental pulp - a laser Doppler flowmetry study. J Appl Oral Sci. 2022;30:e20220329. doi: 10.1590/1678-7757-2022-0329 36477557 PMC9724493

[14] Tu Y, Hao L, Ding Y, Zhong Y, Hua C, Jiang L. The influence of different radiotherapy doses on the mechanical properties and microstructure of the enamel and dentin of human premolar teeth. Strahlenther Onkol. 2024;200(12):1047–56. doi: 10.1007/s00066-024-02296-6 39283341

[15] Reed R, Xu C, Liu Y, Gorski JP, Wang Y, Walker MP. Radiotherapy effect on nano-mechanical properties and chemical composition of enamel and dentine. Arch Oral Biol. 2015;60(5):690–7. doi: 10.1016/j.archoralbio.2015.02.020 25766468 PMC4369427

[16] Hübner W, Blume A, Pushnjakova R, Dekhtyar Y, Hein H-J. The influence of X-ray radiation on the mineral/organic matrix interaction of bone tissue: an FT-IR microscopic investigation. Int J Artif Organs. 2005;28(1):66–73. doi: 10.1177/039139880502800111 15742312

[17] Lopes C de CA, Soares CJ, Lara VC, Arana-Chavez VE, Soares PB, Novais VR. Effect of fluoride application during radiotherapy on enamel demineralization. J Appl Oral Sci. 2018;27:e20180044. doi: 10.1590/1678-7757-2018-0044 30540070 PMC6296282

[18] Lu H, Zhao Q, Guo J, Zeng B, Yu X, Yu D, et al. Direct radiation-induced effects on dental hard tissue. Radiat Oncol. 2019;14(1):5. doi: 10.1186/s13014-019-1208-1 30635005 PMC6329176

[19] Duruk G, Acar B, Temelli Ö. Effect of different doses of radiation on morphogical, mechanical and chemical properties of primary and permanent teeth-an in vitro study. BMC Oral Health. 2020;20(1):242. doi: 10.1186/s12903-020-01222-3 32873280 PMC7465328

[20] Lieshout HFJ, Bots CP. The effect of radiotherapy on dental hard tissue--a systematic review. Clin Oral Investig. 2014;18(1):17–24. doi: 10.1007/s00784-013-1034-z 23873320

[21] Liang X, Zhang JY, Cheng IK, Li JY. Effect of high energy X-ray irradiation on the nano-mechanical properties of human enamel and dentine. Braz Oral Res. 2016;30:S1806-83242016000100209. doi: 10.1590/1807-3107BOR-2016.vol30.0009 26676192

[22] Mobarak EH, El-Badrawy W, Pashley DH, Jamjoom H. Effect of pretest storage conditions of extracted teeth on their dentin bond strengths. J Prosthet Dent. 2010;104(2):92–7. doi: 10.1016/S0022-3913(10)60098-4 20654765

[23] Thanatvarakorn O, Nakashima S, Sadr A, Prasansuttiporn T, Thitthaweerat S, Tagami J. Effect of a calcium-phosphate based desensitizer on dentin surface characteristics. Dent Mater J. 2013;32(4):615–21. doi: 10.4012/dmj.2013-073 23903644

[24] Kielbassa AM, Schendera A, Schulte-Mönting J. Microradiographic and microscopic studies on in situ induced initial caries in irradiated and nonirradiated dental enamel. Caries Res. 2000;34(1):41–7. doi: 10.1159/000016568 10601783

[25] Bohn JC, Chaiben CL, de Souza SS, Rumbelsperger AMB, Fernandes Â, Machado MÂN, et al. Conformational and constitutional analysis of dental caries following radiotherapy for head and neck cancer. Rep Pract Oncol Radiother. 2021;26(3):389–99. doi: 10.5603/RPOR.a2021.0046 34277092 PMC8281910

[26] de Siqueira Mellara T, Palma-Dibb RG, de Oliveira HF, Garcia Paula-Silva FW, Nelson-Filho P, da Silva RAB, et al. The effect of radiation therapy on the mechanical and morphological properties of the enamel and dentin of deciduous teeth--an in vitro study. Radiat Oncol. 2014;9:30. doi: 10.1186/1748-717X-9-30 24450404 PMC3905913

[27] Velo MM de AC, Farha ALH, da Silva Santos PS, Shiota A, Sansavino SZ, Souza AT, et al. Radiotherapy alters the composition, structural and mechanical properties of root dentin in vitro. Clin Oral Investig. 2018;22(8):2871–8. doi: 10.1007/s00784-018-2373-6 29430611

[28] Demirkan I, Yaprak G, Ceylan C, Algul E, Tomruk CO, Bilen B, et al. Acoustic diagnosis of elastic properties of human tooth by 320 MHz scanning acoustic microscopy after radiotherapy treatment for head and neck cancer. Radiat Oncol. 2020;15(1):38. doi: 10.1186/s13014-020-01486-7 32066465 PMC7027275

[29] Klimuszko E, Orywal K, Sierpinska T, Sidun J, Golebiewska M. Evaluation of calcium and magnesium contents in tooth enamel without any pathological changes: in vitro preliminary study. Odontology. 2018;106(4):369–76. doi: 10.1007/s10266-018-0353-6 29556861 PMC6153988

[30] Faustino I-S-P, Palmier N-R, Fernandes P-M, Ribeiro A-C-P, Brandão T-B, Santos-Silva A-R, et al. Morphological patterns of circumpulpal dentin affected by radiation-related caries. J Clin Exp Dent. 2020;12(5):e501–8. doi: 10.4317/jced.56584 32509234 PMC7263775

[31] Deniz Y, Aktaş Ç, Misilli T, Çarıkçıoğlu B. Effects of radiotherapeutic X-ray irradiation on cervical enamel. Int J Radiat Biol. 2021;97(12):1667–74. doi: 10.1080/09553002.2021.1987560 34586954

[32] Celik EU, Ergücü Z, Türkün LS, Türkün M. Effect of different laser devices on the composition and microhardness of dentin. Oper Dent. 2008;33(5):496–501. doi: 10.2341/07-127 18833855

[33] Liu Y, Yao X, Liu YW, Wang Y. A Fourier transform infrared spectroscopy analysis of carious dentin from transparent zone to normal zone. Caries Res. 2014;48(4):320–9. doi: 10.1159/000356868 24556607 PMC4422165

[34] Bystrov VS, Paramonova EV, Avakyan LA, Eremina NV, Makarova SV, Bulina NV. Effect of magnesium substitution on structural features and properties of hydroxyapatite. Materials (Basel). 2023;16(17):5945. doi: 10.3390/ma16175945 37687640 PMC10488744

[35] Toledano M, Aguilera FS, Osorio E, López-López MT, Cabello I, Toledano-Osorio M, et al. Submicron-to-nanoscale structure characterization and organization of crystals in dentin bioapatites. RSC Adv. 2016;6(51):45265–78. doi: 10.1039/c6ra02373h

[36] Hardan L, Daood U, Bourgi R, Cuevas-Suárez CE, Devoto W, Zarow M, et al. Effect of collagen crosslinkers on dentin bond strength of adhesive systems: a systematic review and meta-analysis. Cells. 2022;11(15):2417. doi: 10.3390/cells11152417 35954261 PMC9368291

[37] Gonçalves LMN, Palma-Dibb RG, Paula-Silva FWG, Oliveira HF de, Nelson-Filho P, Silva LAB da, et al. Radiation therapy alters microhardness and microstructure of enamel and dentin of permanent human teeth. J Dent. 2014;42(8):986–92. doi: 10.1016/j.jdent.2014.05.011 24887361

[38] Kolmas J, Piotrowska U, Kuras M, Kurek E. Effect of carbonate substitution on physicochemical and biological properties of silver containing hydroxyapatites. Mater Sci Eng C Mater Biol Appl. 2017;74:124–30. doi: 10.1016/j.msec.2017.01.003 28254276

[39] Rýglová Š, Braun M, Suchý T. Collagen and its modifications-crucial aspects with concern to its processing and analysis. Macromol Mater Eng. 2017;302(6):1600460. doi: 10.1002/mame.201600460

[40] Lopes C de CA, Limirio PHJO, Novais VR, Dechichi P. Fourier transform infrared spectroscopy (FTIR) application chemical characterization of enamel, dentin and bone. Appl Spectrosc Rev. 2018;53(9):747–69. doi: 10.1080/05704928.2018.1431923

[41] Rodrigues RB, Soares CJ, Junior PCS, Lara VC, Arana-Chavez VE, Novais VR. Influence of radiotherapy on the dentin properties and bond strength. Clin Oral Investig. 2018;22(2):875–83. doi: 10.1007/s00784-017-2165-4 28776096

[42] Yamauchi M, Sricholpech M. Lysine post-translational modifications of collagen. Essays Biochem. 2012;52:113–33. doi: 10.1042/bse0520113 22708567 PMC3499978

[43] Palmier NR, Migliorati CA, Prado-Ribeiro AC, de Oliveira MCQ, Vechiato Filho AJ, de Goes MF, et al. Radiation-related caries: current diagnostic, prognostic, and management paradigms. Oral Surg Oral Med Oral Pathol Oral Radiol. 2020;130(1):52–62. doi: 10.1016/j.oooo.2020.04.003 32444333

[44] Breschi L, Maravic T, Cunha SR, Comba A, Cadenaro M, Tjäderhane L, et al. Dentin bonding systems: from dentin collagen structure to bond preservation and clinical applications. Dent Mater. 2018;34(1):78–96. doi: 10.1016/j.dental.2017.11.005 29179971

[45] Wood RE, Maxymiw WG, McComb D. A clinical comparison of glass ionomer (polyalkenoate) and silver amalgam restorations in the treatment of Class 5 caries in xerostomic head and neck cancer patients. Oper Dent. 1993;18(3):94–102. 8415169

[46] Hu JY, Li YQ, Smales RJ, Yip KHK. Restoration of teeth with more-viscous glass ionomer cements following radiation-induced caries. Int Dent J. 2002;52(6):445–8. doi: 10.1111/j.1875-595x.2002.tb00640.x 12553399

[47] McComb D, Erickson RL, Maxymiw WG, Wood RE. A clinical comparison of glass ionomer, resin-modified glass ionomer and resin composite restorations in the treatment of cervical caries in xerostomic head and neck radiation patients. Oper Dent. 2002;27(5):430–7. 12216559

[48] De Moor RJG, Stassen IG, van ’t Veldt Y, Torbeyns D, Hommez GMG. Two-year clinical performance of glass ionomer and resin composite restorations in xerostomic head- and neck-irradiated cancer patients. Clin Oral Investig. 2011;15(1):31–8. doi: 10.1007/s00784-009-0355-4 19997859

[49] Dezanetti JMP, Nascimento BL, Orsi JSR, Souza EM. Effectiveness of glass ionomer cements in the restorative treatment of radiation-related caries - a systematic review. Support Care Cancer. 2022;30(11):8667–78. doi: 10.1007/s00520-022-07168-2 35657403

