# Supplementary figures and images for: Microstructural and chemical characterization of radiation-induced carious dentin of teeth submitted to ionizing radiation as a head and neck cancer therapy (part 1 of 2)
Source: PLoS One. 2025 Dec 12;20(12):e0337062. doi: 10.1371/journal.pone.0337062 (PMC12700452; doi:10.1371/journal.pone.0337062)

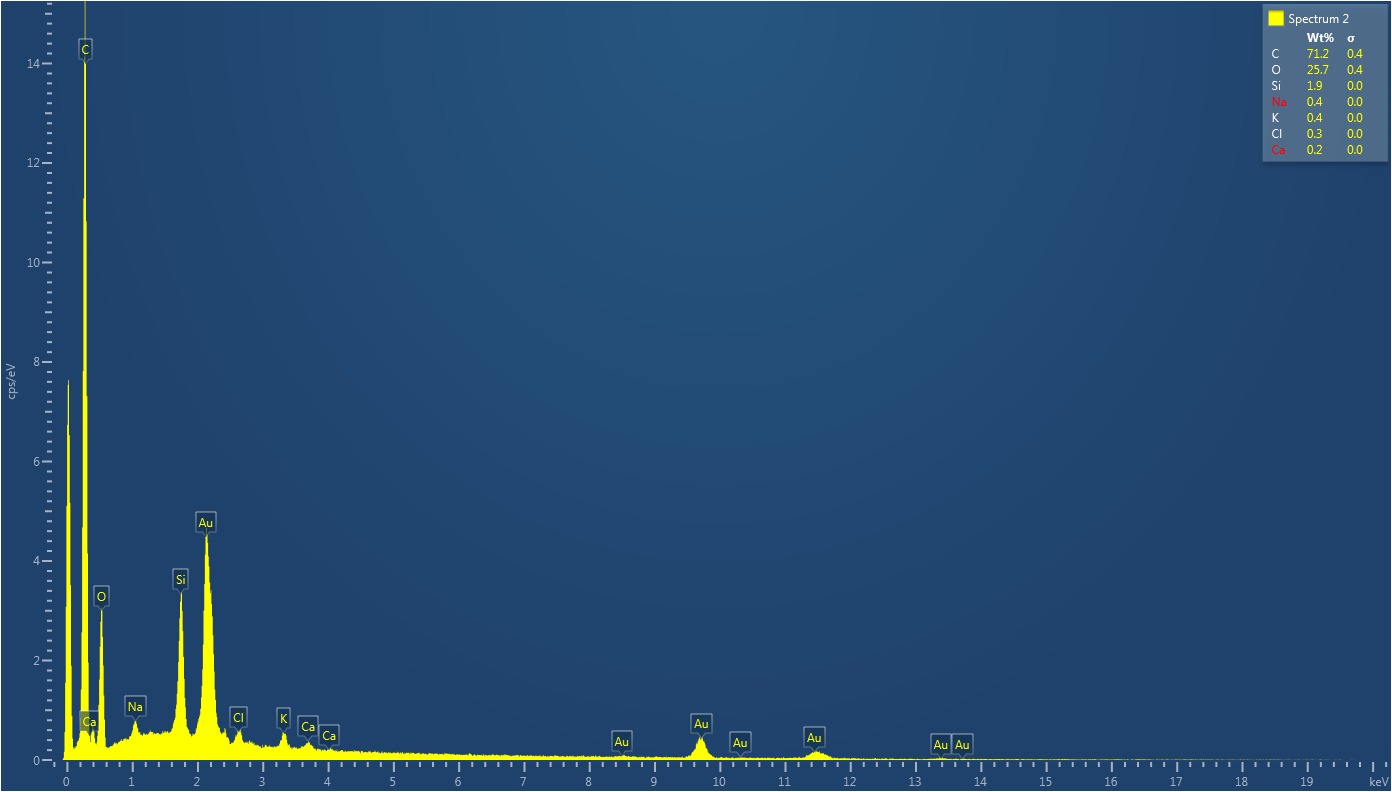

Supplement: S1 Data — (ZIP) [file pone.0337062.s001.zip › EDS/21Jan21/am1_crr_1kx_eds.tiff]

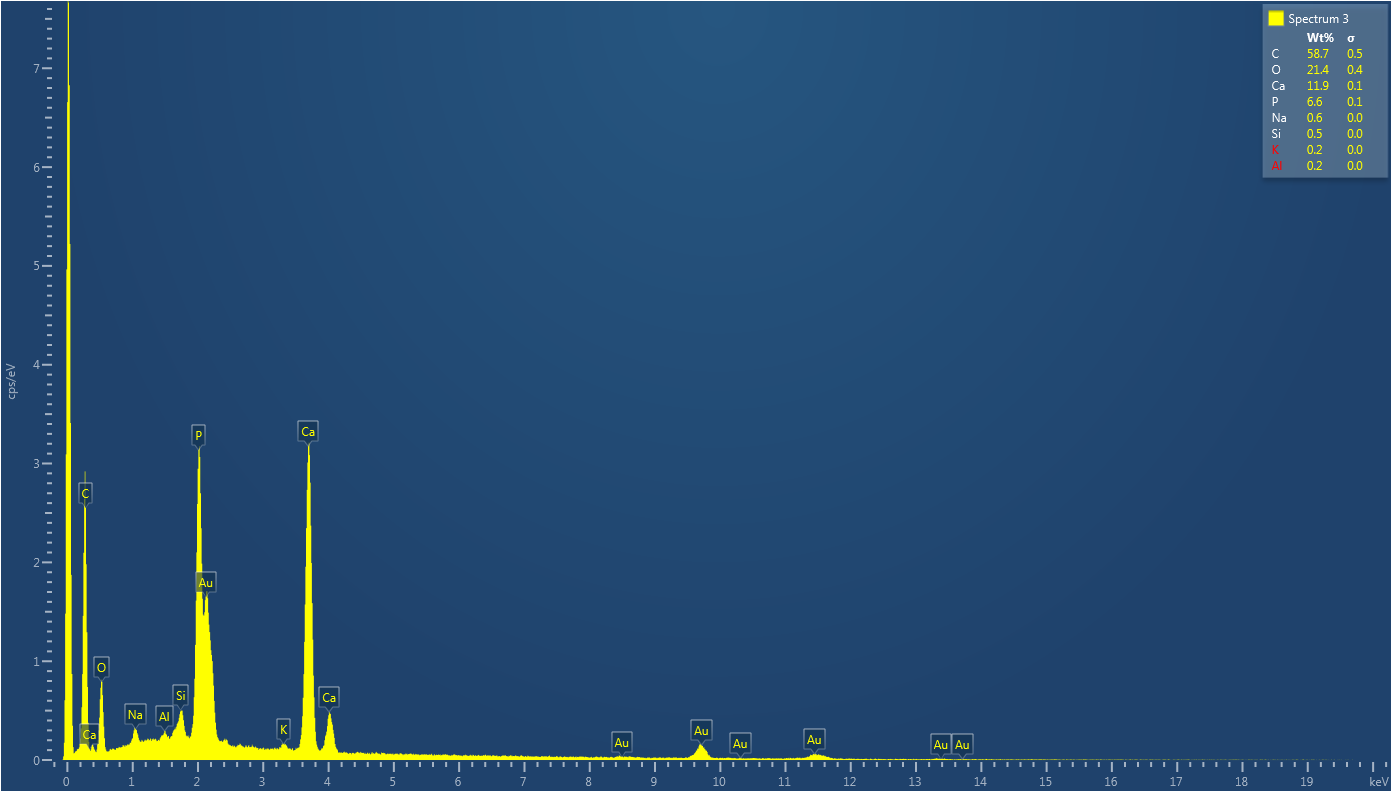

Supplement: S1 Data — (ZIP) [file pone.0337062.s001.zip › EDS/21Jan21/am1_higido_1kx_eds.tiff]

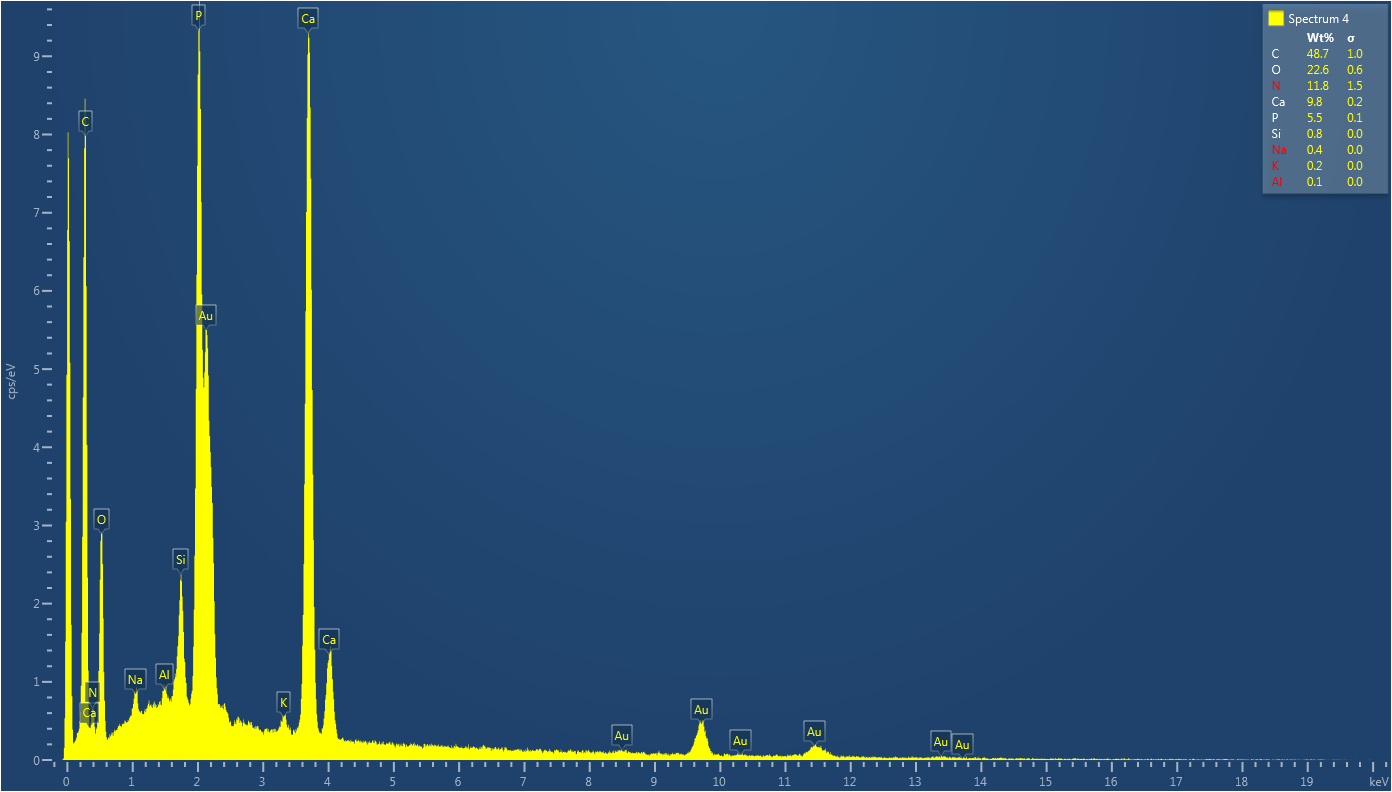

Supplement: S1 Data — (ZIP) [file pone.0337062.s001.zip › EDS/21Jan21/am2_higido_1kx_eds.tiff]

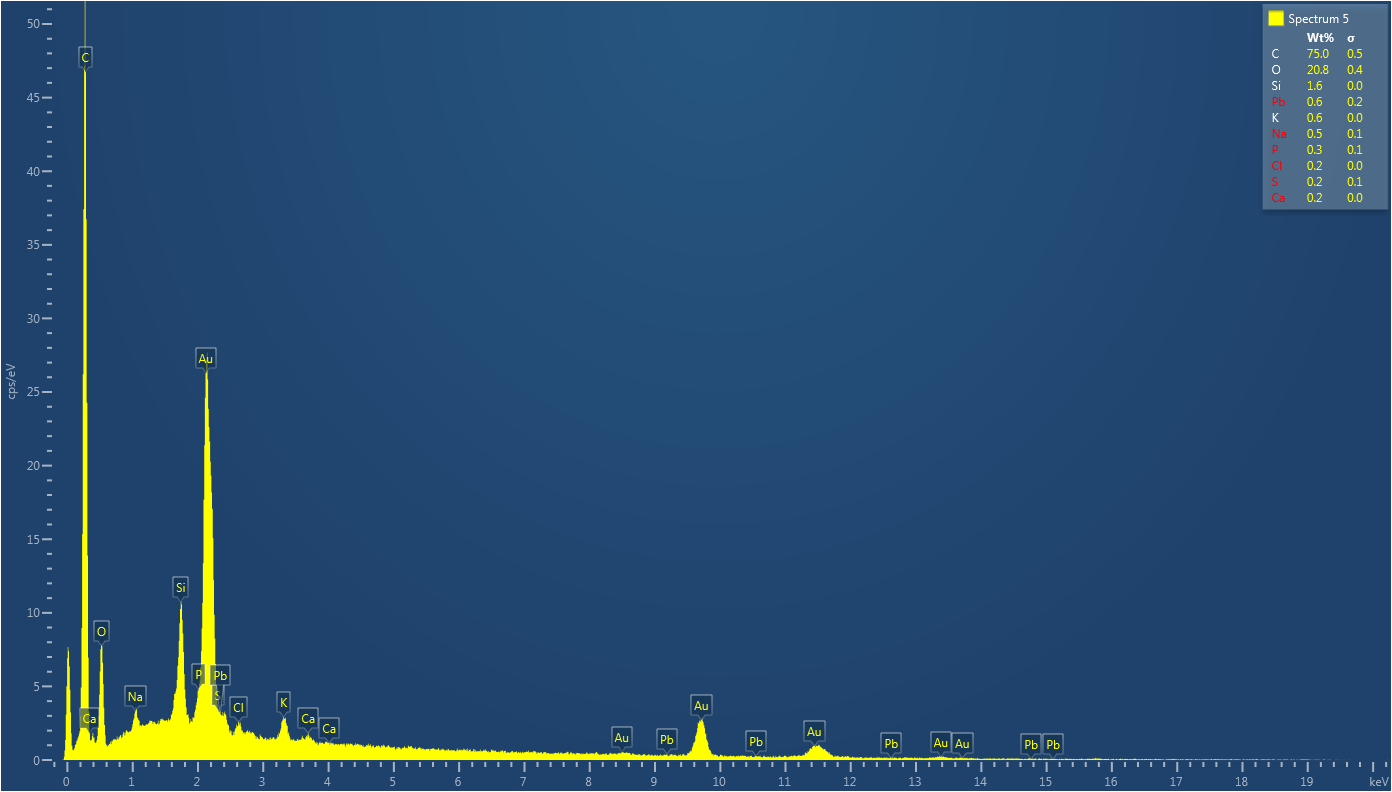

Supplement: S1 Data — (ZIP) [file pone.0337062.s001.zip › EDS/21Jan21/am2_crr_1kx_eds.tiff]

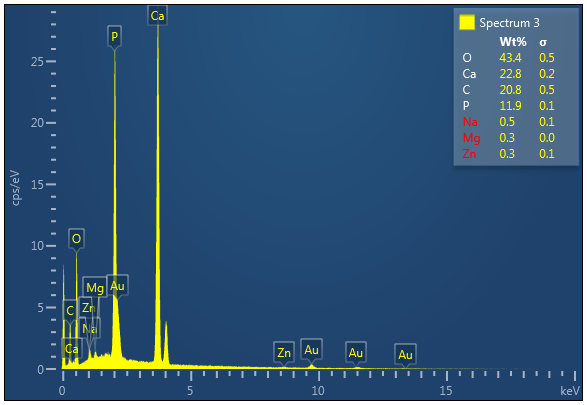

Supplement: S1 Data — (ZIP) [file pone.0337062.s001.zip › EDS/Project 1/reports/Dente higido.docx]

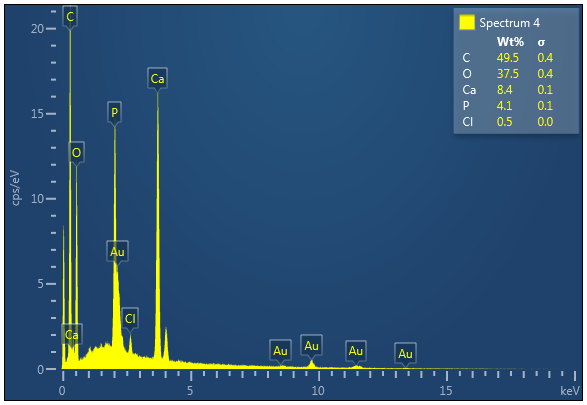

Supplement: S1 Data — (ZIP) [file pone.0337062.s001.zip › EDS/Project 1/reports/CRR 2.docx]

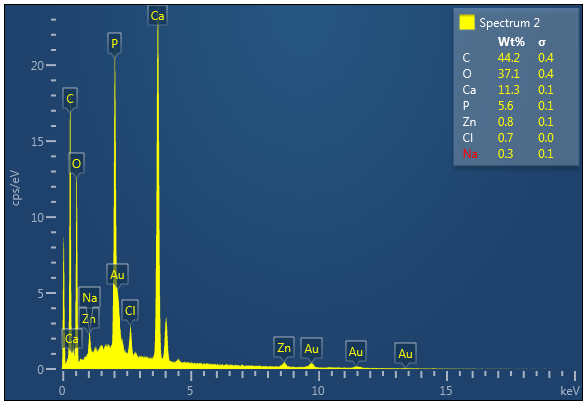

Supplement: S1 Data — (ZIP) [file pone.0337062.s001.zip › EDS/Project 1/reports/CRR.docx]

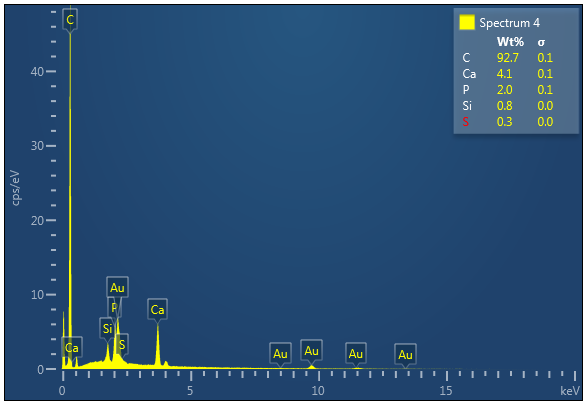

Supplement: S1 Data — (ZIP) [file pone.0337062.s001.zip › EDS/16Abr21/bruna/reports/am3_higido x1k.docx]

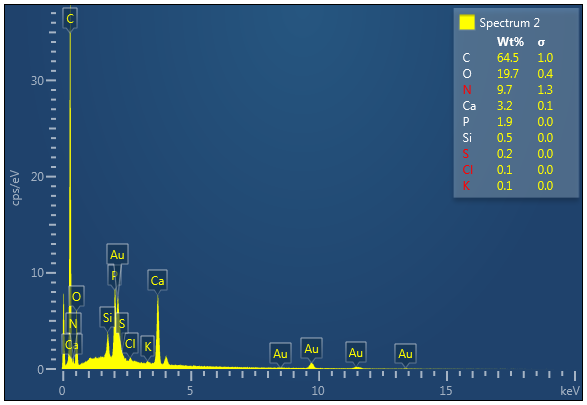

Supplement: S1 Data — (ZIP) [file pone.0337062.s001.zip › EDS/16Abr21/bruna/reports/am1_higido x1k outra regiao.docx]

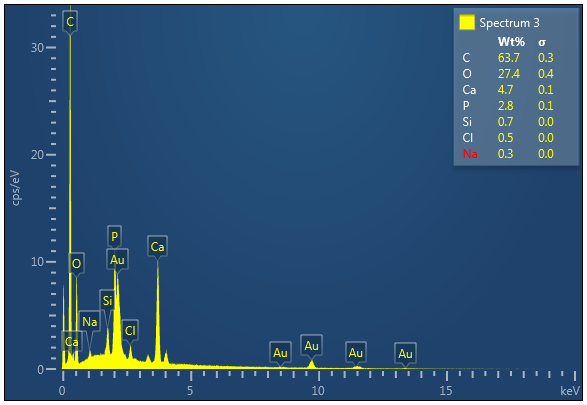

Supplement: S1 Data — (ZIP) [file pone.0337062.s001.zip › EDS/16Abr21/bruna/reports/am2_higido x1k.docx]

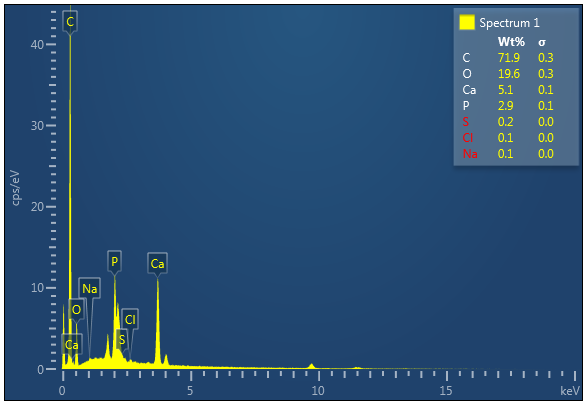

Supplement: S1 Data — (ZIP) [file pone.0337062.s001.zip › EDS/16Abr21/bruna/reports/am1_higido x1k.docx]

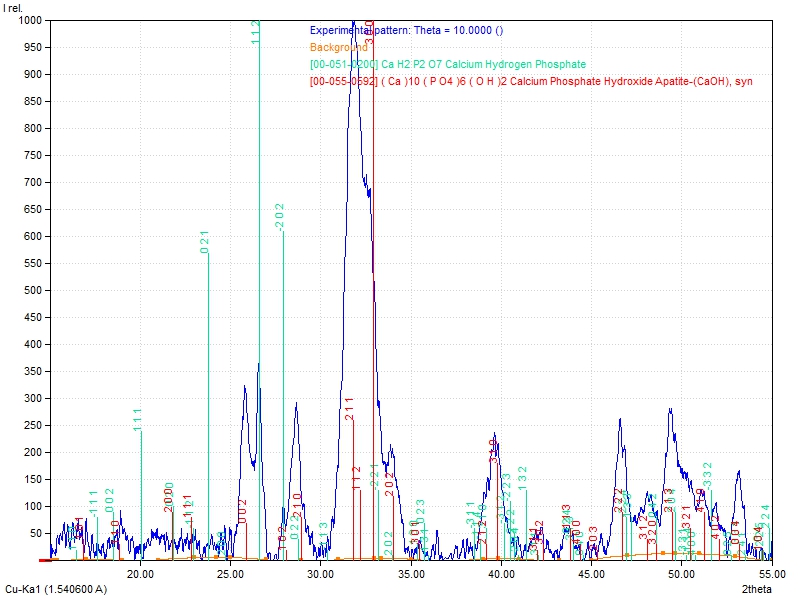

Supplement: S3 Data — (ZIP) [file pone.0337062.s003.zip › BrunaOdo/crr_mesmo/Theta = 10.0000 ()_Pattern.jpg]

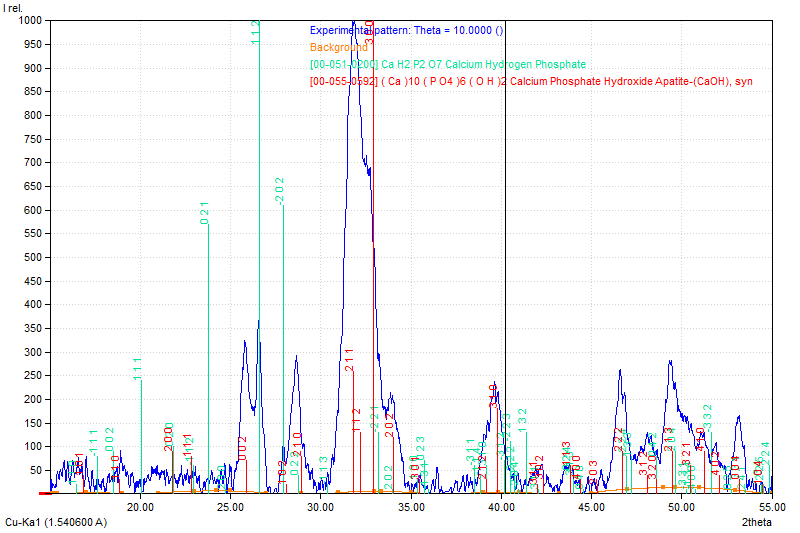

Supplement: S3 Data — (ZIP) [file pone.0337062.s003.zip › BrunaOdo/crr_mesmo/Theta = 10.0000 ()_Report_patterngraphics.png]

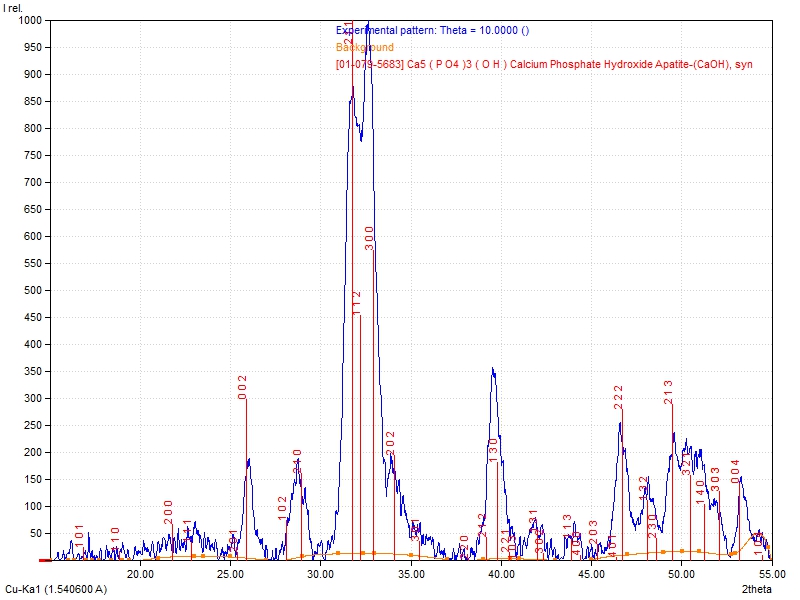

Supplement: S3 Data — (ZIP) [file pone.0337062.s003.zip › BrunaOdo/dentina_irradiada/Theta = 10.0000 ()_Pattern.jpg]

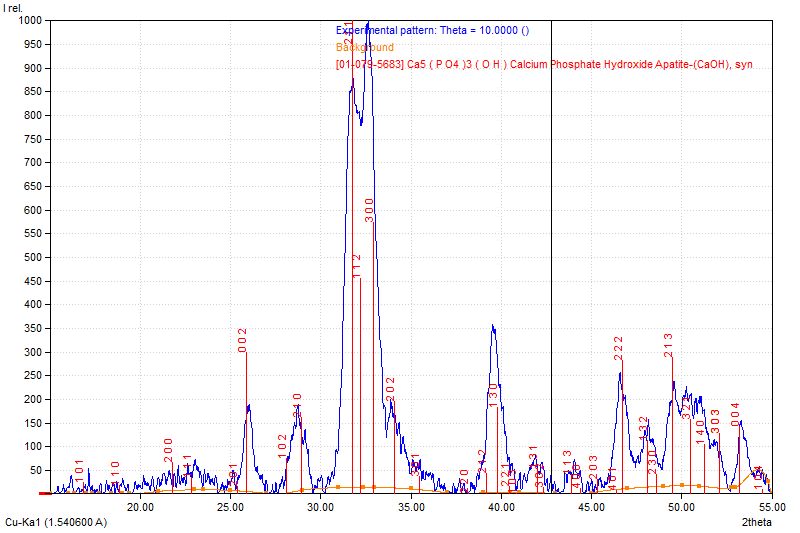

Supplement: S3 Data — (ZIP) [file pone.0337062.s003.zip › BrunaOdo/dentina_irradiada/Theta = 10.0000 ()_Report_patterngraphics.png]

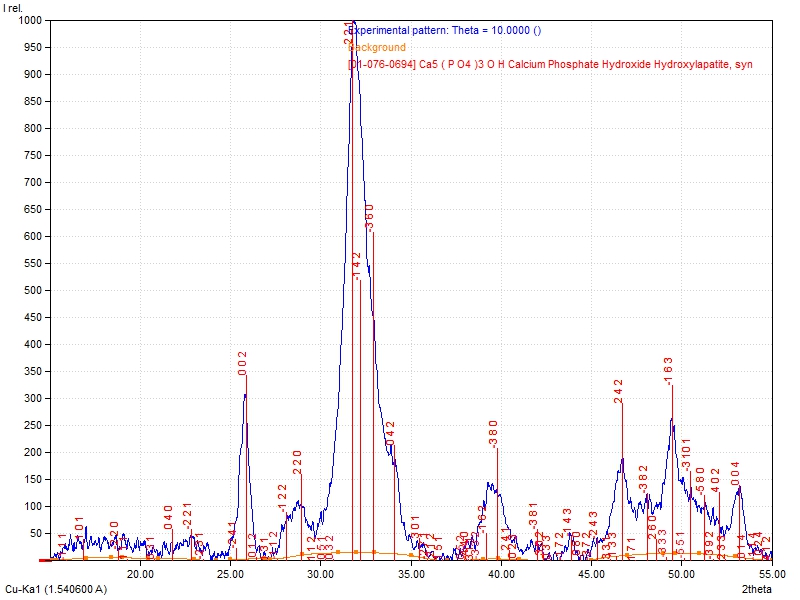

Supplement: S3 Data — (ZIP) [file pone.0337062.s003.zip › BrunaOdo/higido-ds/Theta = 10.0000 ()_Pattern.jpg]

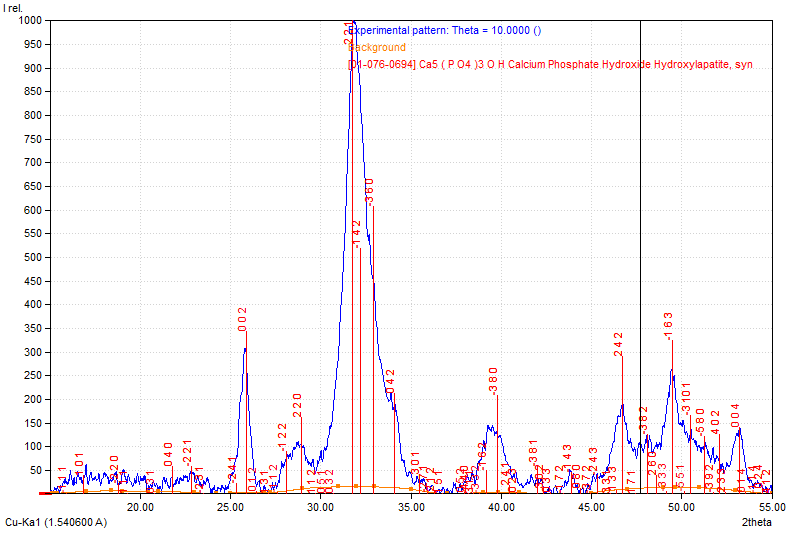

Supplement: S3 Data — (ZIP) [file pone.0337062.s003.zip › BrunaOdo/higido-ds/Theta = 10.0000 ()_Report_patterngraphics.png]

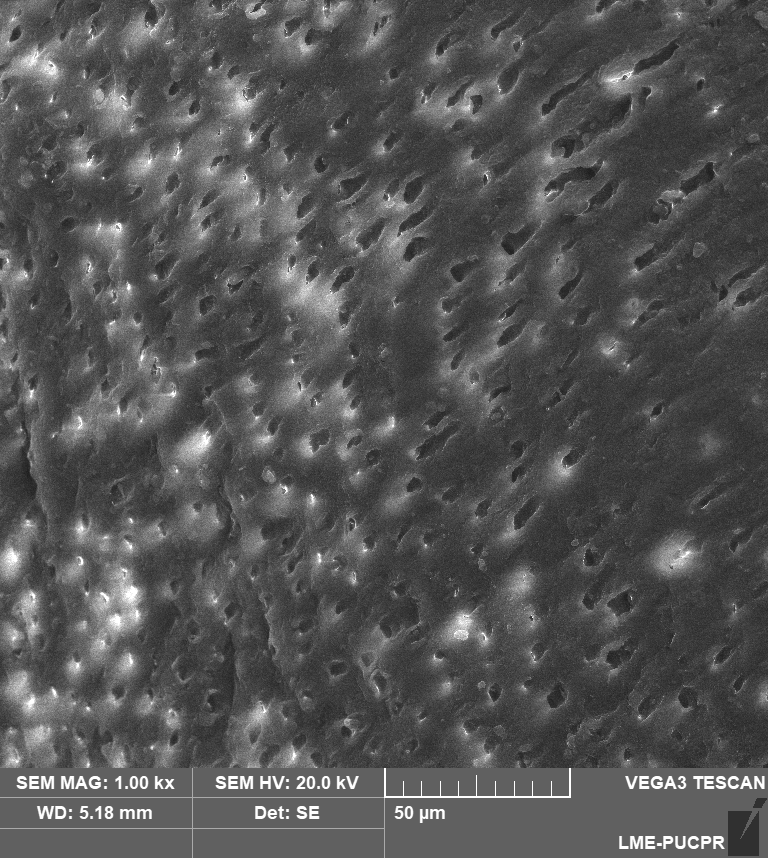

Supplement: S5 Data — (ZIP) [file pone.0337062.s005.zip › SEM/21Jan21/am1/am1_crr_1xk.tif]

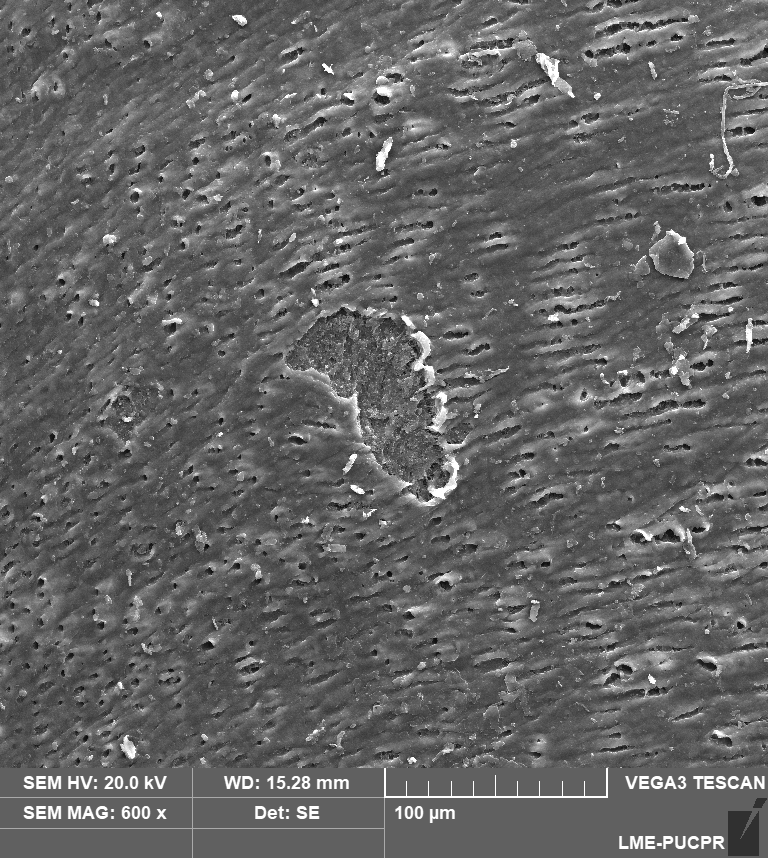

Supplement: S5 Data — (ZIP) [file pone.0337062.s005.zip › SEM/08Nov19/CRR22_600x.tif]

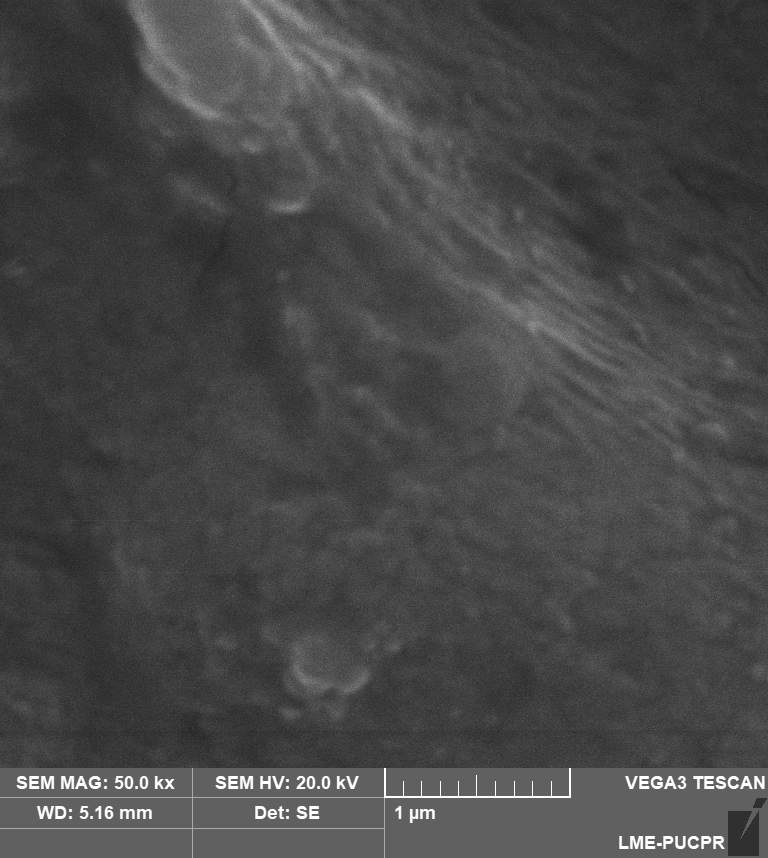

Supplement: S5 Data — (ZIP) [file pone.0337062.s005.zip › SEM/21Jan21/am2/am2_crr_50kx.tif]

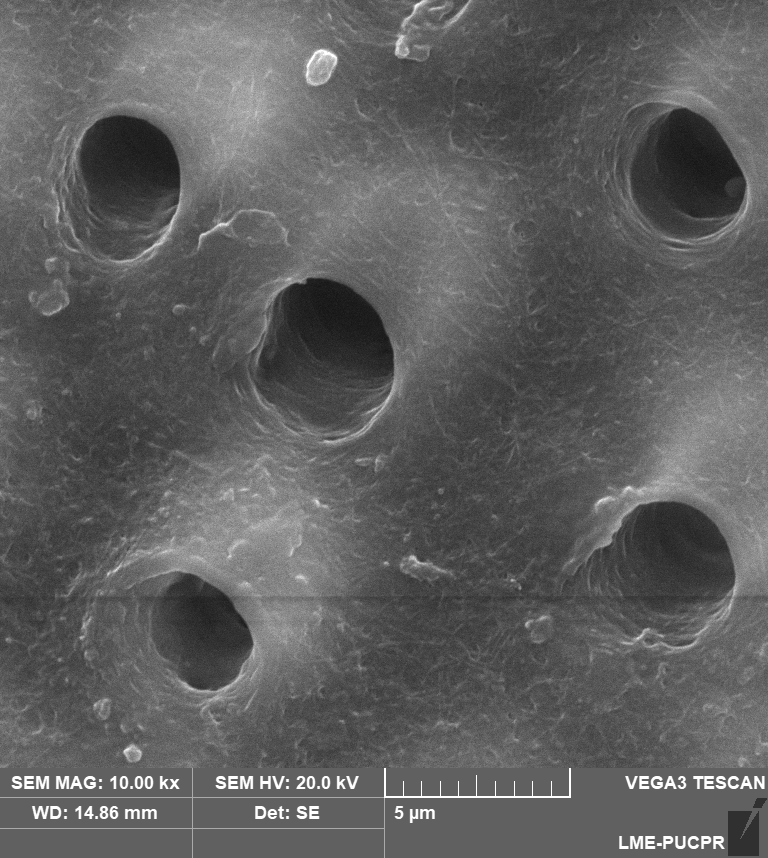

Supplement: S5 Data — (ZIP) [file pone.0337062.s005.zip › SEM/21Jan21/am2/am2_higido_10kx.tif]

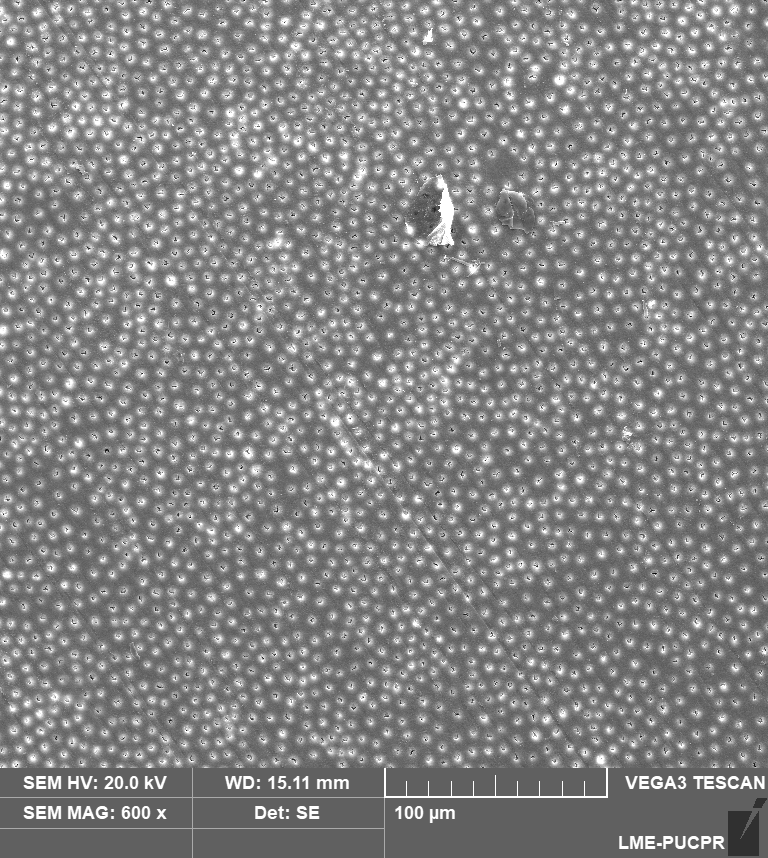

Supplement: S5 Data — (ZIP) [file pone.0337062.s005.zip › SEM/08Nov19/Dente_higido_600x.tif]

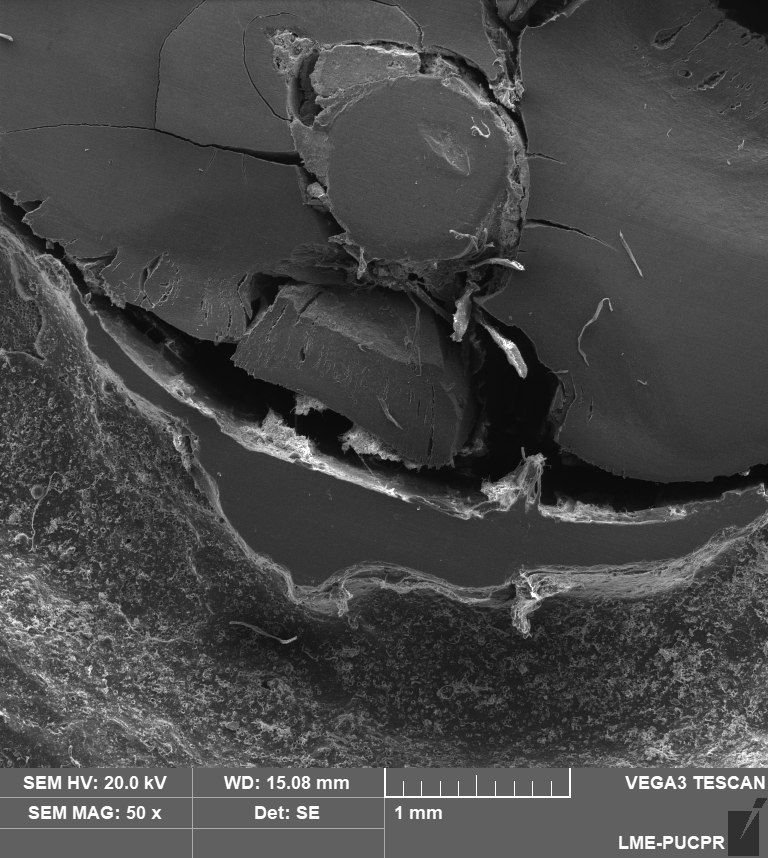

Supplement: S5 Data — (ZIP) [file pone.0337062.s005.zip › SEM/08Nov19/CRR_50x.tif]

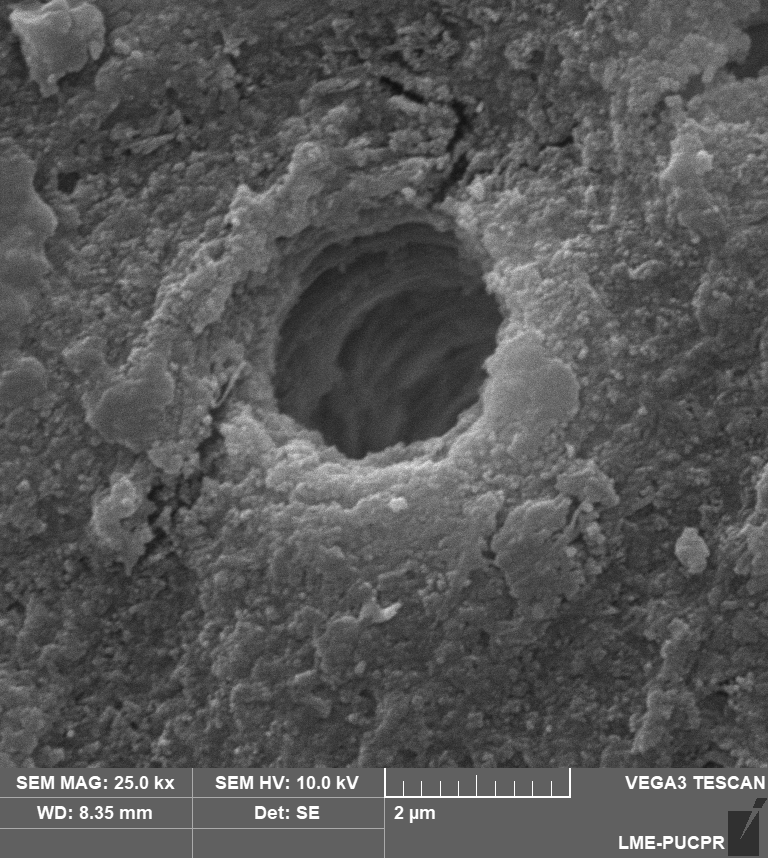

Supplement: S5 Data — (ZIP) [file pone.0337062.s005.zip › SEM/24Set21/irradiado2_x25k2.tif]

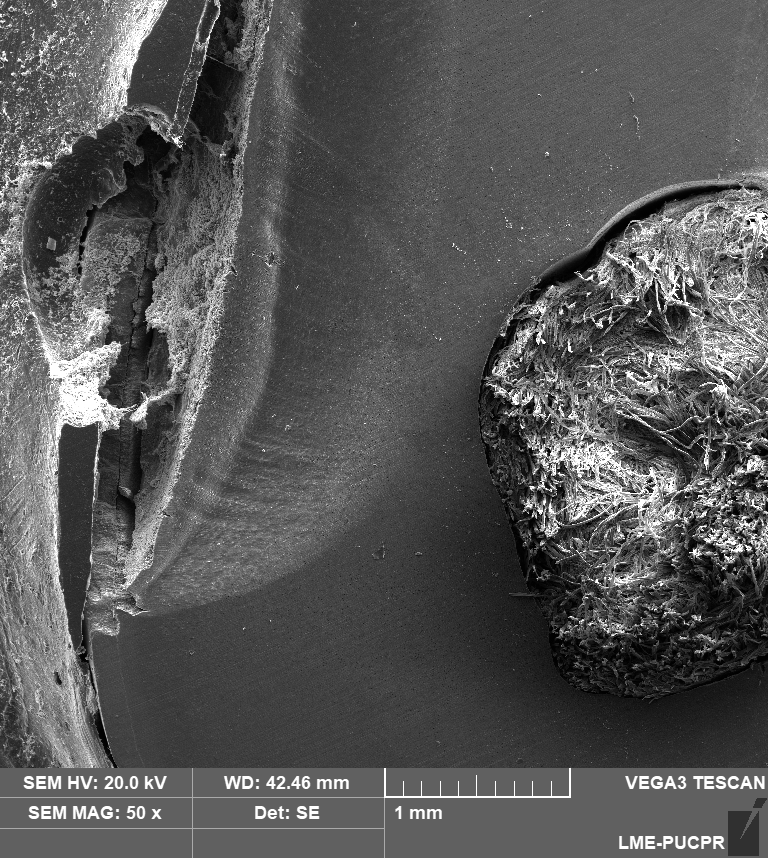

Supplement: S5 Data — (ZIP) [file pone.0337062.s005.zip › SEM/08Nov19/CRR2_50x.tif]

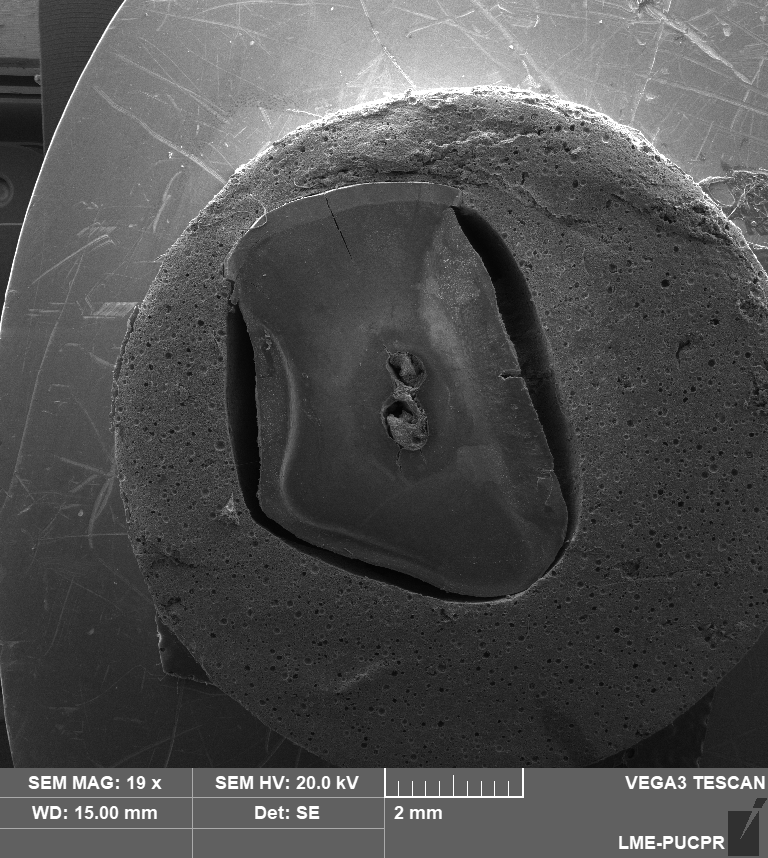

Supplement: S5 Data — (ZIP) [file pone.0337062.s005.zip › SEM/21Jan21/am1/am1_geral_20x.tif]

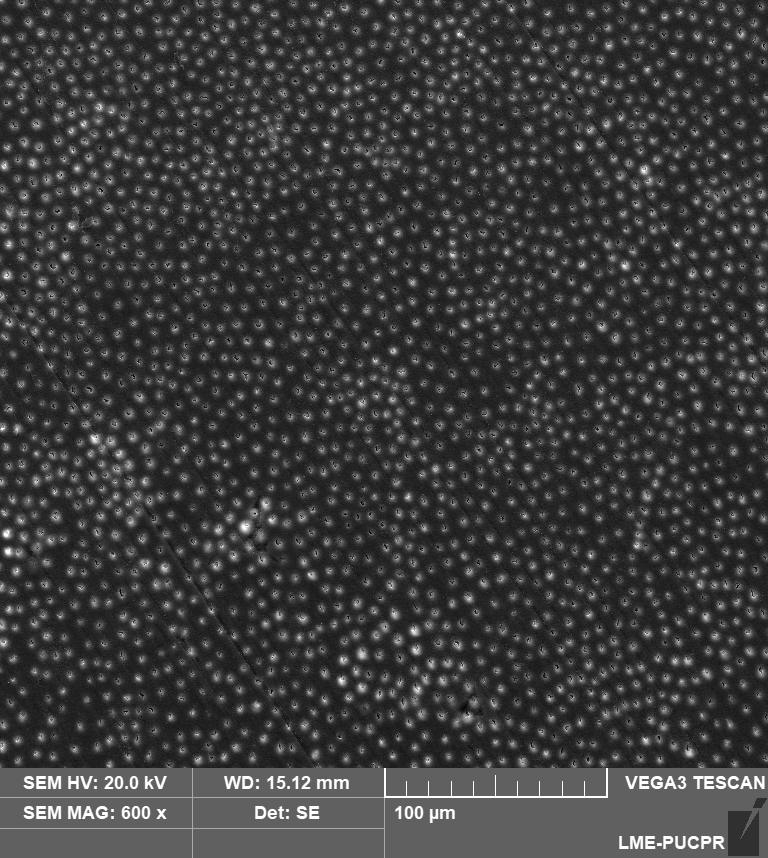

Supplement: S5 Data — (ZIP) [file pone.0337062.s005.zip › SEM/08Nov19/Dente_higido_600x_B.tif]

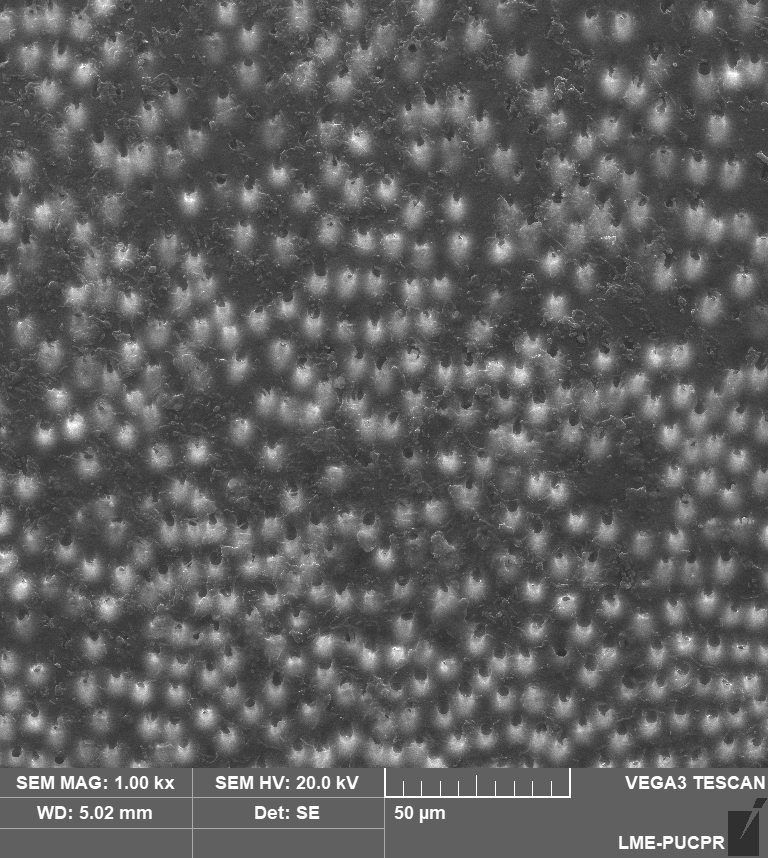

Supplement: S5 Data — (ZIP) [file pone.0337062.s005.zip › SEM/21Jan21/am1/am1_higido_1kx.tif]

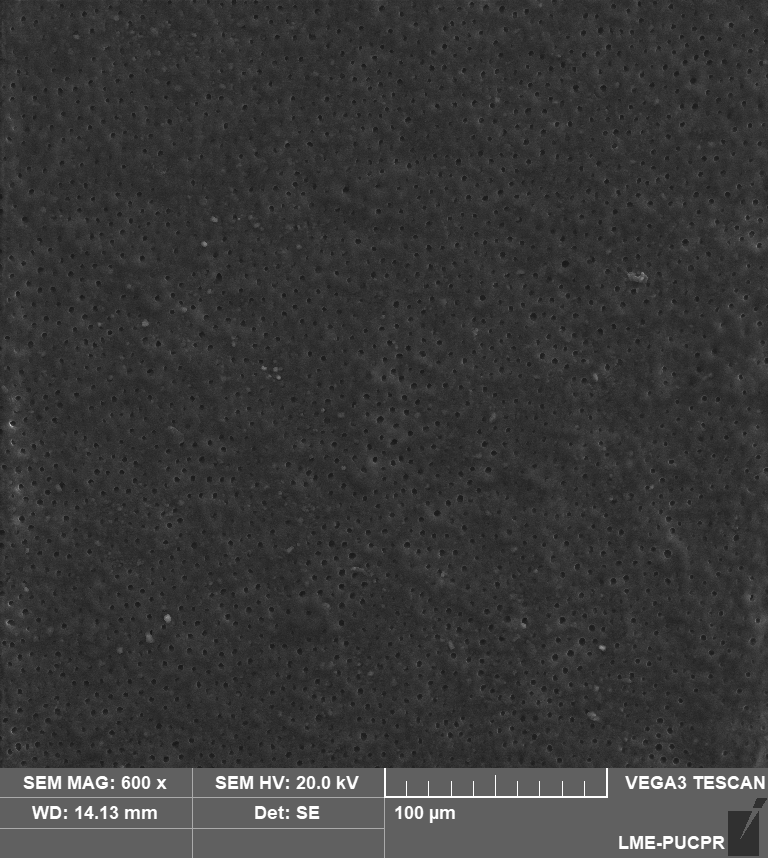

Supplement: S5 Data — (ZIP) [file pone.0337062.s005.zip › SEM/16Abr21/Am higido 3_x600.tif]

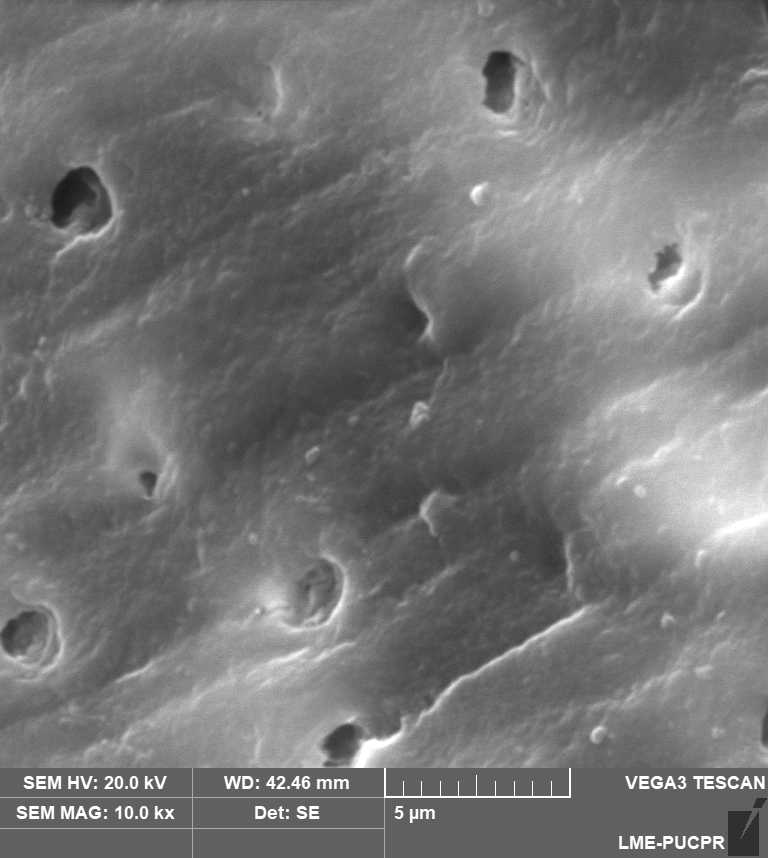

Supplement: S5 Data — (ZIP) [file pone.0337062.s005.zip › SEM/08Nov19/CRR2_10Kx.tif]

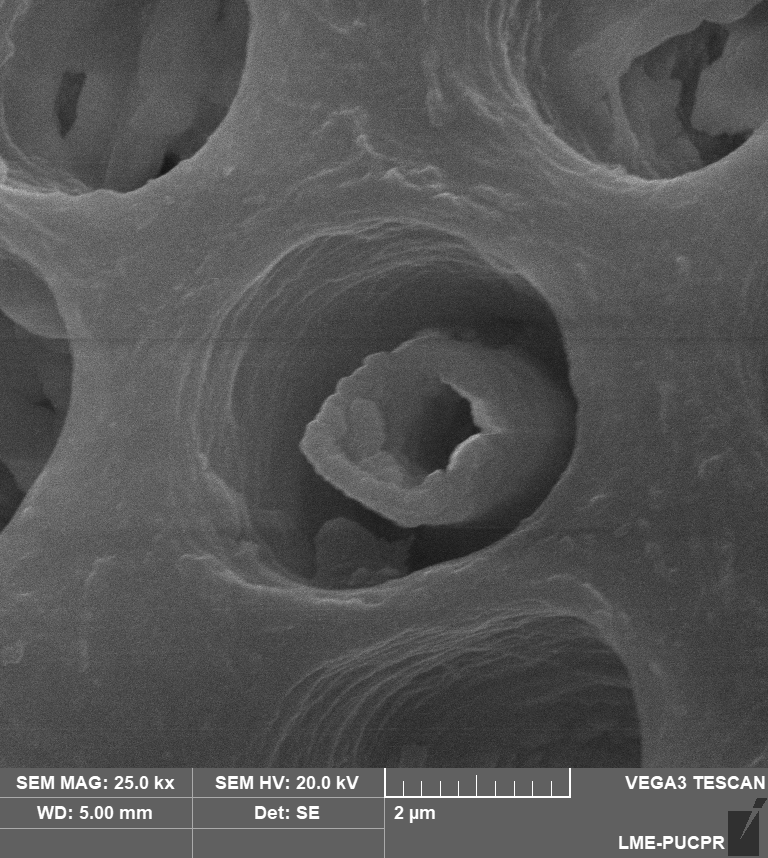

Supplement: S5 Data — (ZIP) [file pone.0337062.s005.zip › SEM/21Jan21/am2/am2_higido_25kx_tubulo.tif]

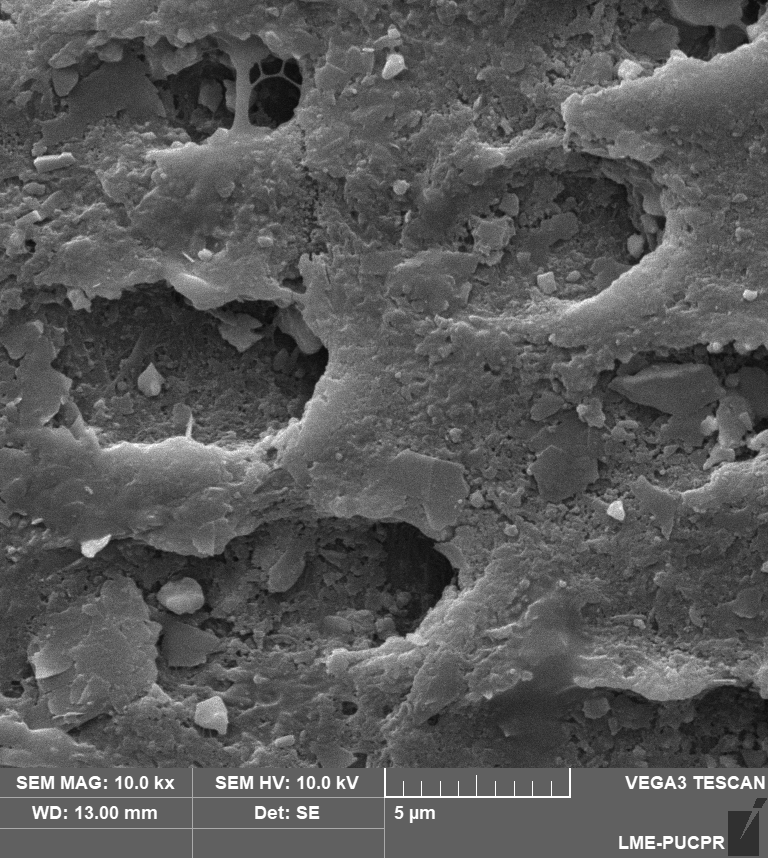

Supplement: S5 Data — (ZIP) [file pone.0337062.s005.zip › SEM/24Set21/crr_x10k.tif]

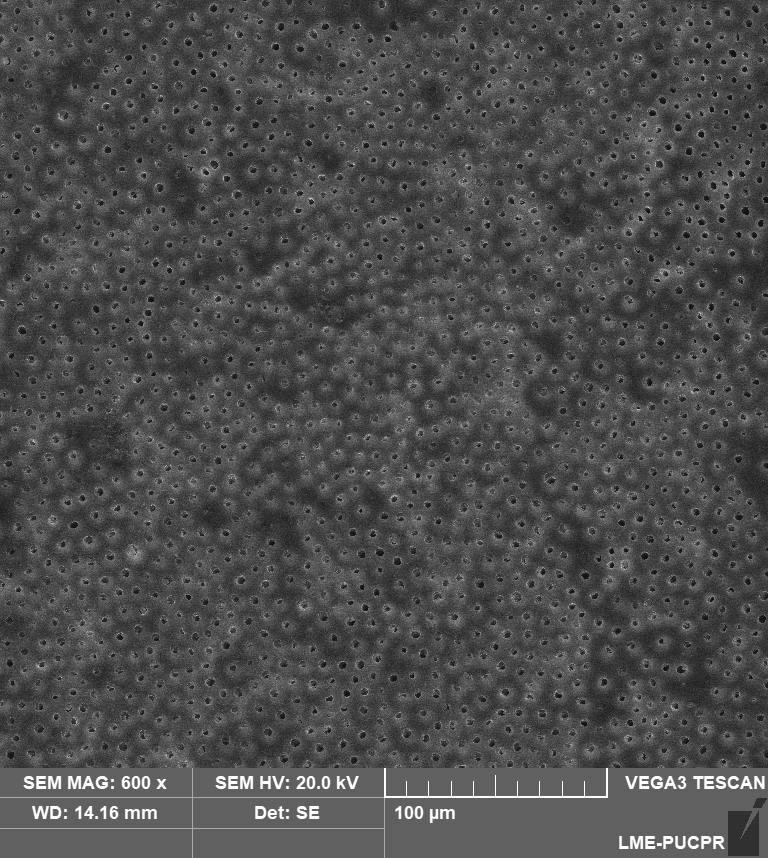

Supplement: S5 Data — (ZIP) [file pone.0337062.s005.zip › SEM/16Abr21/Am higido 2_x600.tif]

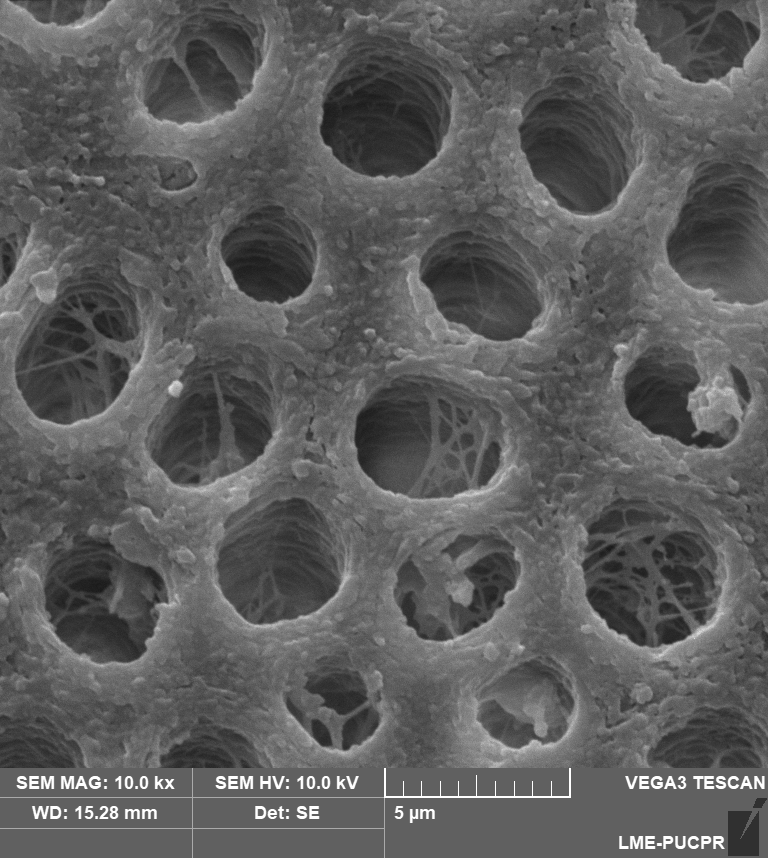

Supplement: S5 Data — (ZIP) [file pone.0337062.s005.zip › SEM/24Set21/higido_x10k.tif]

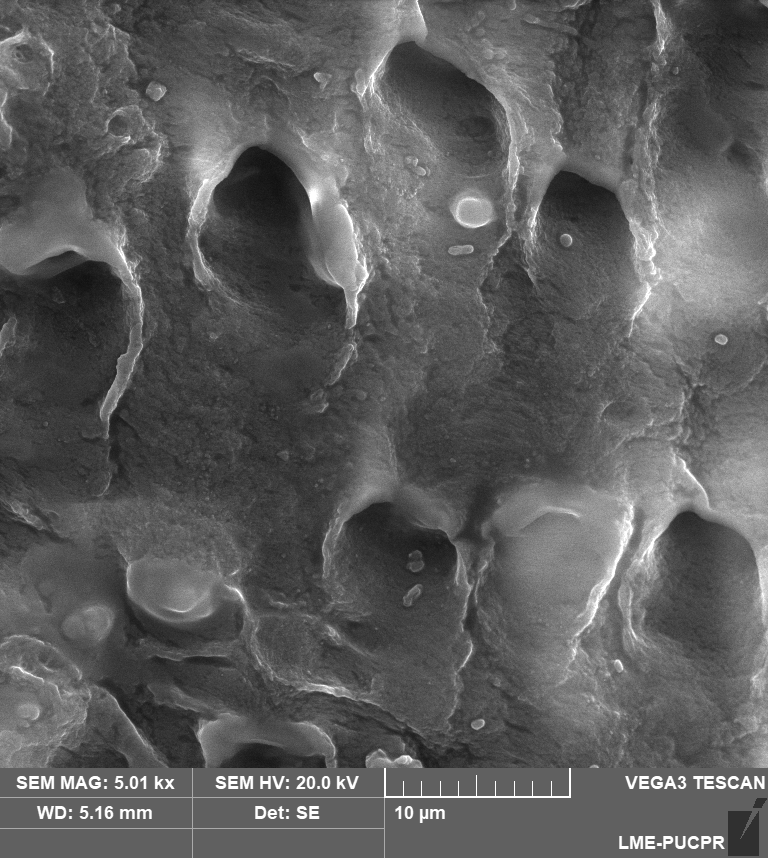

Supplement: S5 Data — (ZIP) [file pone.0337062.s005.zip › SEM/21Jan21/am2/am2_crr_5kx.tif]

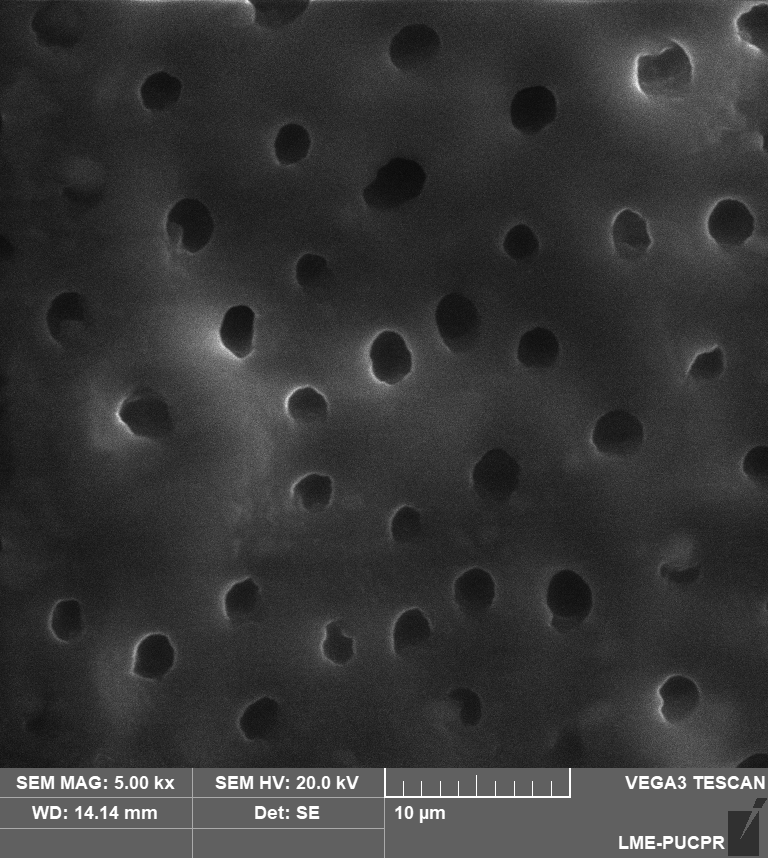

Supplement: S5 Data — (ZIP) [file pone.0337062.s005.zip › SEM/16Abr21/Am higido 3_x5k.tif]

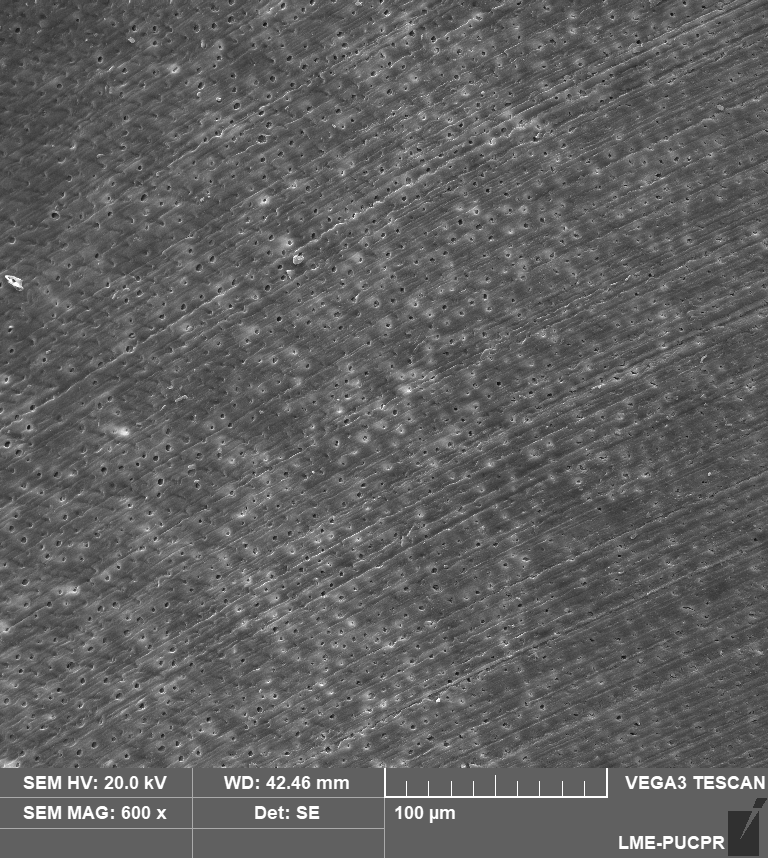

Supplement: S5 Data — (ZIP) [file pone.0337062.s005.zip › SEM/08Nov19/CRR2_600x.tif]

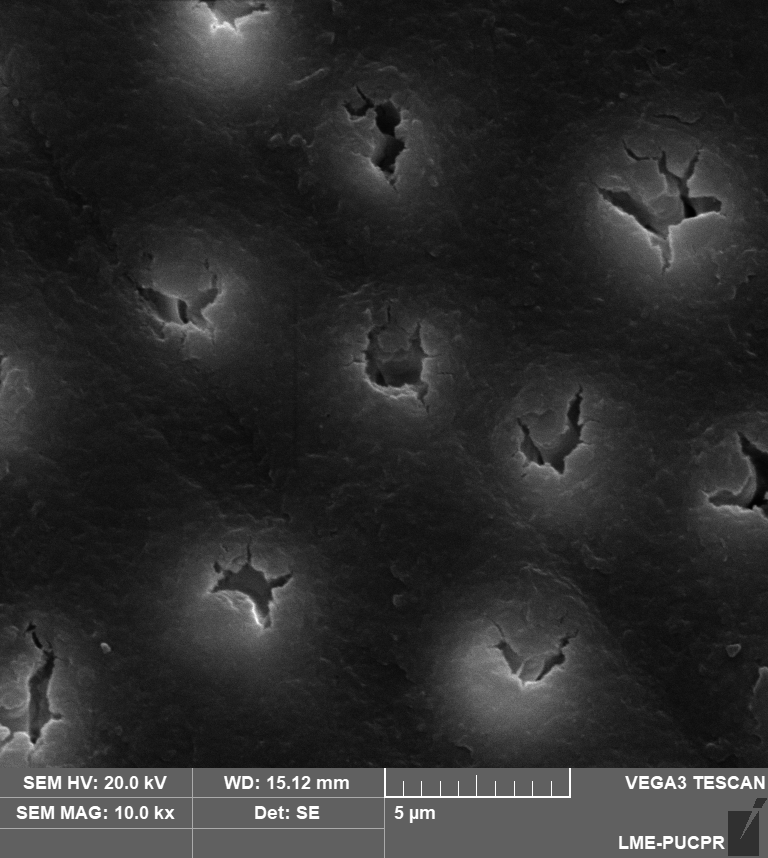

Supplement: S5 Data — (ZIP) [file pone.0337062.s005.zip › SEM/08Nov19/Dente_higido_10Kx_A.tif]

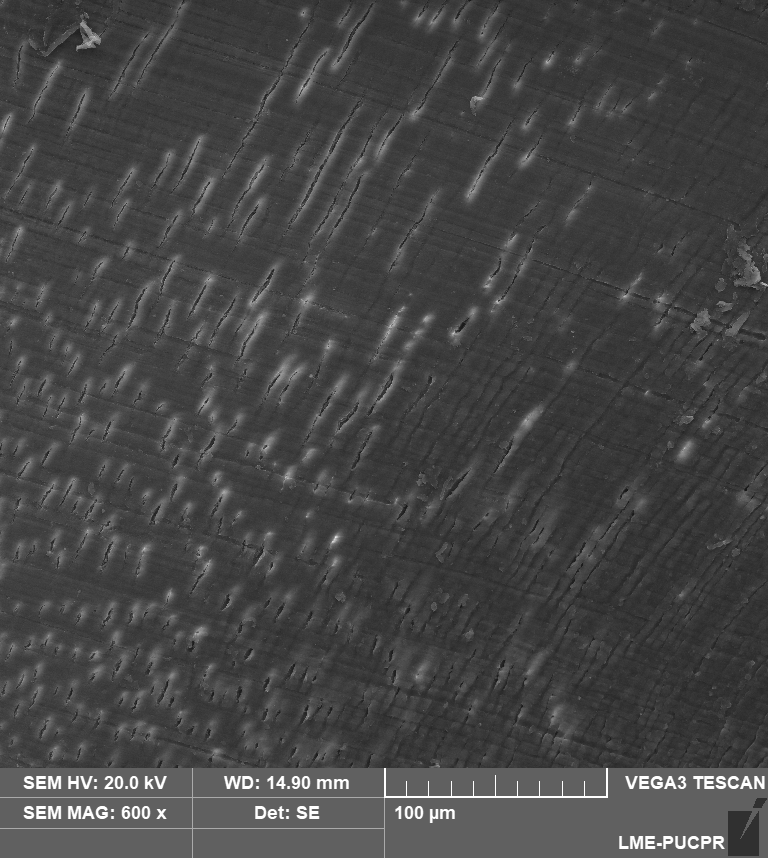

Supplement: S5 Data — (ZIP) [file pone.0337062.s005.zip › SEM/08Nov19/CRR_600x_B.tif]

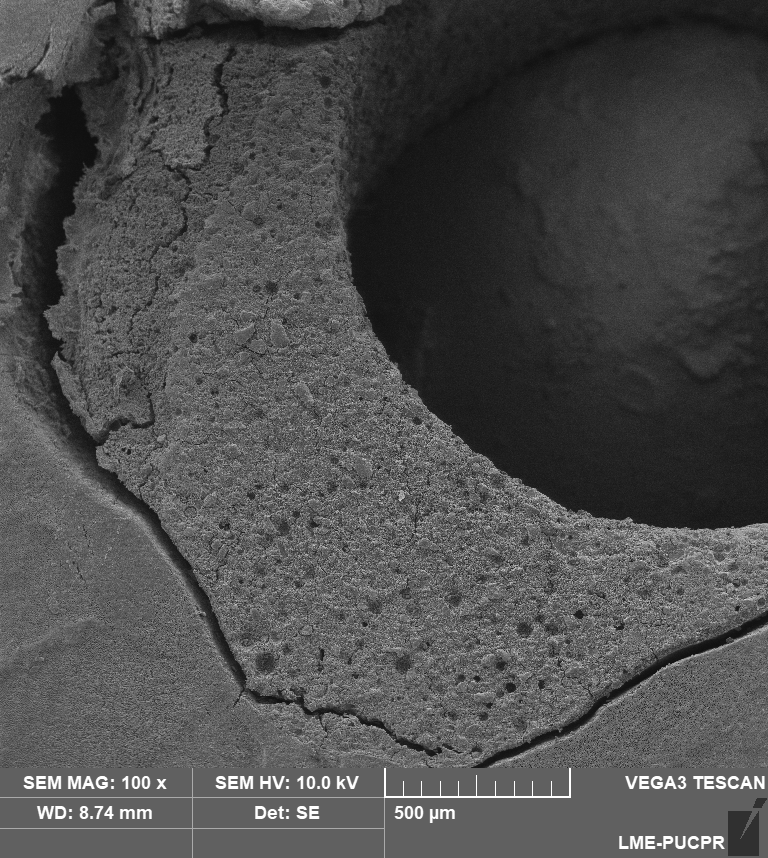

Supplement: S5 Data — (ZIP) [file pone.0337062.s005.zip › SEM/24Set21/irradiado3_x100.tif]

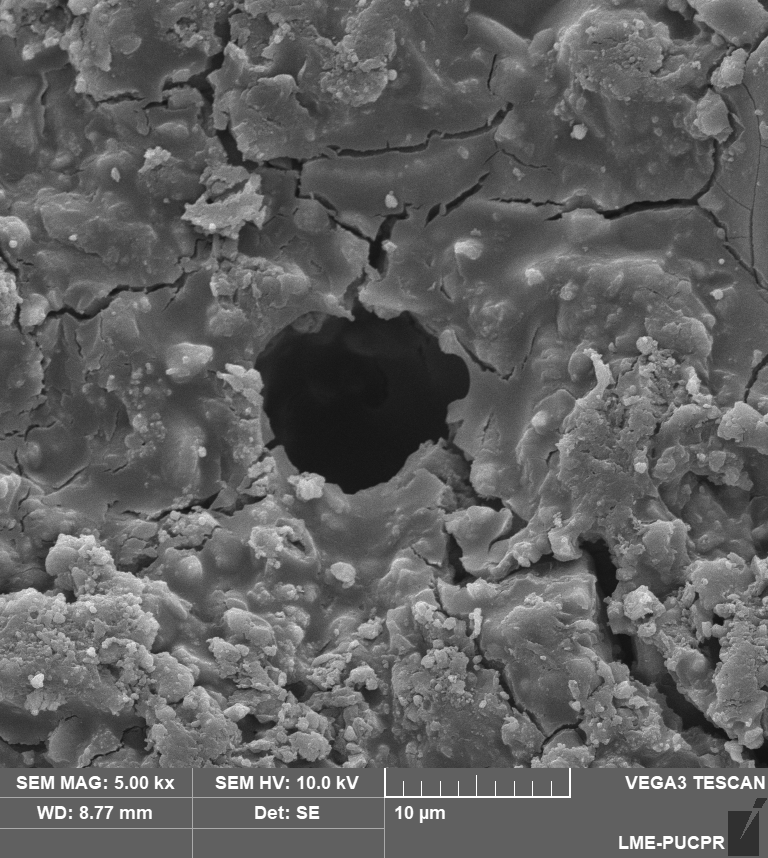

Supplement: S5 Data — (ZIP) [file pone.0337062.s005.zip › SEM/24Set21/irradiado3_x5k.tif]

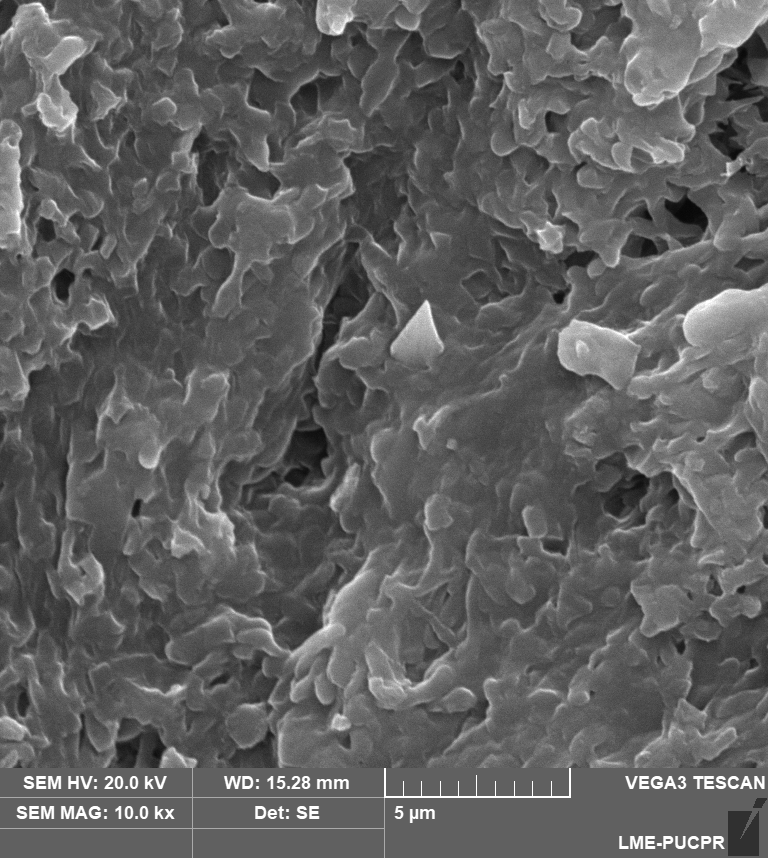

Supplement: S5 Data — (ZIP) [file pone.0337062.s005.zip › SEM/08Nov19/CRR22_10kx.tif]

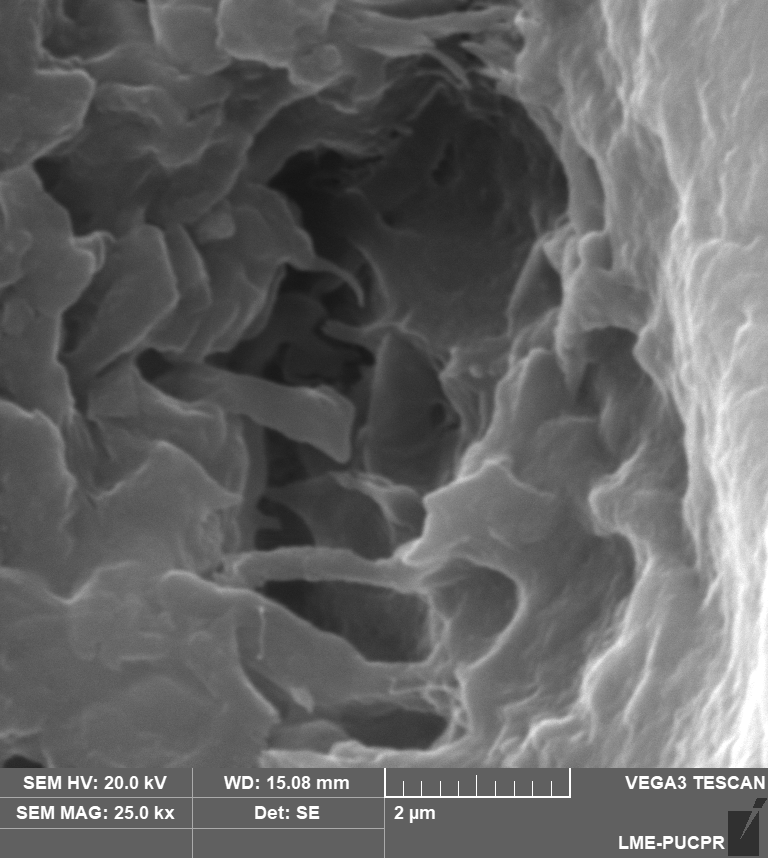

Supplement: S5 Data — (ZIP) [file pone.0337062.s005.zip › SEM/08Nov19/CRR_25Kx.tif]

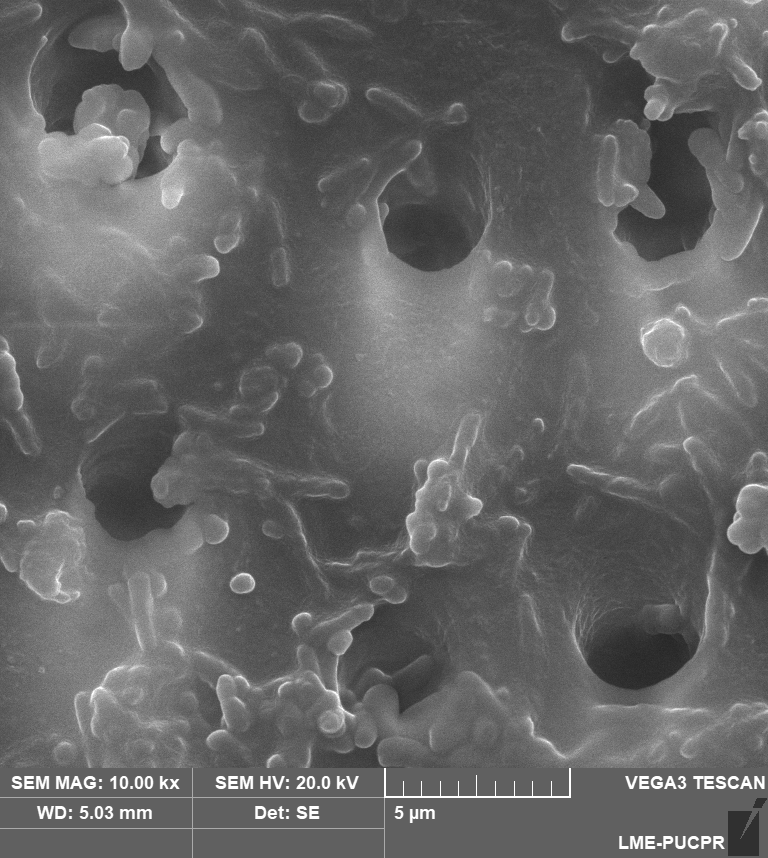

Supplement: S5 Data — (ZIP) [file pone.0337062.s005.zip › SEM/21Jan21/am1/am1_higido_10kx.tif]

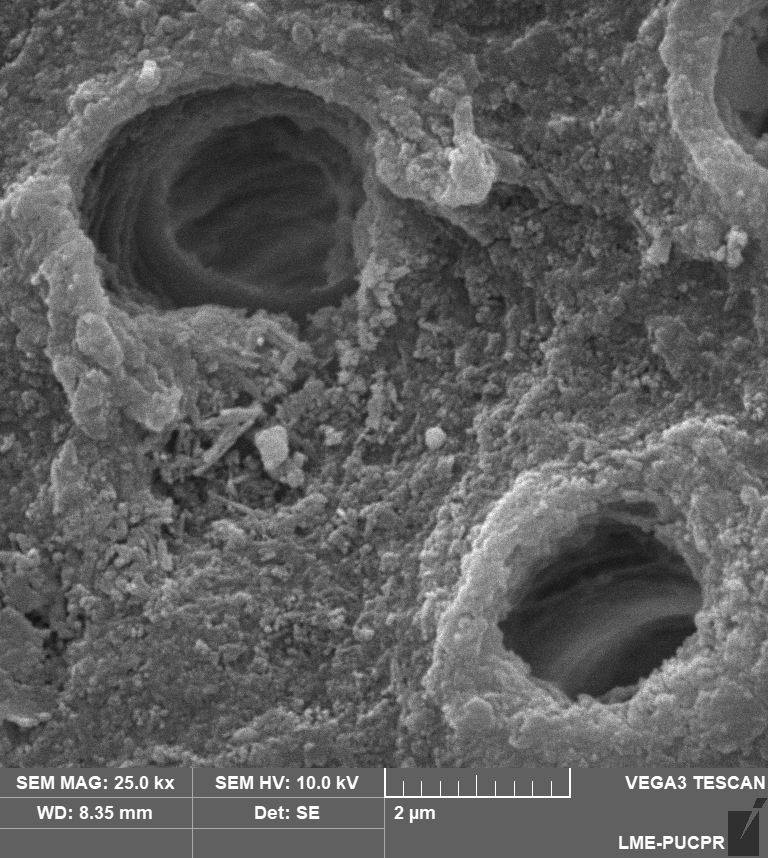

Supplement: S5 Data — (ZIP) [file pone.0337062.s005.zip › SEM/24Set21/irradiado2_x25k.tif]

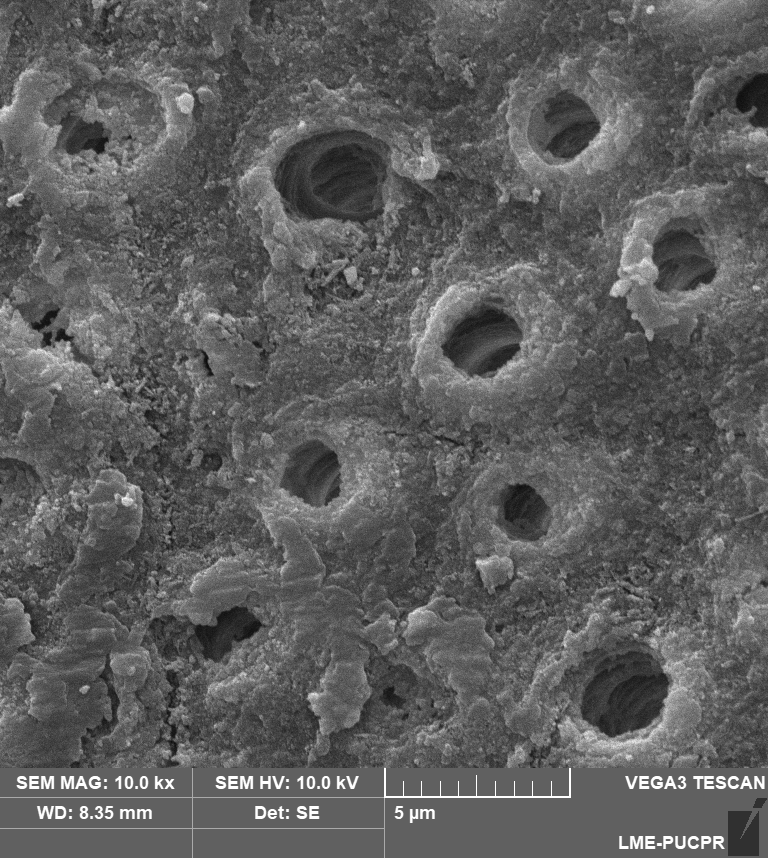

Supplement: S5 Data — (ZIP) [file pone.0337062.s005.zip › SEM/24Set21/irradiado2_x10k.tif]

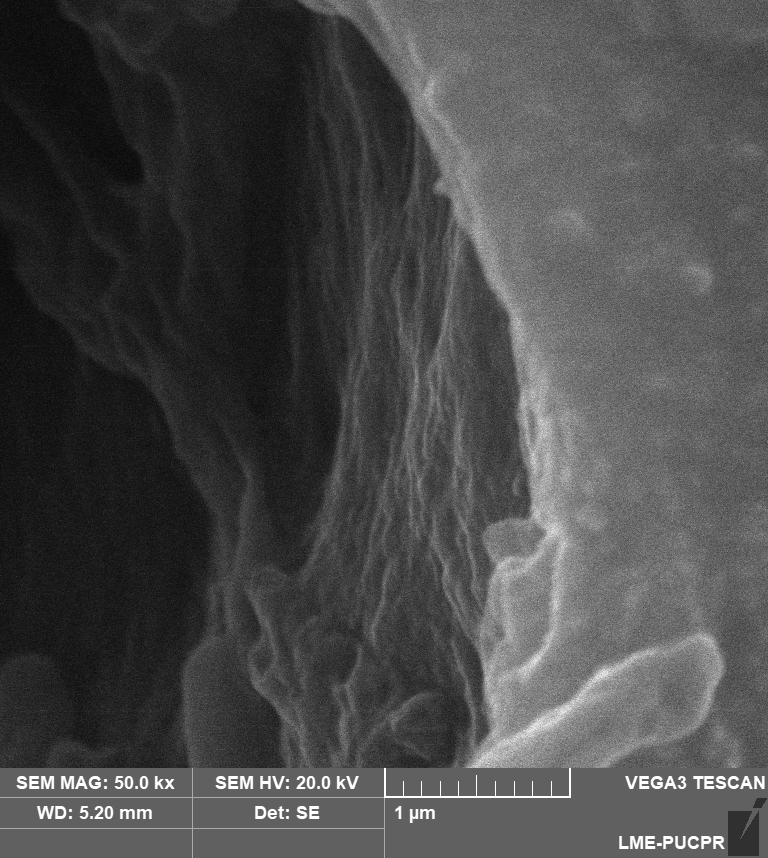

Supplement: S5 Data — (ZIP) [file pone.0337062.s005.zip › SEM/21Jan21/am1/am1_crr_50xka.tif]

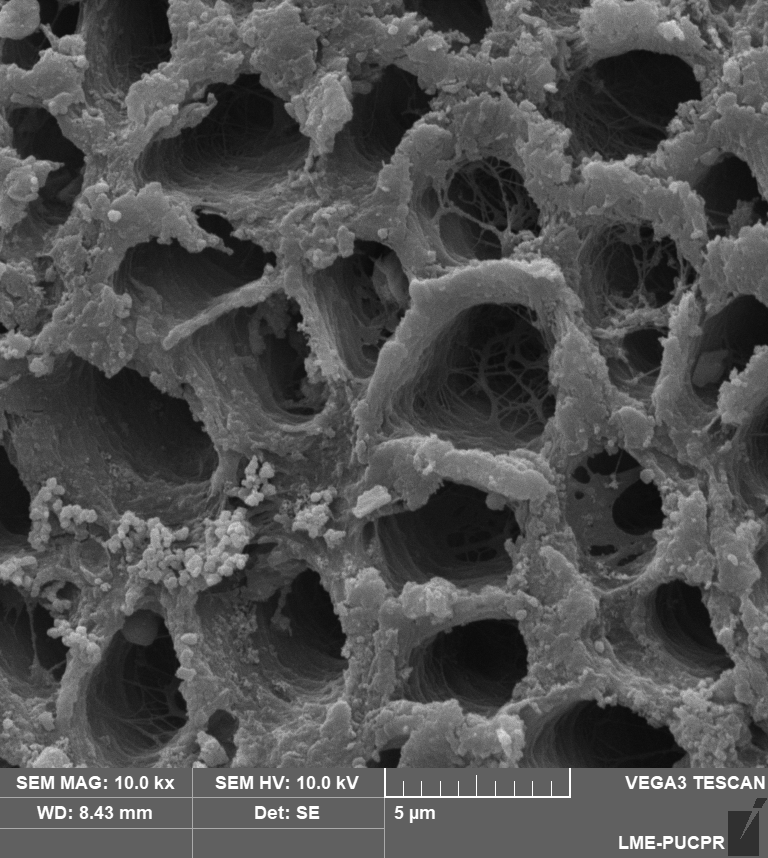

Supplement: S5 Data — (ZIP) [file pone.0337062.s005.zip › SEM/24Set21/irradiado_x105k.tif]

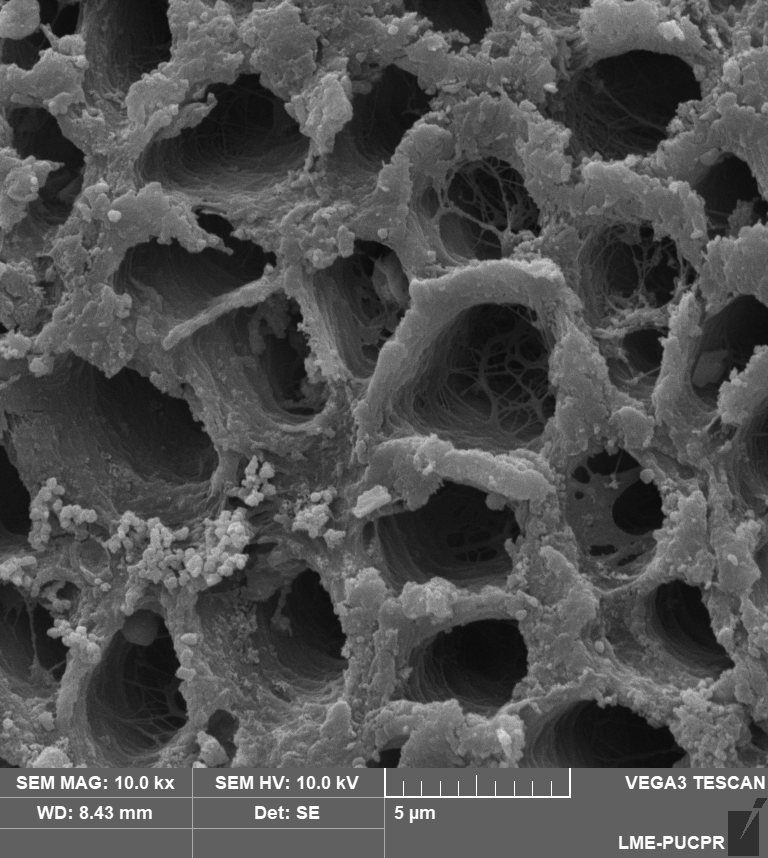

Supplement: S5 Data — (ZIP) [file pone.0337062.s005.zip › SEM/24Set21/irradiado_x10k.tif]

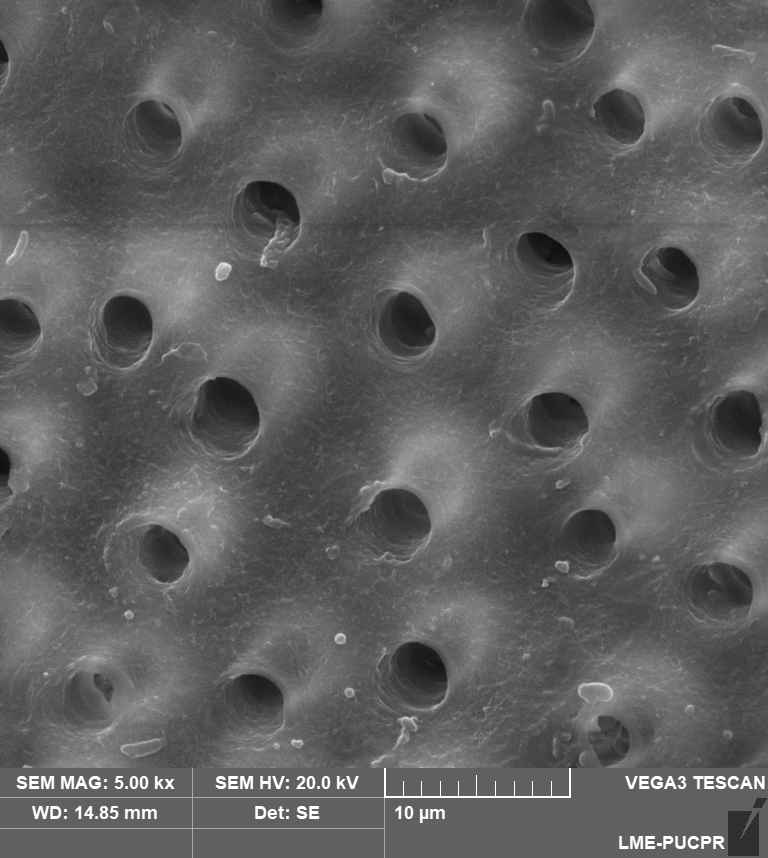

Supplement: S5 Data — (ZIP) [file pone.0337062.s005.zip › SEM/21Jan21/am2/am2_higido_5kx.tif]

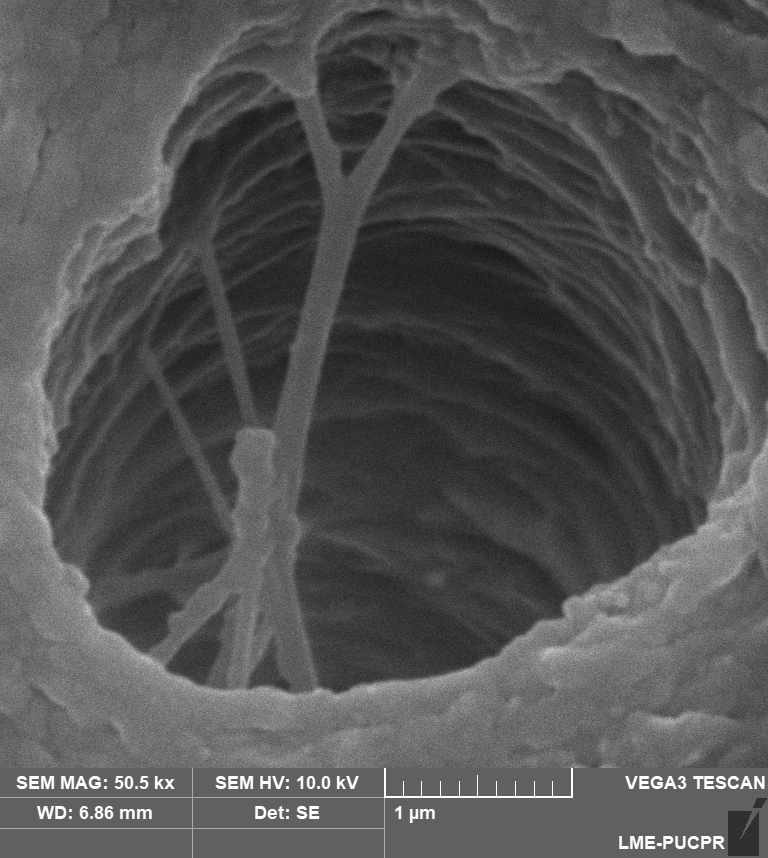

Supplement: S5 Data — (ZIP) [file pone.0337062.s005.zip › SEM/24Set21/higido_x50k tilt 55.tif]

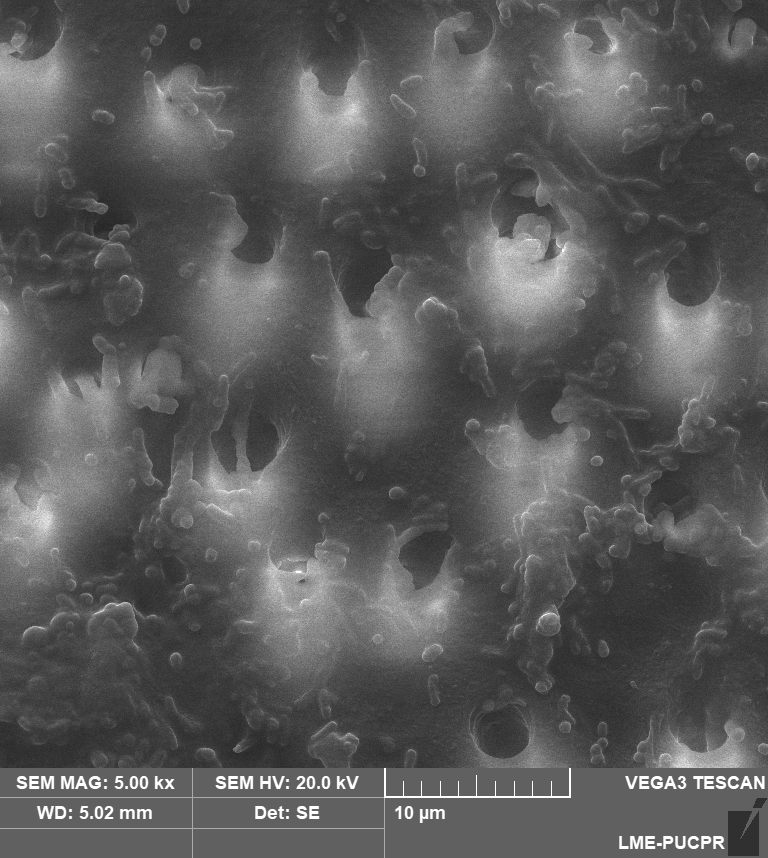

Supplement: S5 Data — (ZIP) [file pone.0337062.s005.zip › SEM/21Jan21/am1/am1_higido_5kx.tif]

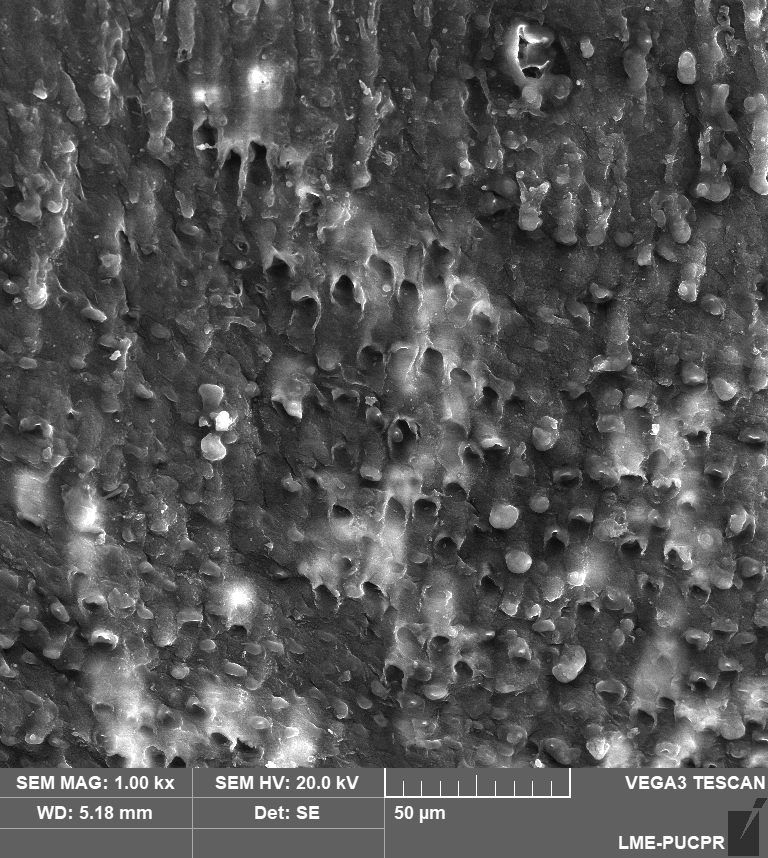

Supplement: S5 Data — (ZIP) [file pone.0337062.s005.zip › SEM/21Jan21/am2/am2_crr_1kx.tif]

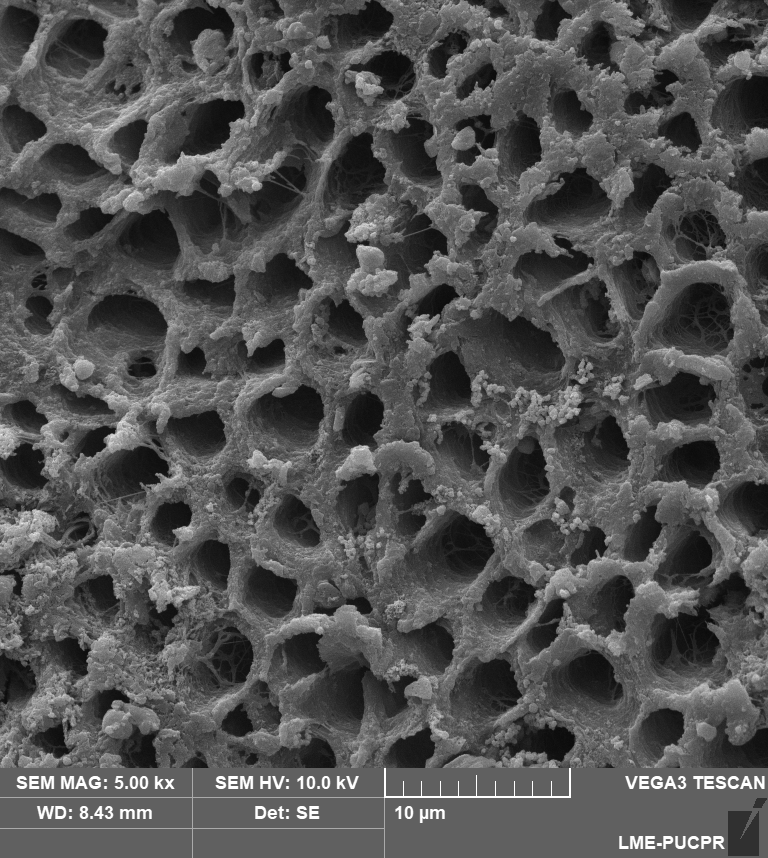

Supplement: S5 Data — (ZIP) [file pone.0337062.s005.zip › SEM/24Set21/irradiado_x5k.tif]

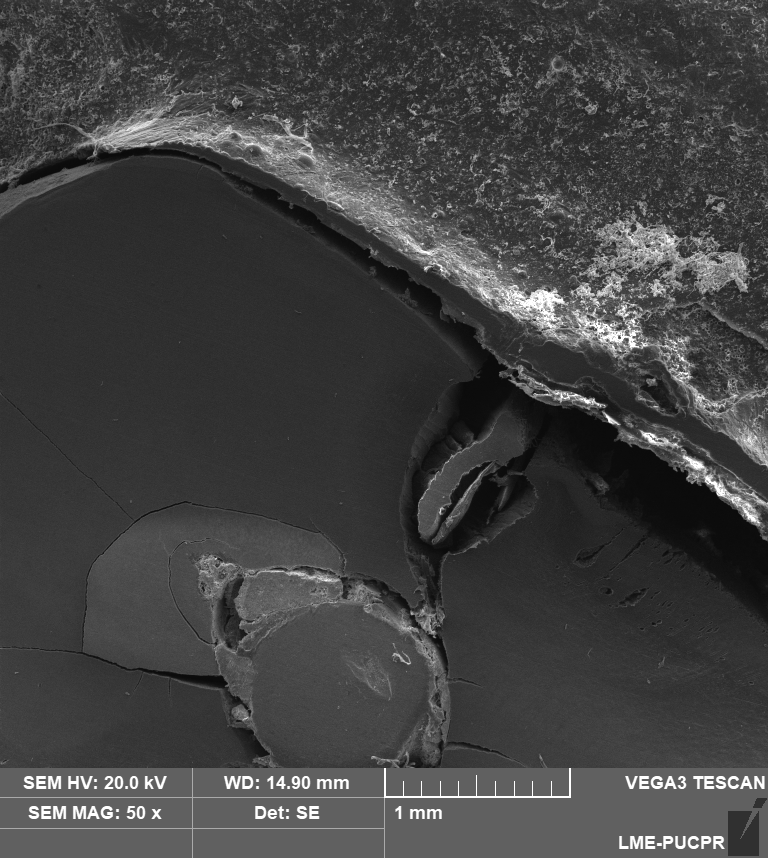

Supplement: S5 Data — (ZIP) [file pone.0337062.s005.zip › SEM/08Nov19/CRR_50x_B.tif]

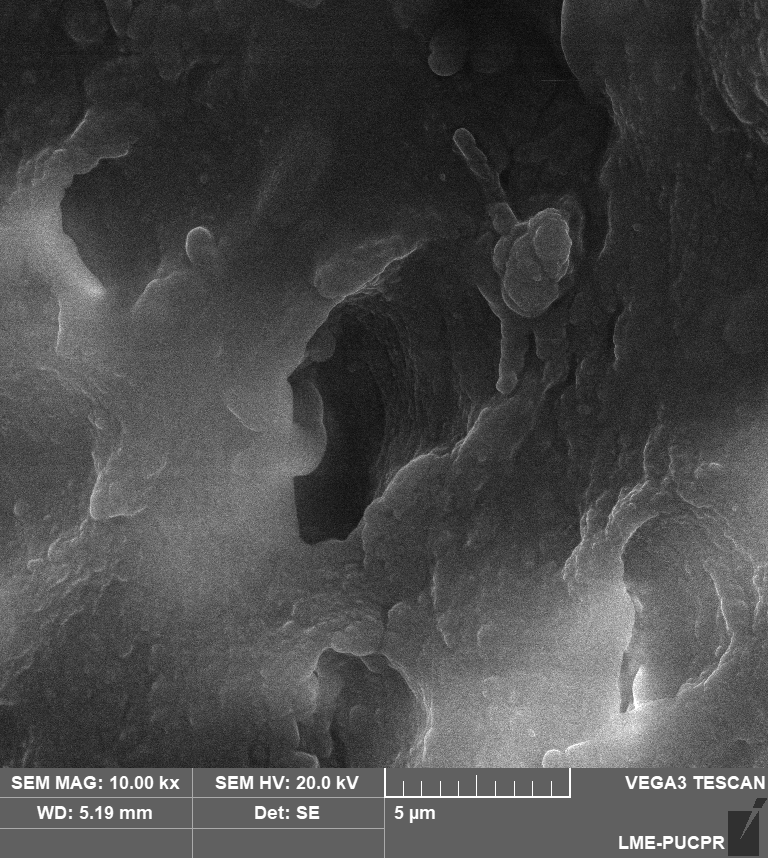

Supplement: S5 Data — (ZIP) [file pone.0337062.s005.zip › SEM/21Jan21/am1/am1_crr_10kx.tif]

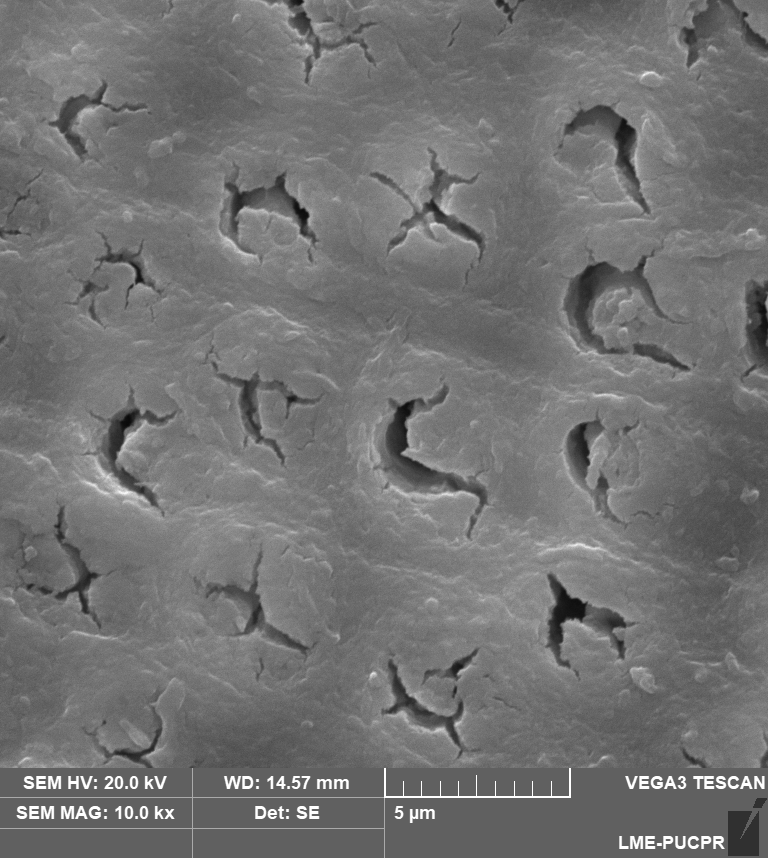

Supplement: S5 Data — (ZIP) [file pone.0337062.s005.zip › SEM/08Nov19/Dente_higido2_10Kx.tif]

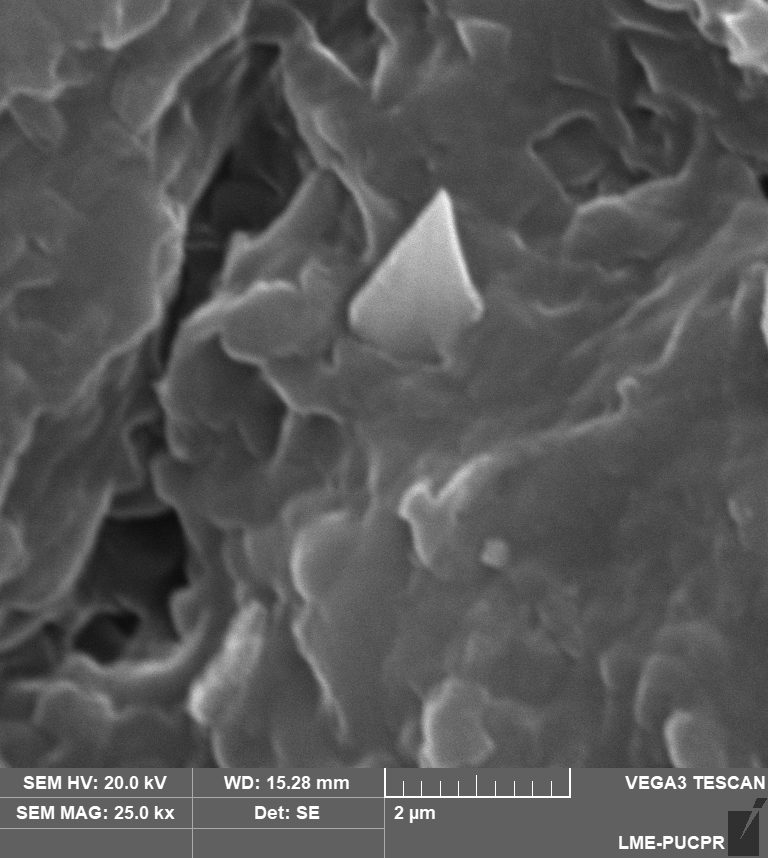

Supplement: S5 Data — (ZIP) [file pone.0337062.s005.zip › SEM/08Nov19/CRR22_25kx.tif]

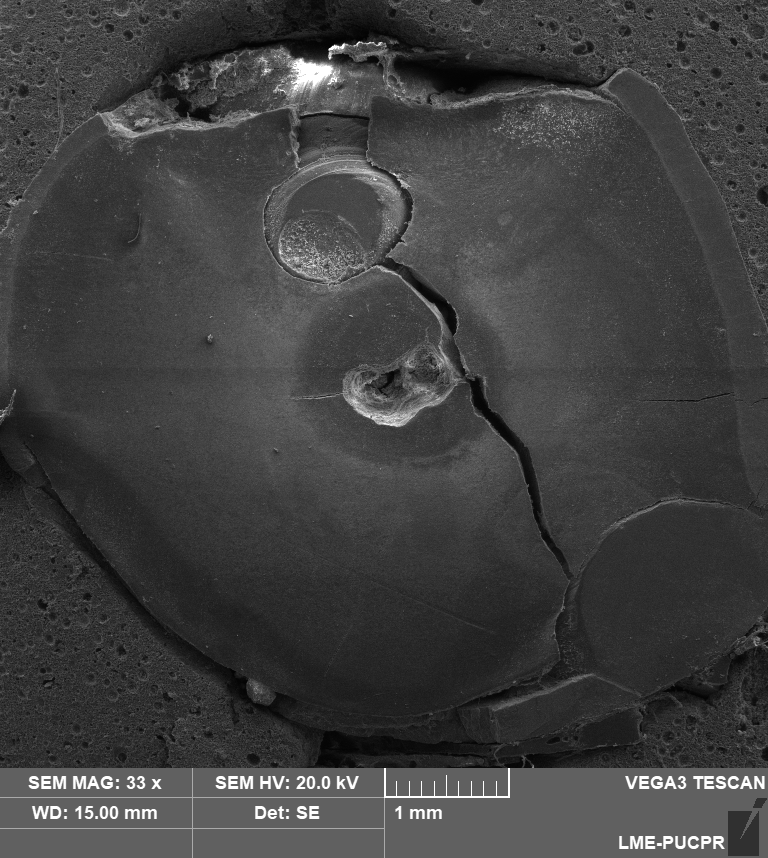

Supplement: S5 Data — (ZIP) [file pone.0337062.s005.zip › SEM/21Jan21/am1/am2_geral_33x.tif]

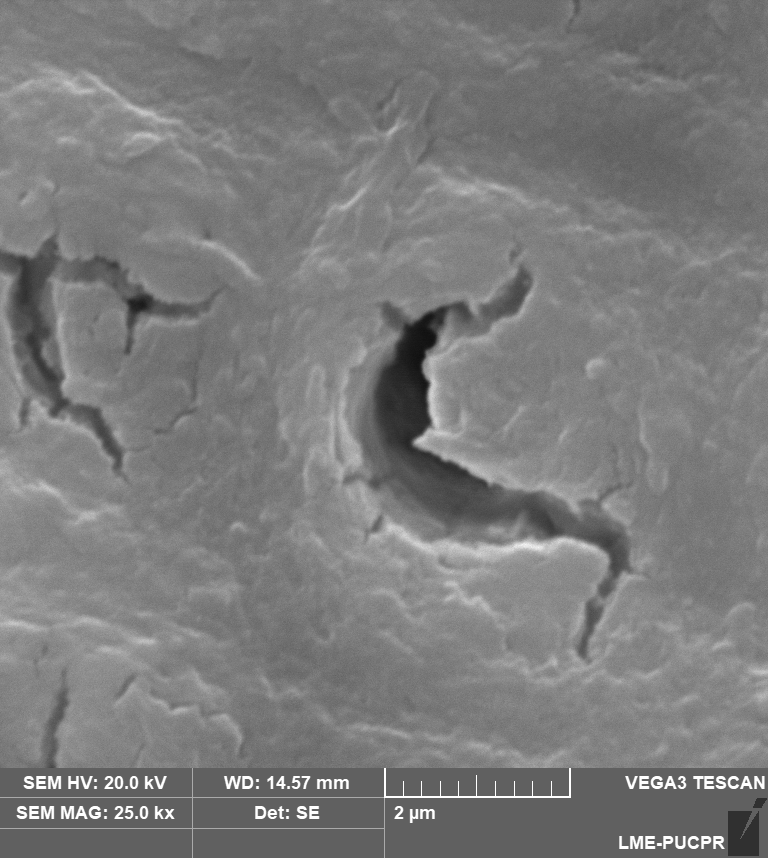

Supplement: S5 Data — (ZIP) [file pone.0337062.s005.zip › SEM/08Nov19/Dente_higido2_25Kx.tif]

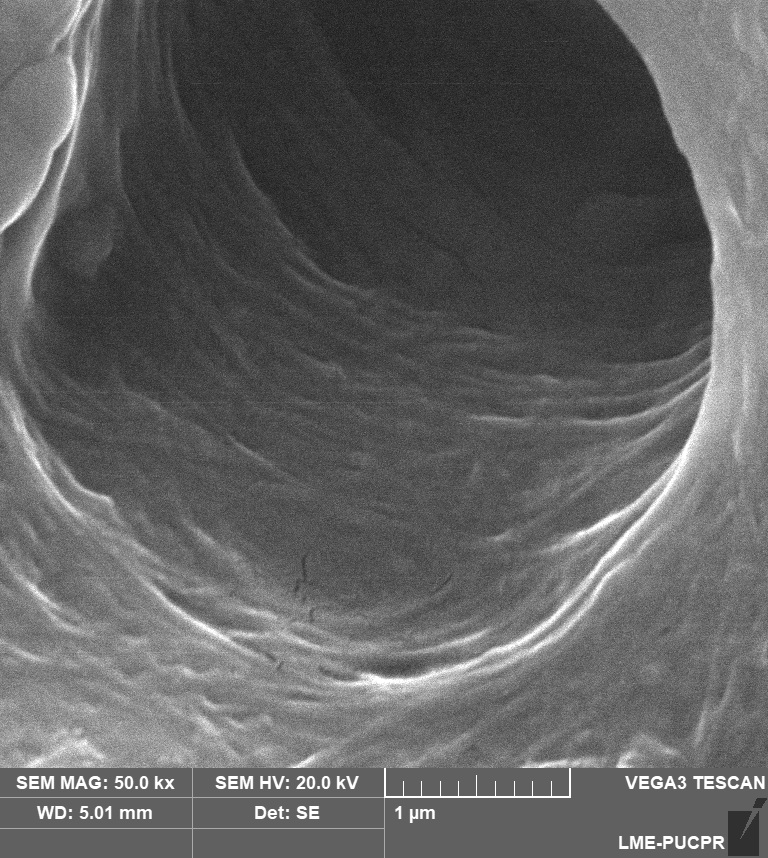

Supplement: S5 Data — (ZIP) [file pone.0337062.s005.zip › SEM/21Jan21/am2/am2_higido_50kx.tif]

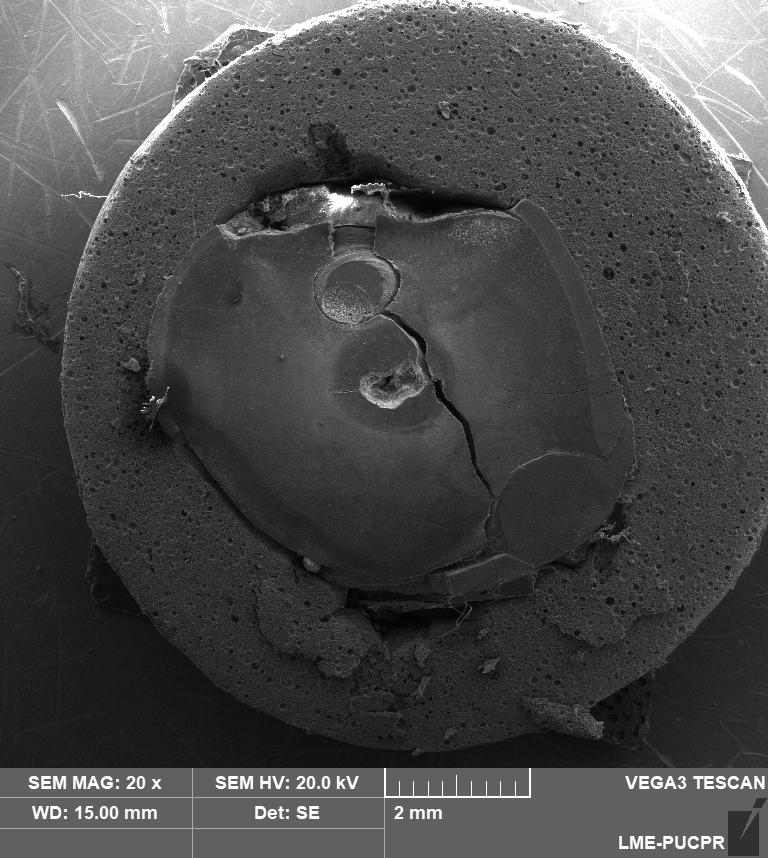

Supplement: S5 Data — (ZIP) [file pone.0337062.s005.zip › SEM/21Jan21/am1/am2_geral_20x.tif]

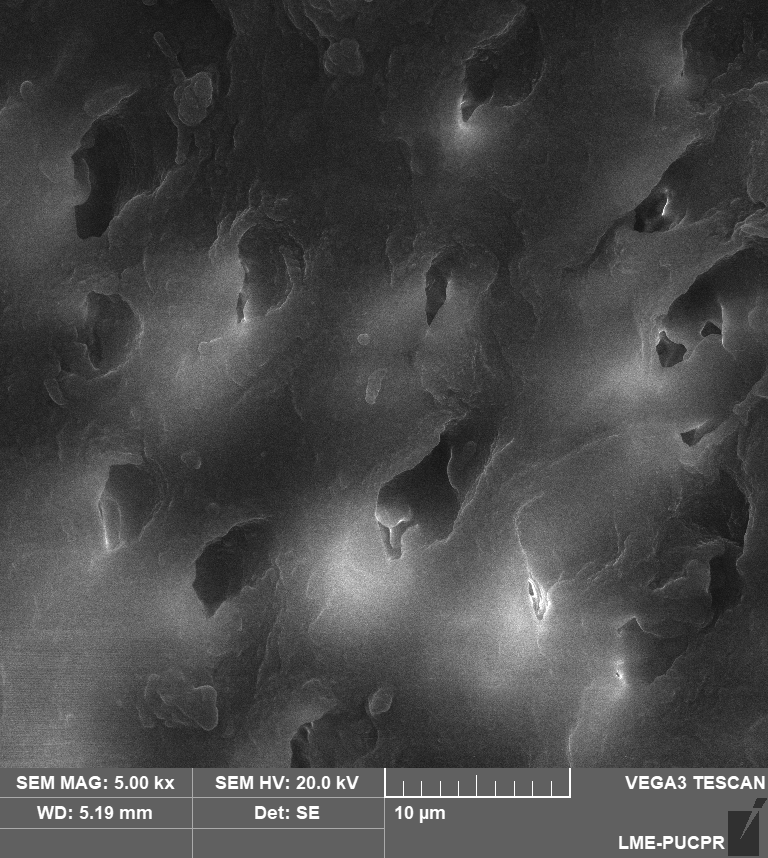

Supplement: S5 Data — (ZIP) [file pone.0337062.s005.zip › SEM/21Jan21/am1/am1_crr_5kx.tif]

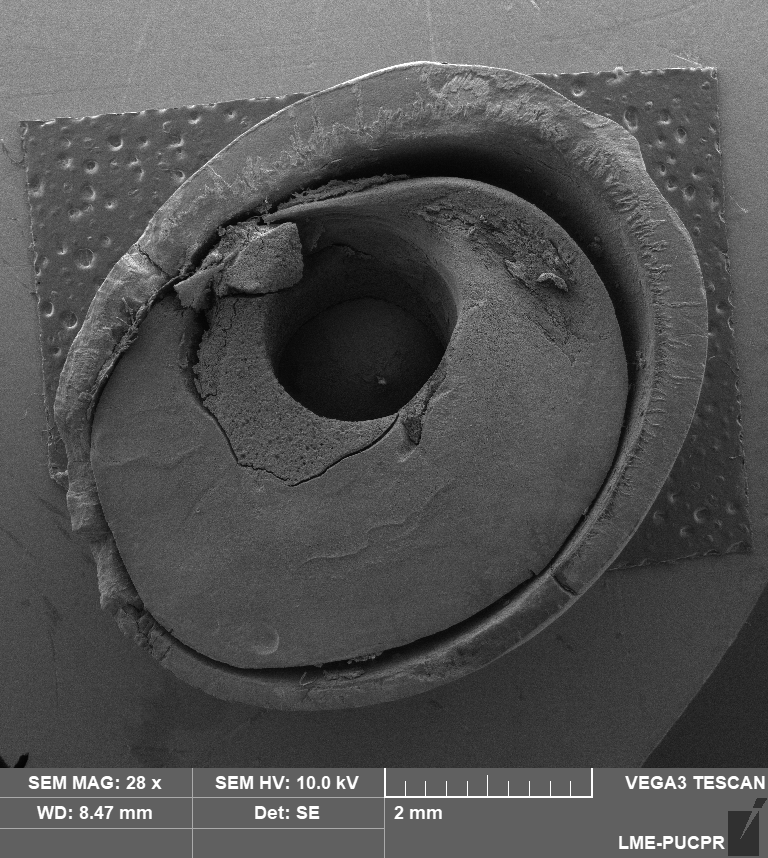

Supplement: S5 Data — (ZIP) [file pone.0337062.s005.zip › SEM/24Set21/irradiado_x28.tif]

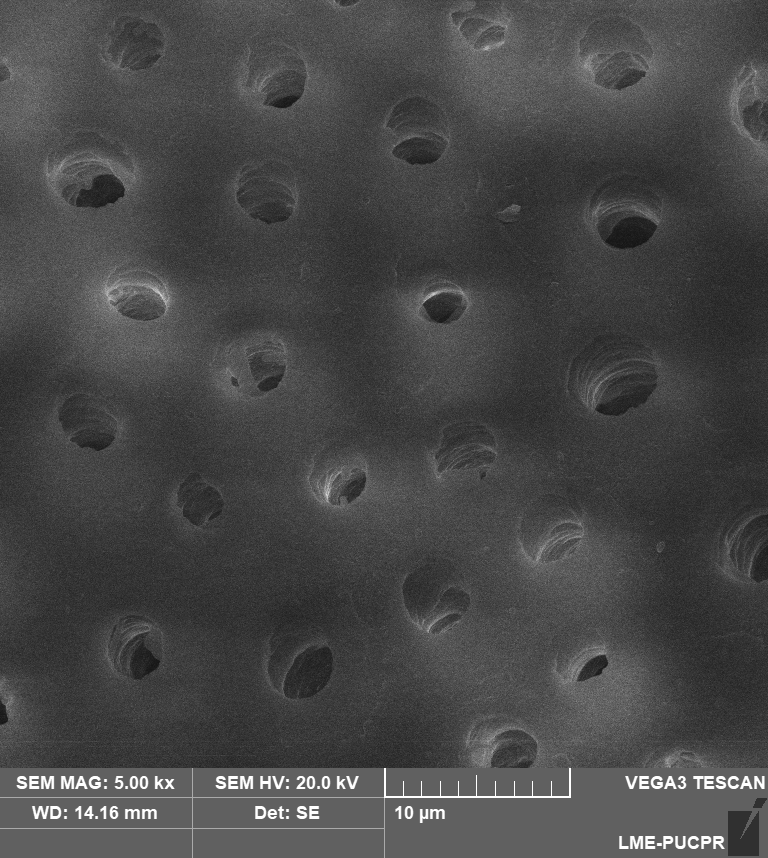

Supplement: S5 Data — (ZIP) [file pone.0337062.s005.zip › SEM/16Abr21/Am higido 2_x5k.tif]

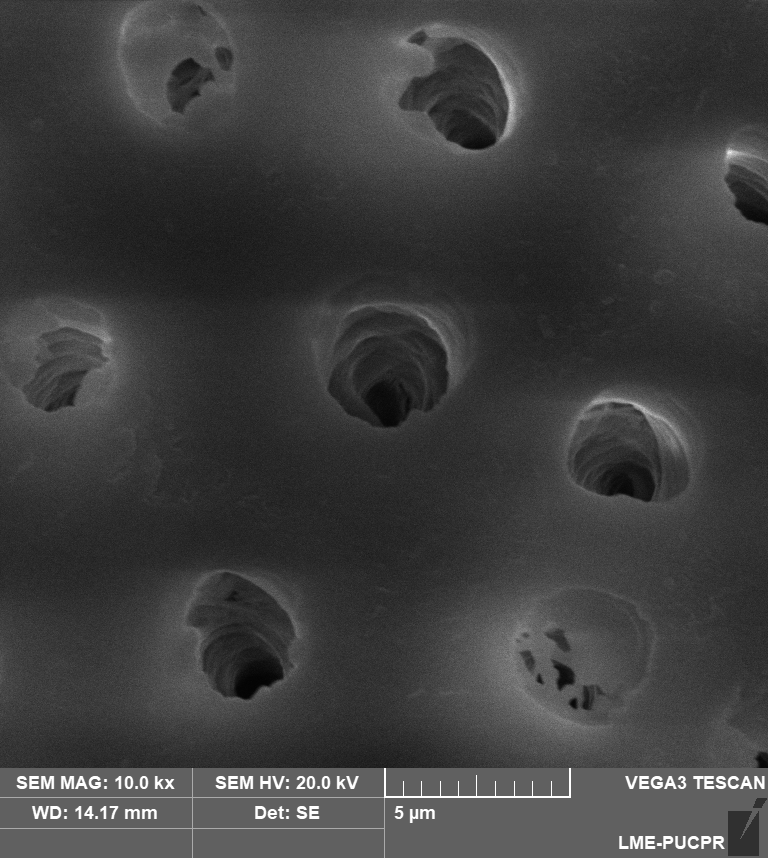

Supplement: S5 Data — (ZIP) [file pone.0337062.s005.zip › SEM/16Abr21/Am higido 2_x10ka.tif]

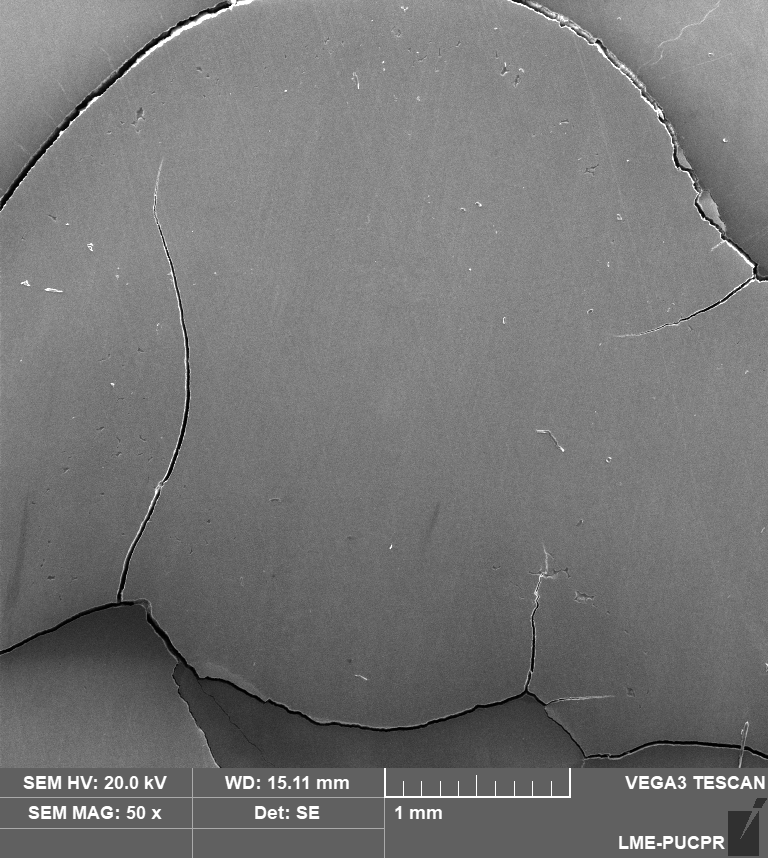

Supplement: S5 Data — (ZIP) [file pone.0337062.s005.zip › SEM/08Nov19/Dente_higido_50x.tif]

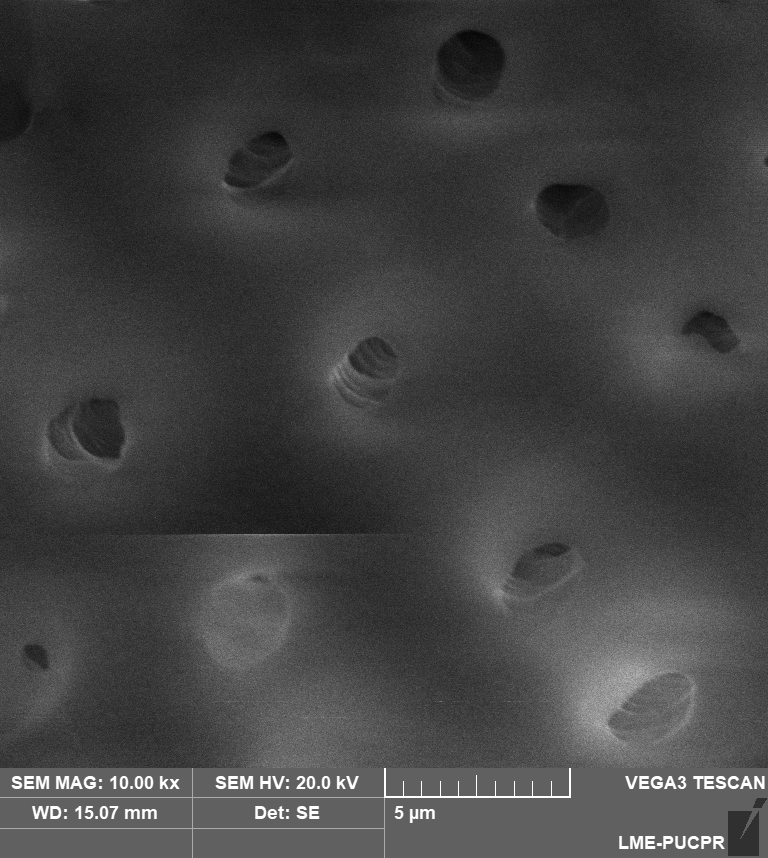

Supplement: S5 Data — (ZIP) [file pone.0337062.s005.zip › SEM/16Abr21/Am higido 1_x10ka.tif]

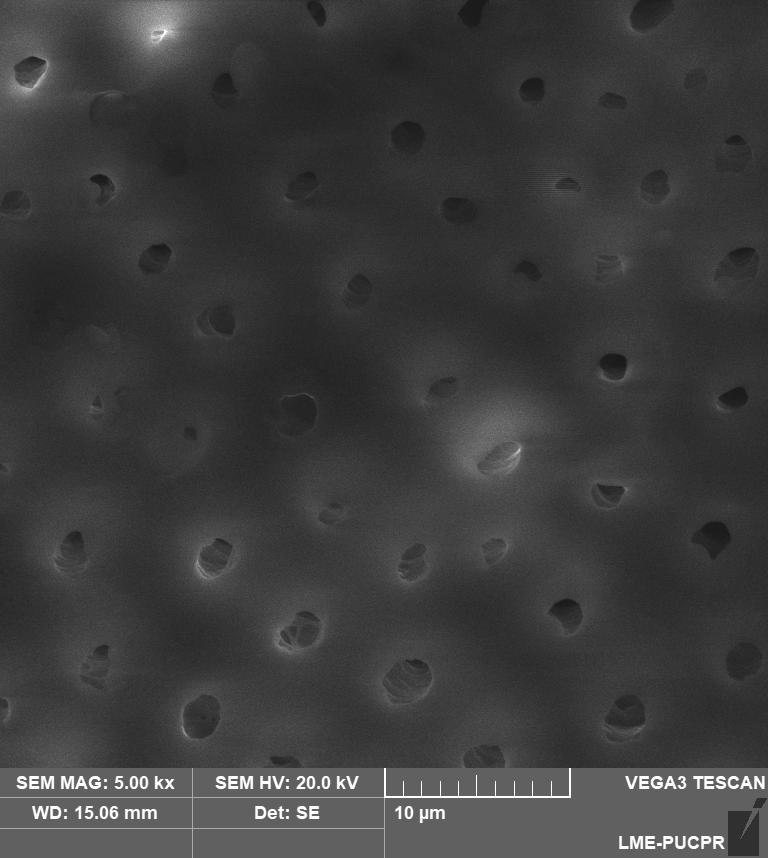

Supplement: S5 Data — (ZIP) [file pone.0337062.s005.zip › SEM/16Abr21/Am higido 1_x5ka.tif]

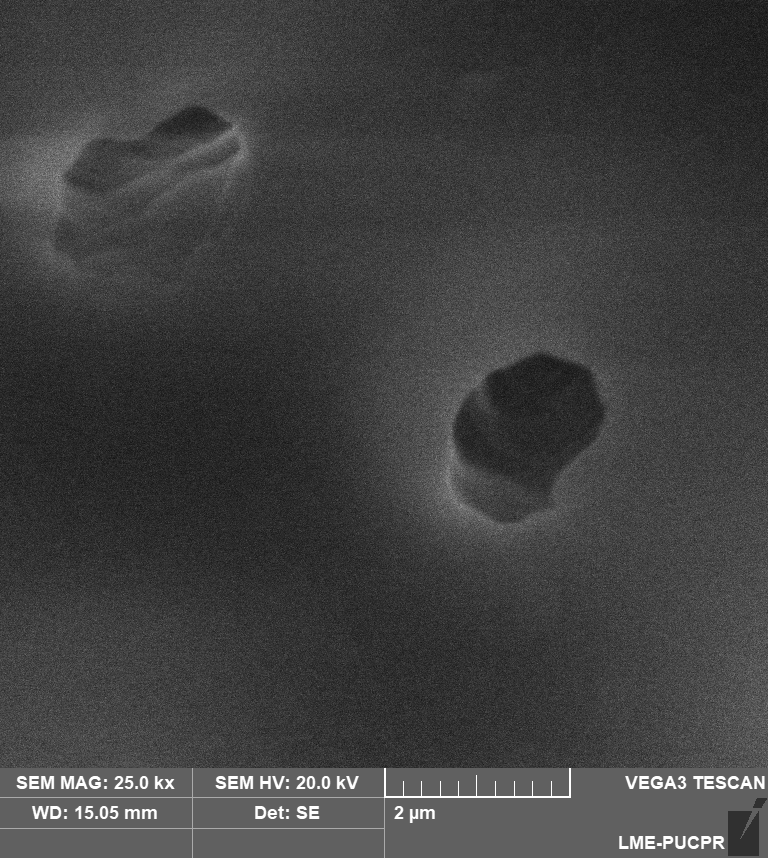

Supplement: S5 Data — (ZIP) [file pone.0337062.s005.zip › SEM/16Abr21/Am higido 1_x25k.tif]

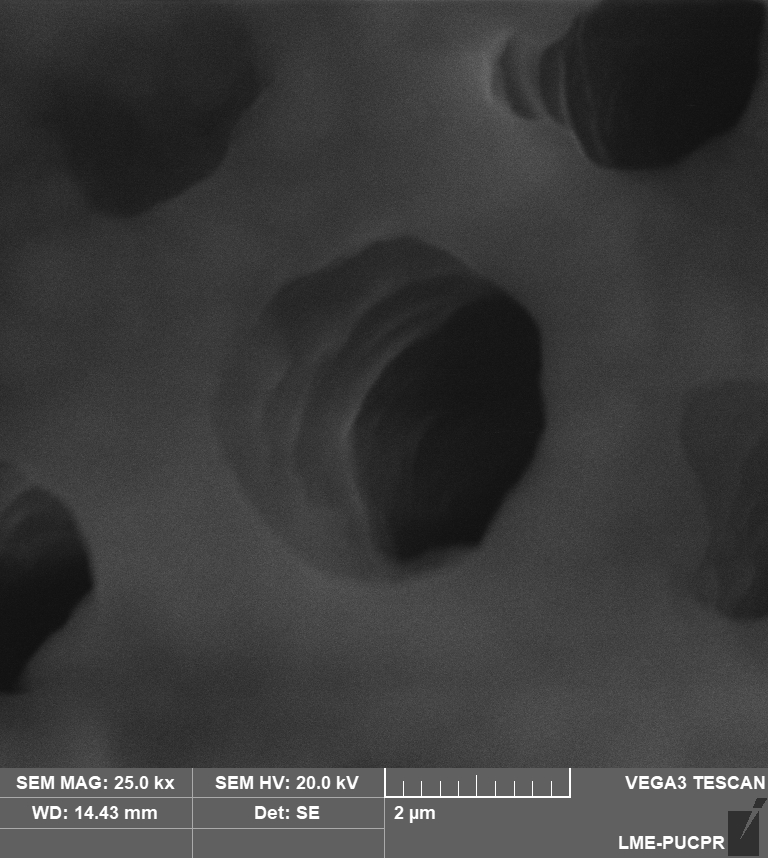

Supplement: S5 Data — (ZIP) [file pone.0337062.s005.zip › SEM/16Abr21/Am higido 3_x25ka.tif]

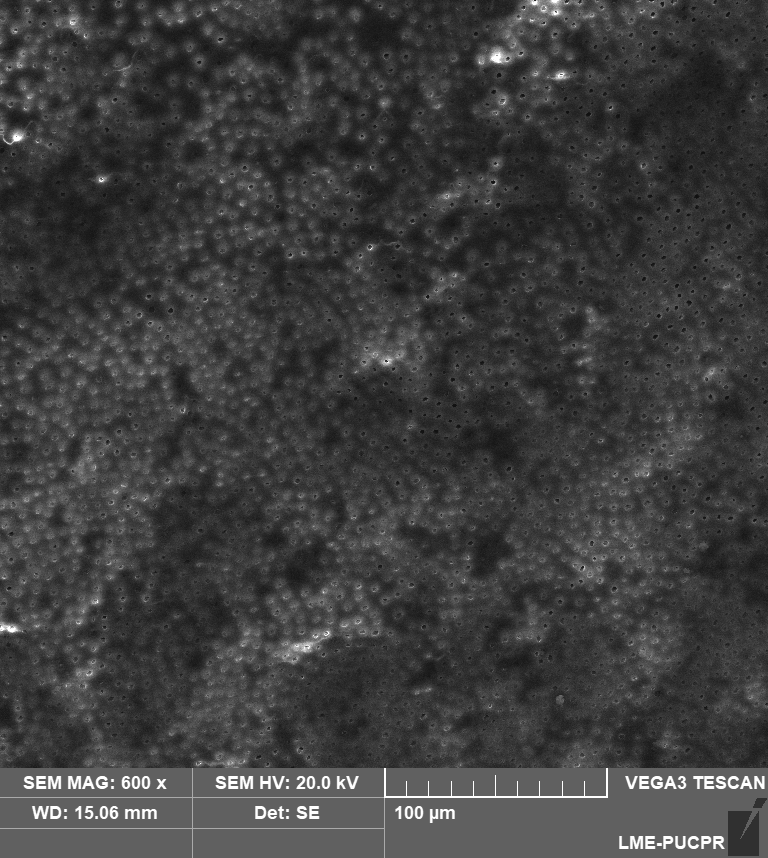

Supplement: S6 Data — (ZIP) [file pone.0337062.s006.zip › SEM/16Abr21/Am higido 1_x600.tif]

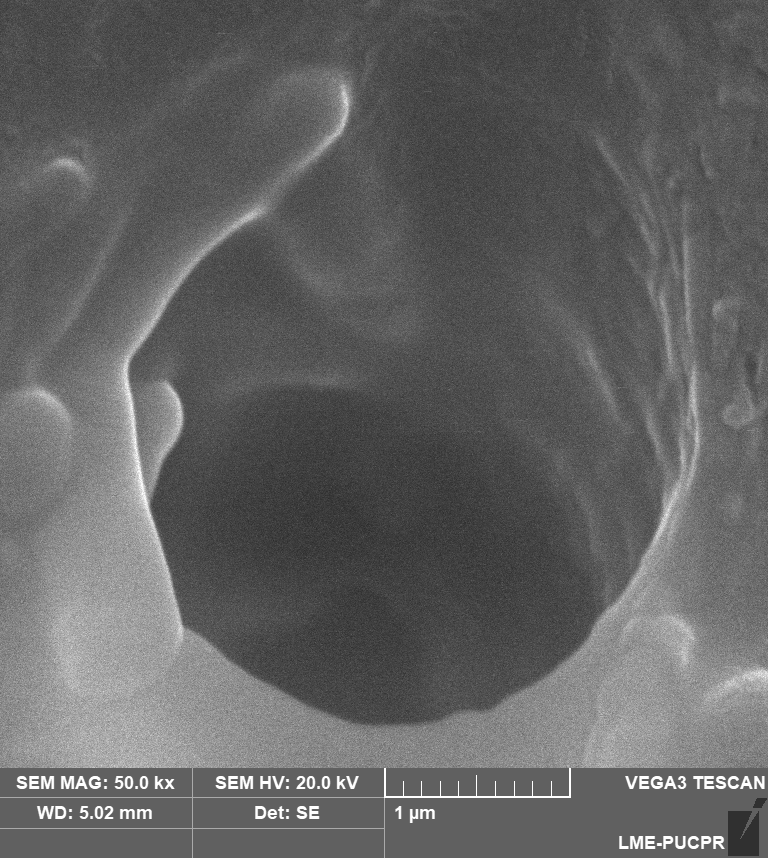

Supplement: S6 Data — (ZIP) [file pone.0337062.s006.zip › SEM/21Jan21/am1/am1_higido_50kx.tif]

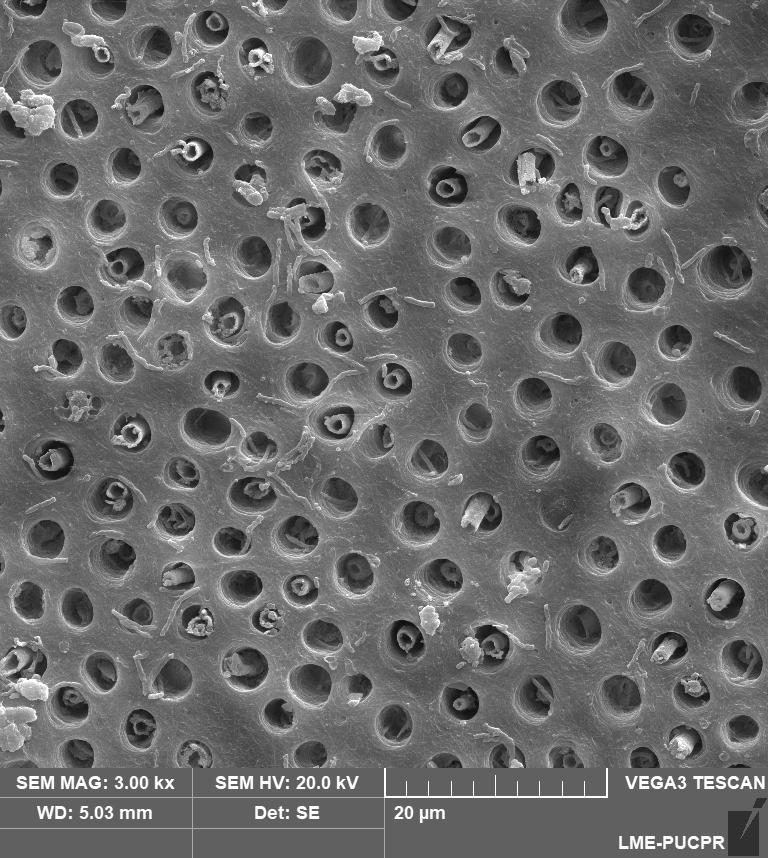

Supplement: S6 Data — (ZIP) [file pone.0337062.s006.zip › SEM/21Jan21/am2/am2_higido_3kx.tif]

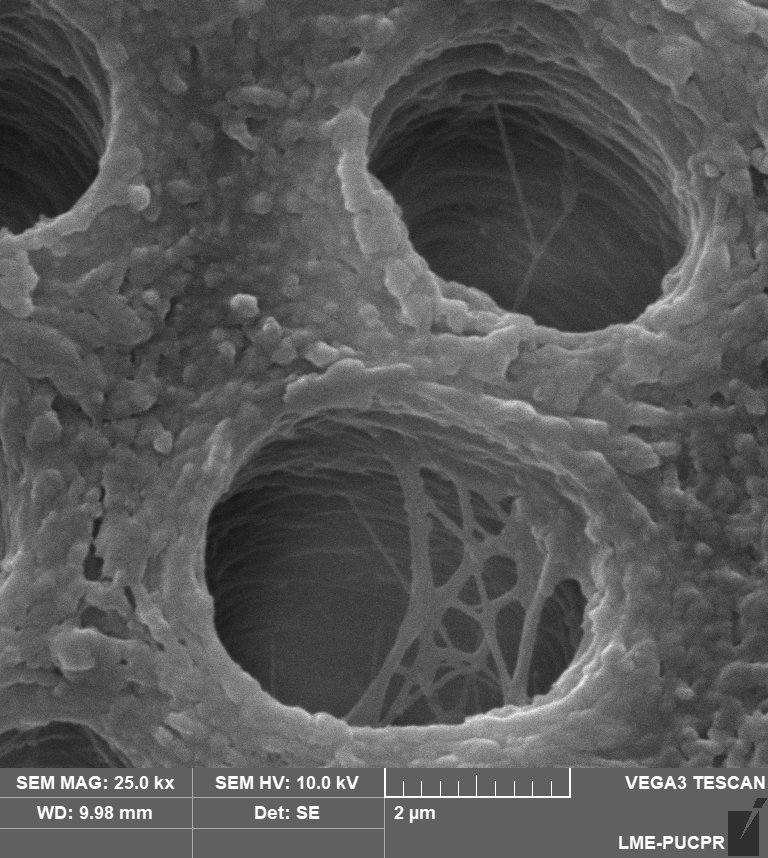

Supplement: S6 Data — (ZIP) [file pone.0337062.s006.zip › SEM/24Set21/higido_x25k.tif]

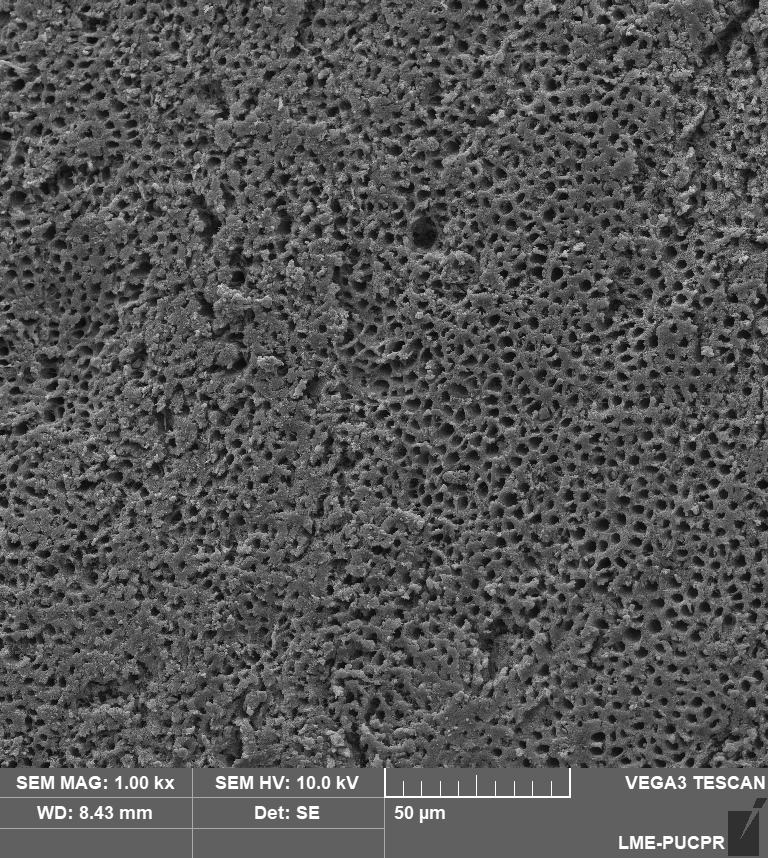

Supplement: S6 Data — (ZIP) [file pone.0337062.s006.zip › SEM/24Set21/irradiado_x1k.tif]

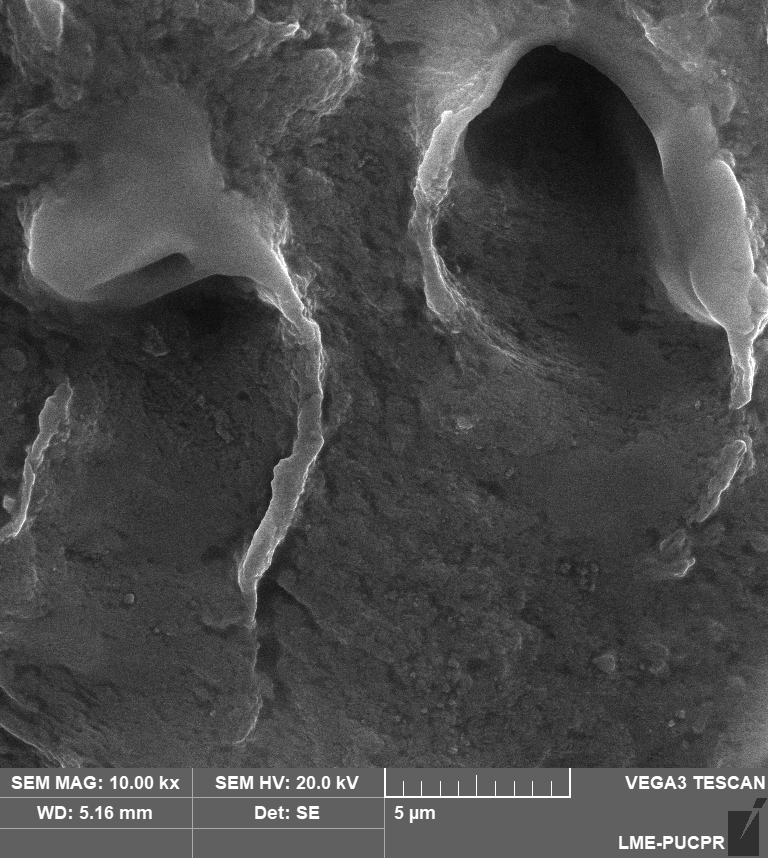

Supplement: S6 Data — (ZIP) [file pone.0337062.s006.zip › SEM/21Jan21/am2/am2_crr_10kx.tif]

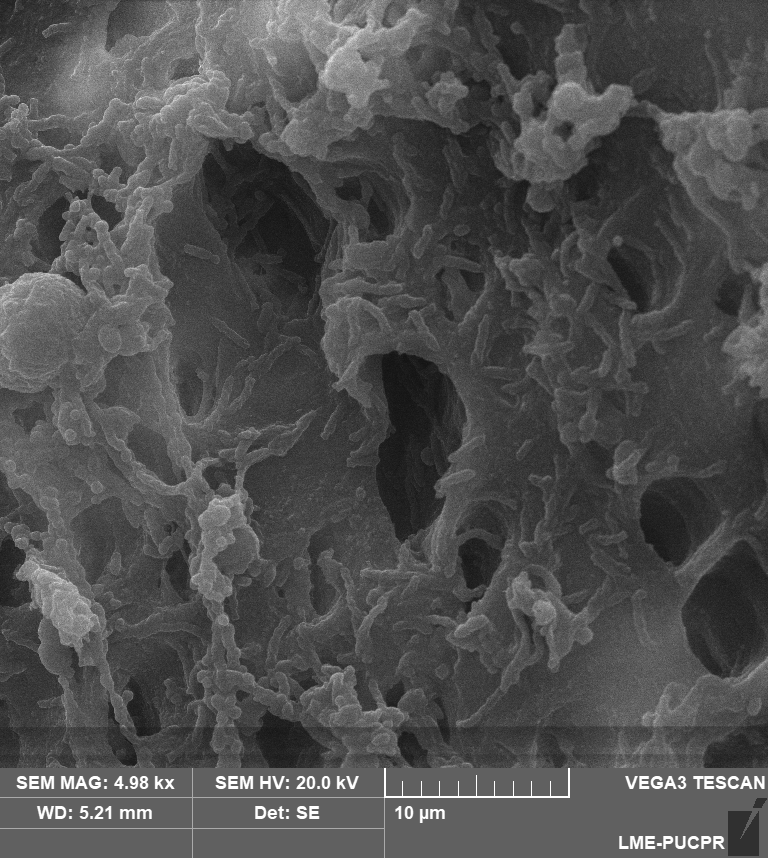

Supplement: S6 Data — (ZIP) [file pone.0337062.s006.zip › SEM/21Jan21/am1/am1_crr_5xka.tif]

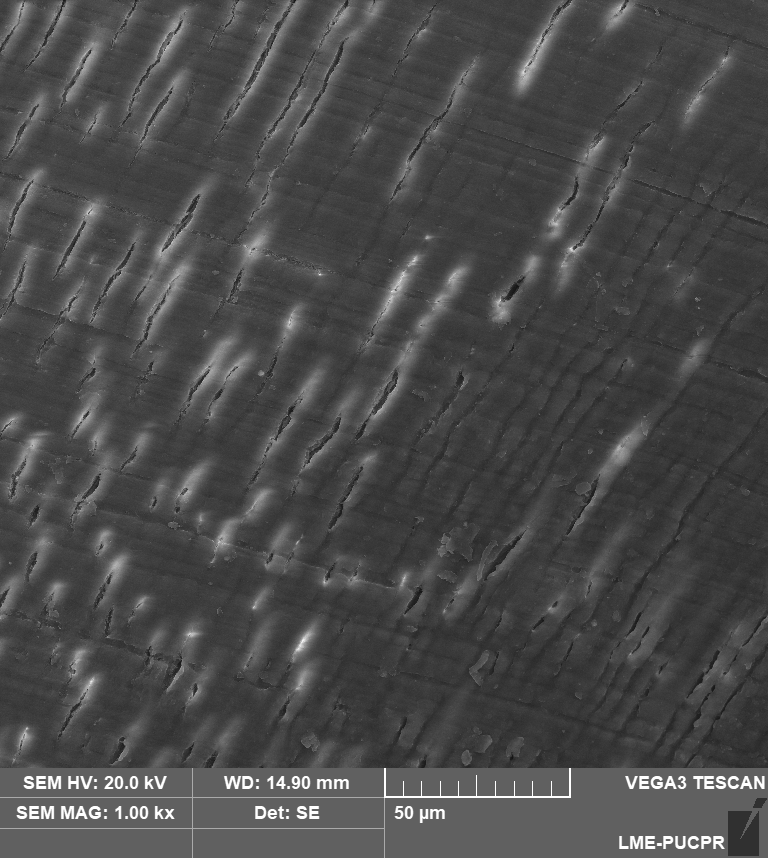

Supplement: S6 Data — (ZIP) [file pone.0337062.s006.zip › SEM/08Nov19/CRR_10Kx_B.tif]

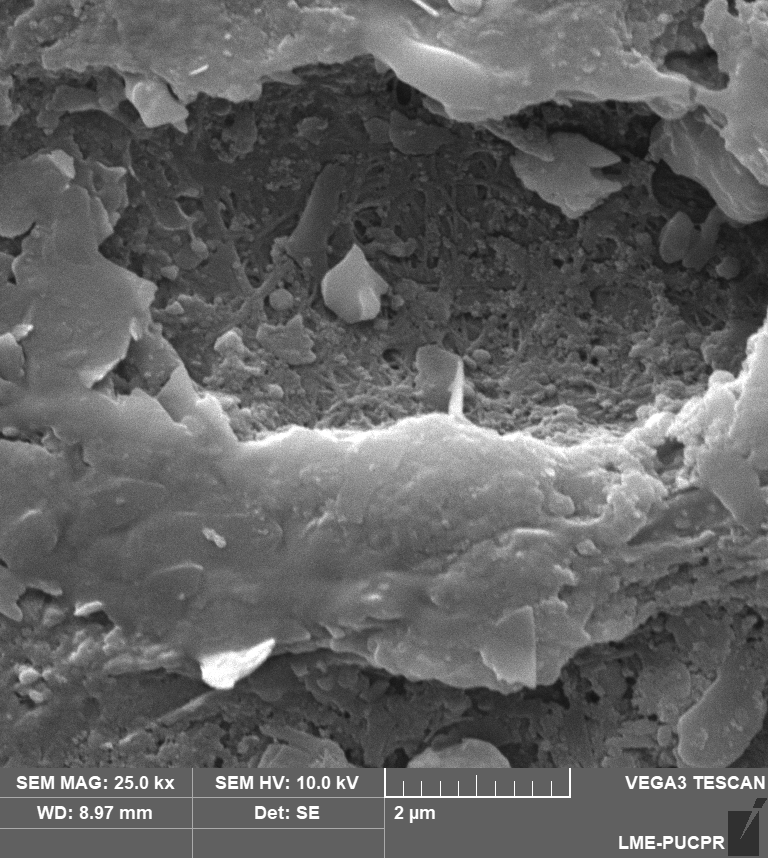

Supplement: S6 Data — (ZIP) [file pone.0337062.s006.zip › SEM/24Set21/crr_x25k.tif]

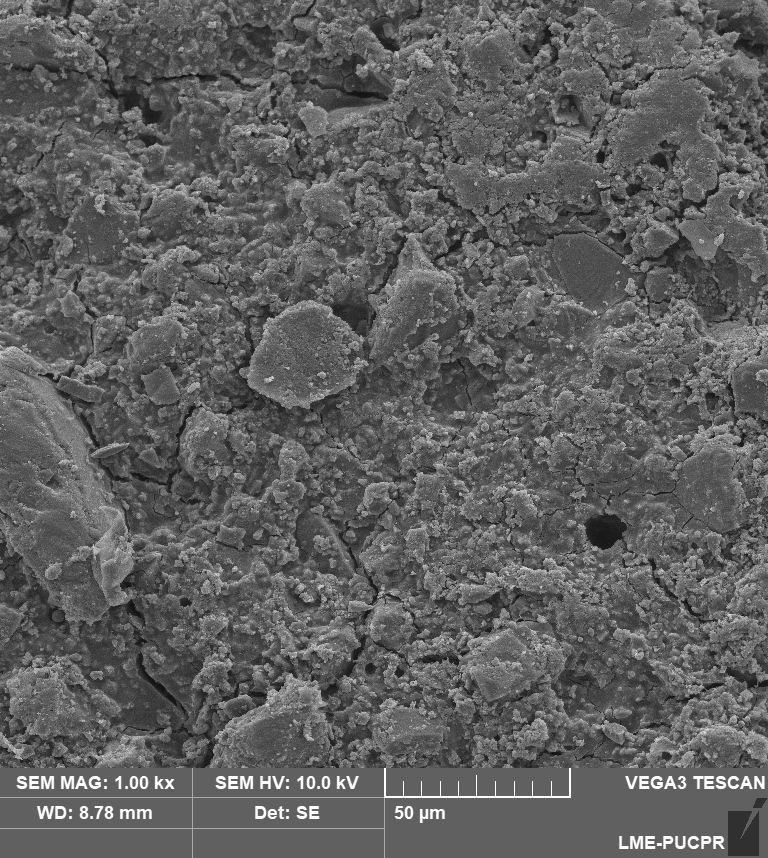

Supplement: S6 Data — (ZIP) [file pone.0337062.s006.zip › SEM/24Set21/irradiado3_x1k.tif]

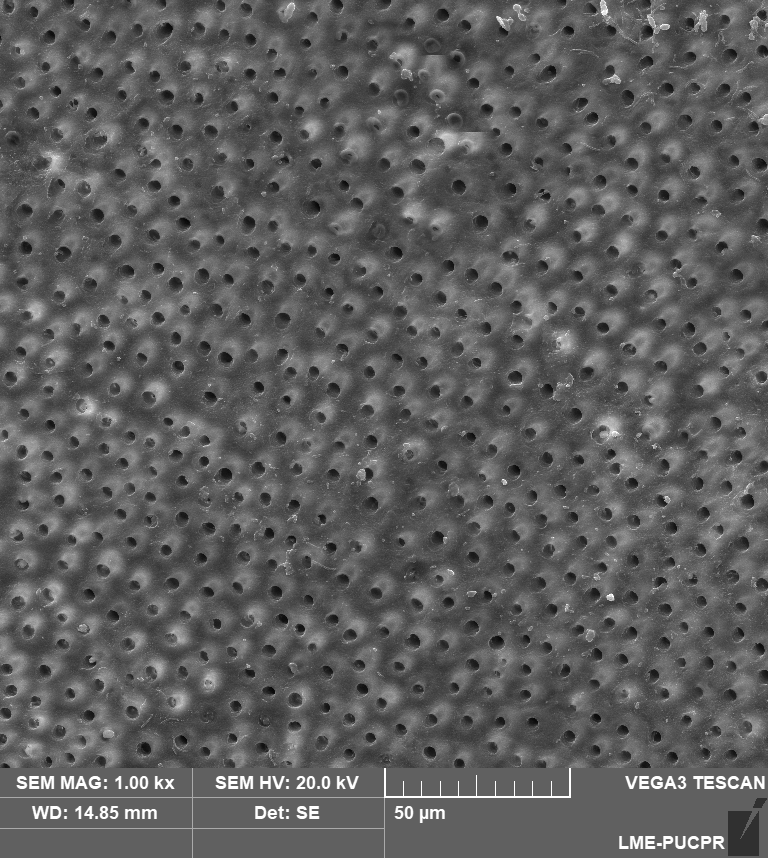

Supplement: S6 Data — (ZIP) [file pone.0337062.s006.zip › SEM/21Jan21/am2/am2_higido_1kx.tif]

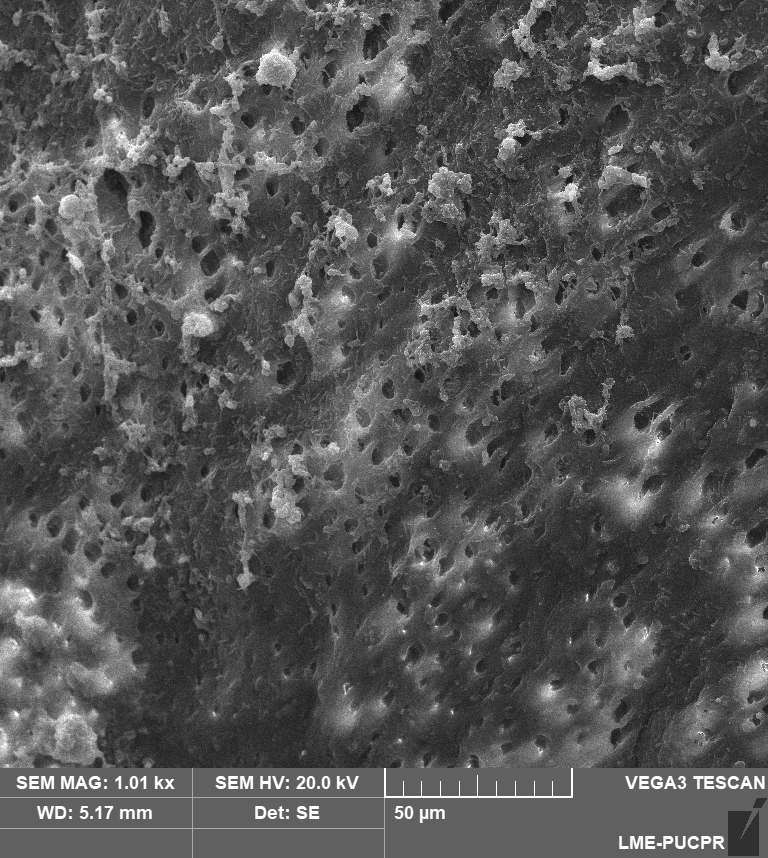

Supplement: S6 Data — (ZIP) [file pone.0337062.s006.zip › SEM/21Jan21/am1/am1_crr_1xka.tif]

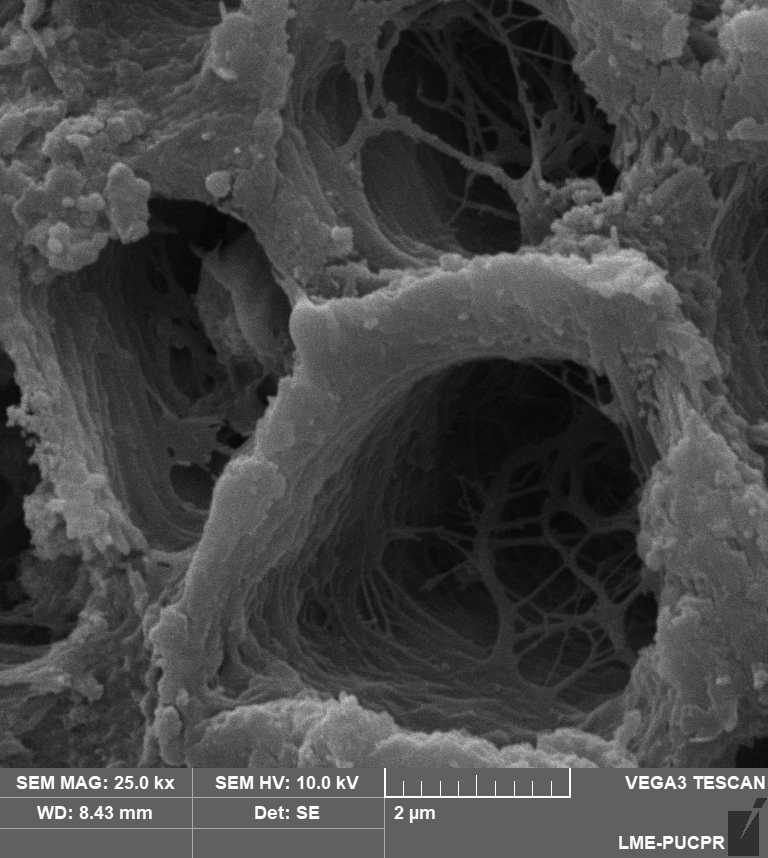

Supplement: S6 Data — (ZIP) [file pone.0337062.s006.zip › SEM/24Set21/irradiado_x25k.tif]

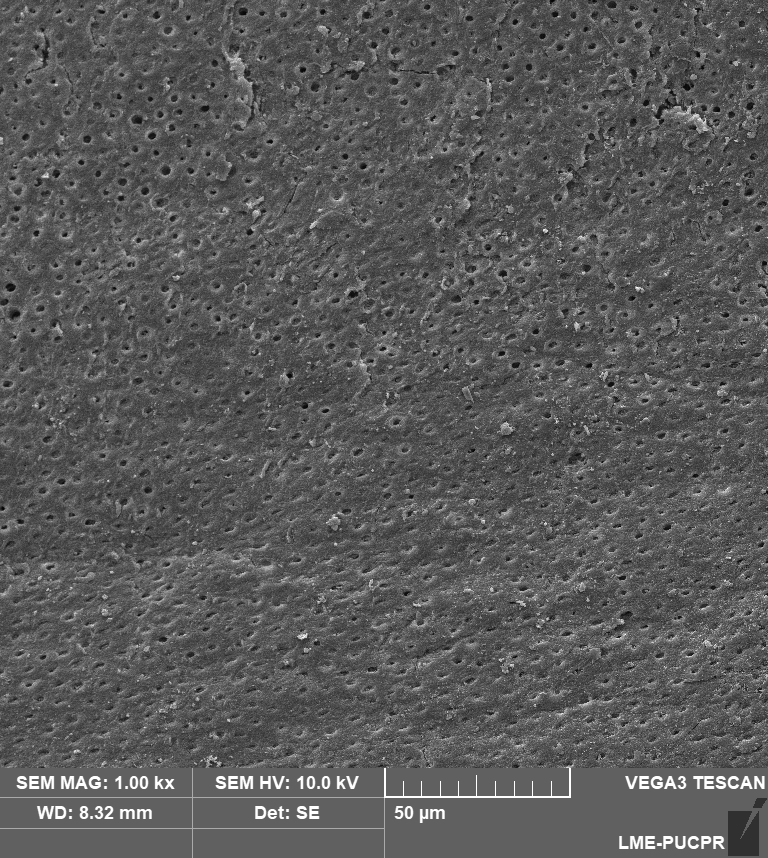

Supplement: S6 Data — (ZIP) [file pone.0337062.s006.zip › SEM/24Set21/irradiado2_x1k.tif]

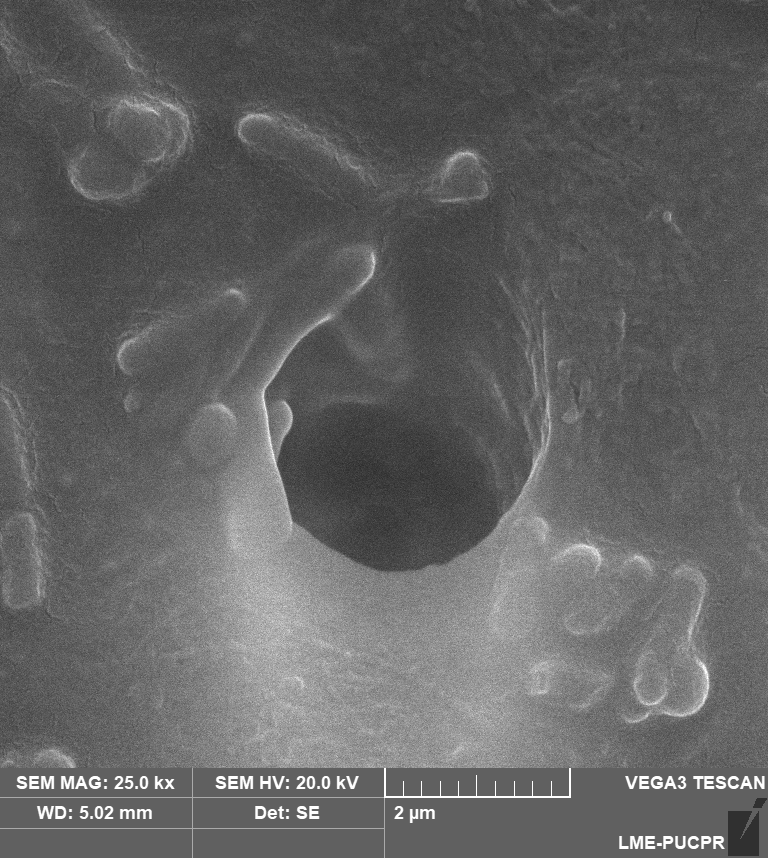

Supplement: S6 Data — (ZIP) [file pone.0337062.s006.zip › SEM/21Jan21/am1/am1_higido_25kx.tif]

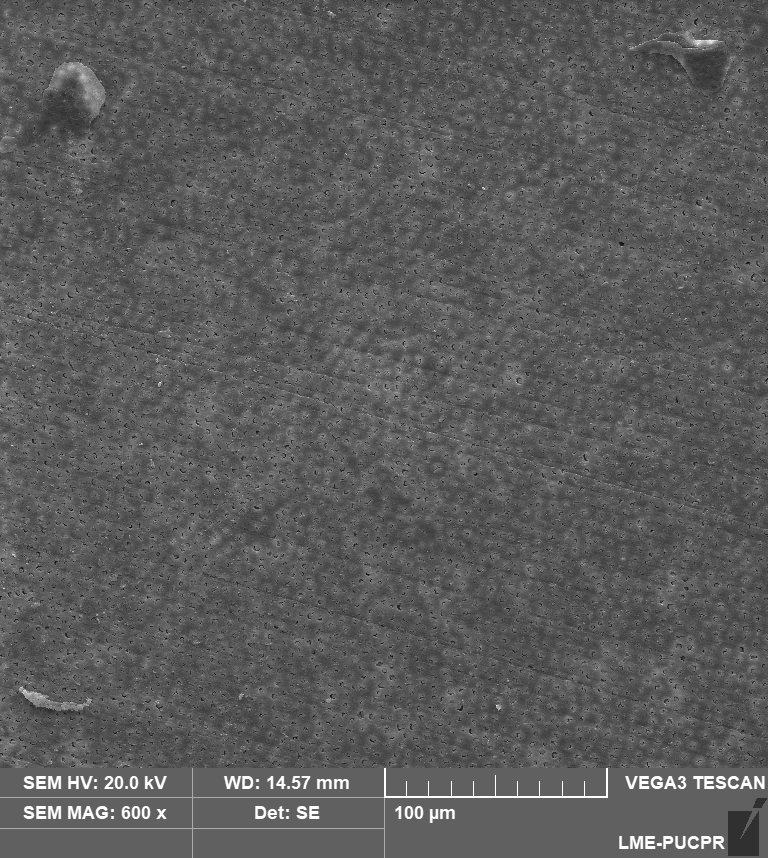

Supplement: S6 Data — (ZIP) [file pone.0337062.s006.zip › SEM/08Nov19/Dente_higido2_600x.tif]

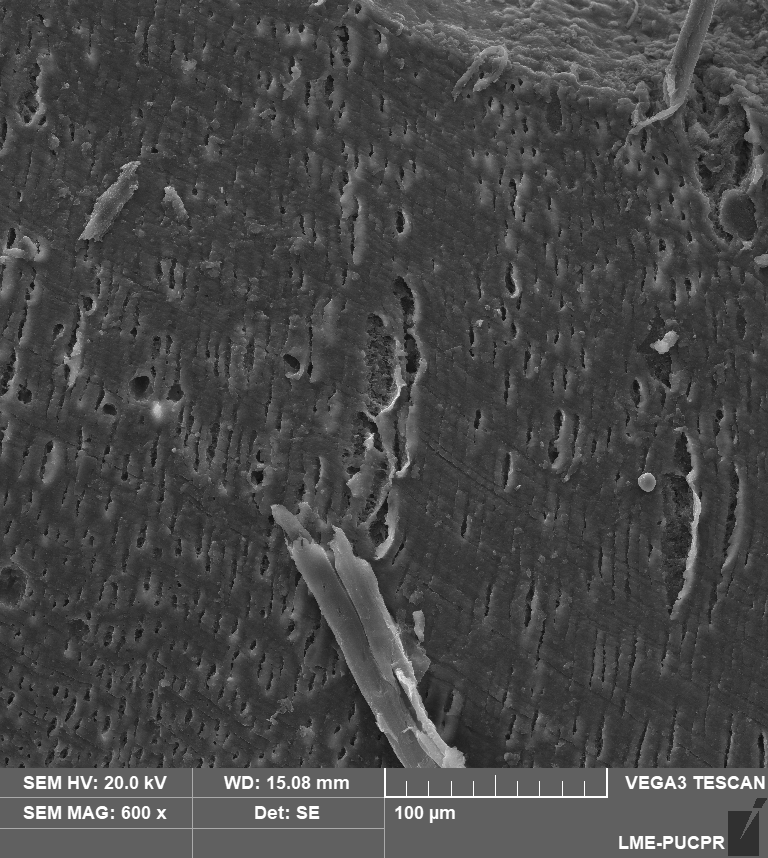

Supplement: S6 Data — (ZIP) [file pone.0337062.s006.zip › SEM/08Nov19/CRR_600x.tif]

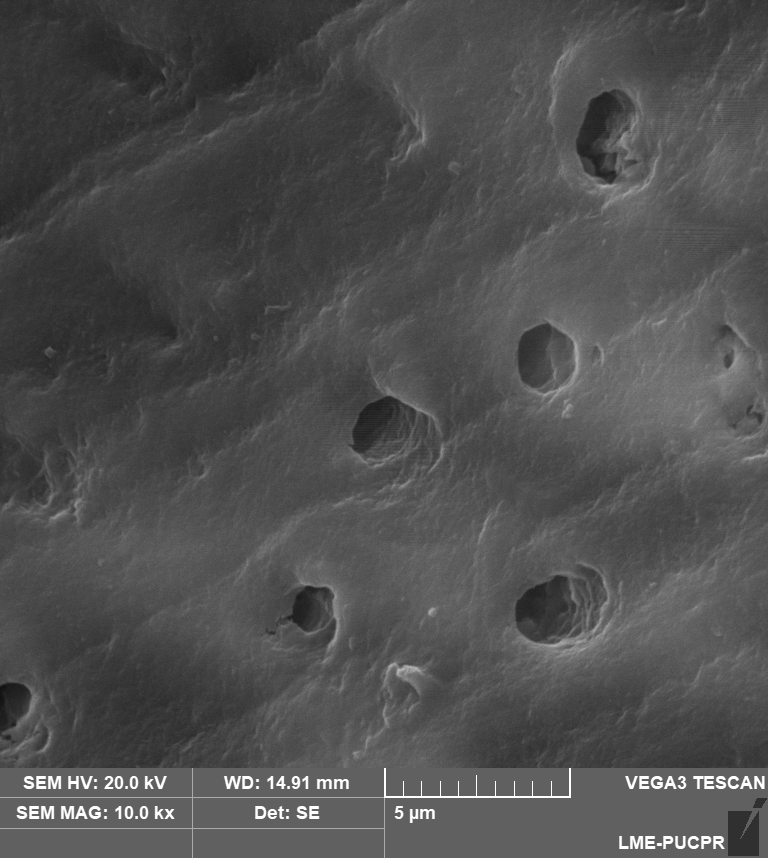

Supplement: S6 Data — (ZIP) [file pone.0337062.s006.zip › SEM/08Nov19/CRR2_10Kx_B.tif]

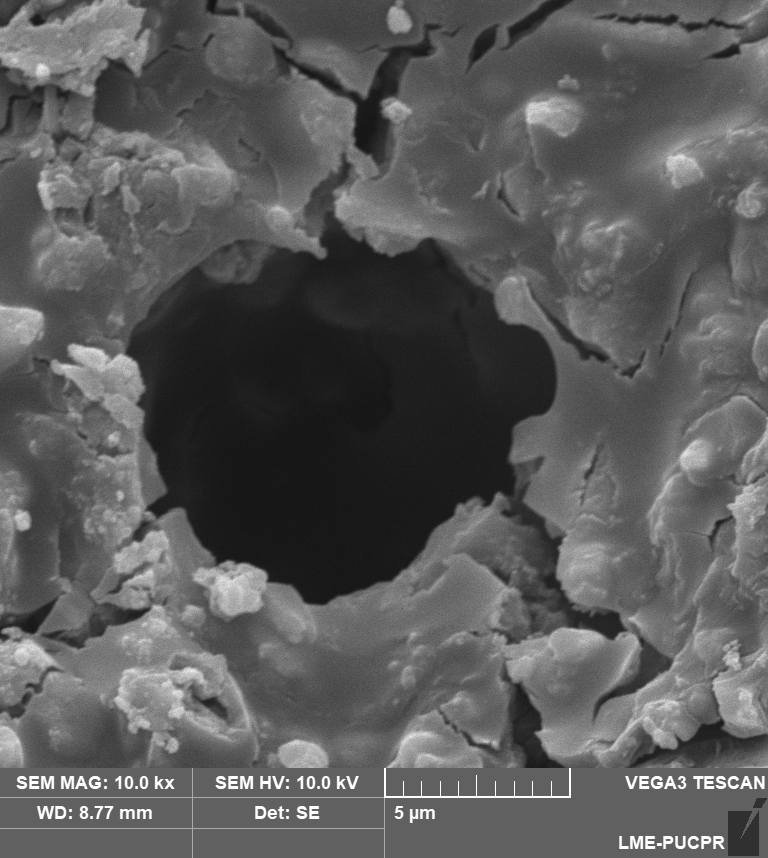

Supplement: S6 Data — (ZIP) [file pone.0337062.s006.zip › SEM/24Set21/irradiado3_x10k.tif]

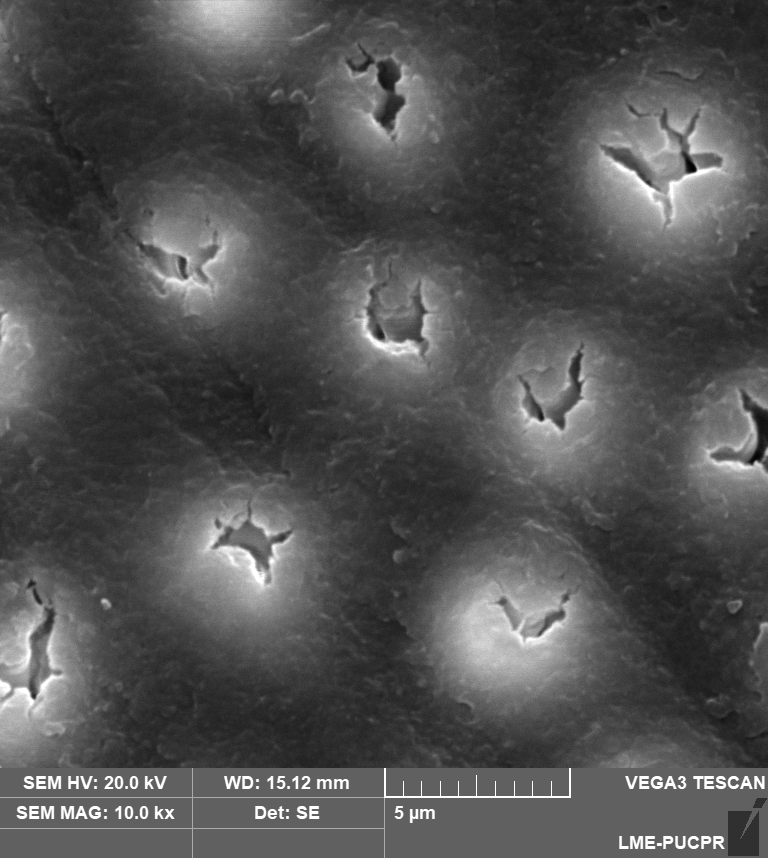

Supplement: S6 Data — (ZIP) [file pone.0337062.s006.zip › SEM/08Nov19/Dente_higido_10Kx.tif]

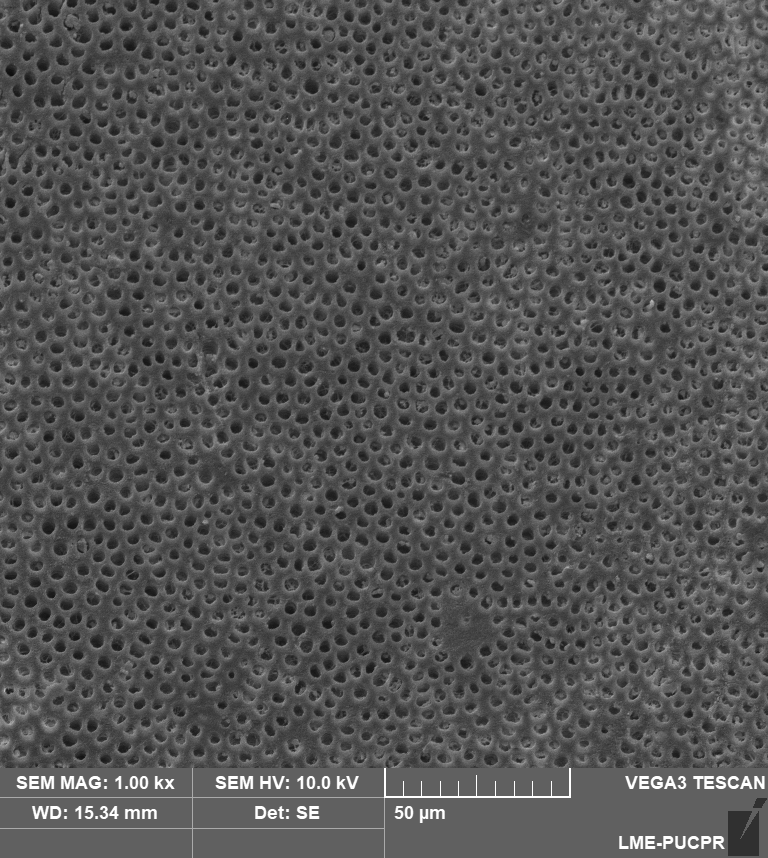

Supplement: S6 Data — (ZIP) [file pone.0337062.s006.zip › SEM/24Set21/higido_x1k.tif]

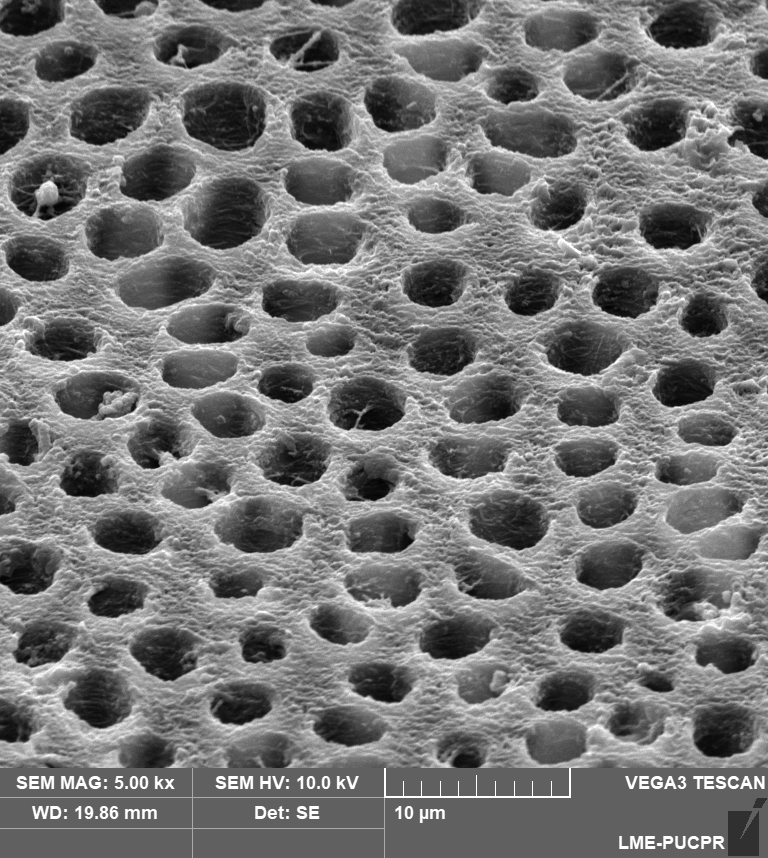

Supplement: S6 Data — (ZIP) [file pone.0337062.s006.zip › SEM/24Set21/higido_x5k tilt 55.tif]

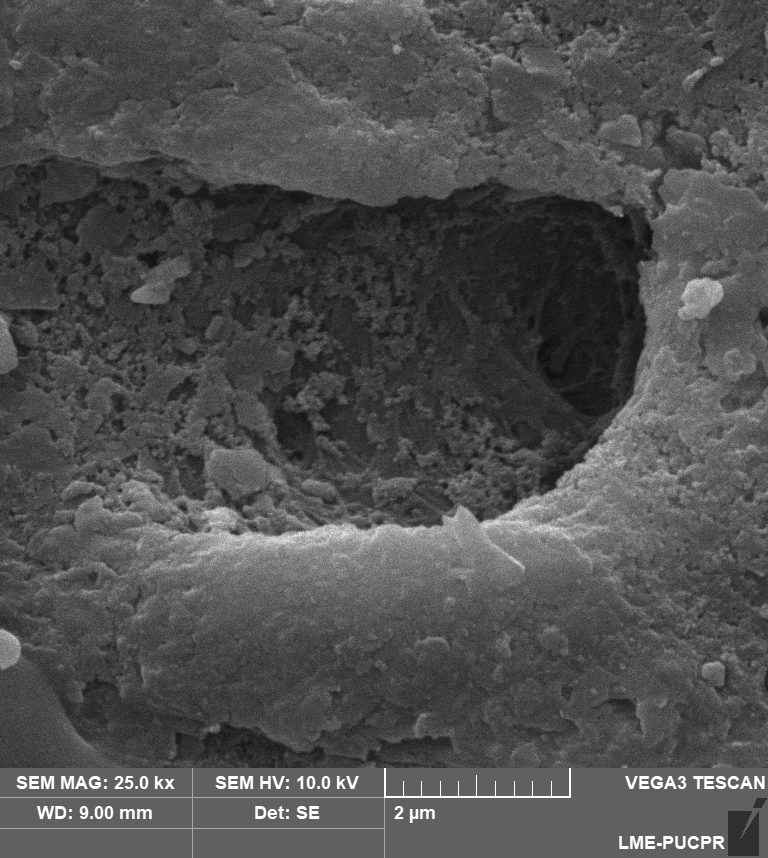

Supplement: S6 Data — (ZIP) [file pone.0337062.s006.zip › SEM/24Set21/crr_x25kb.tif]

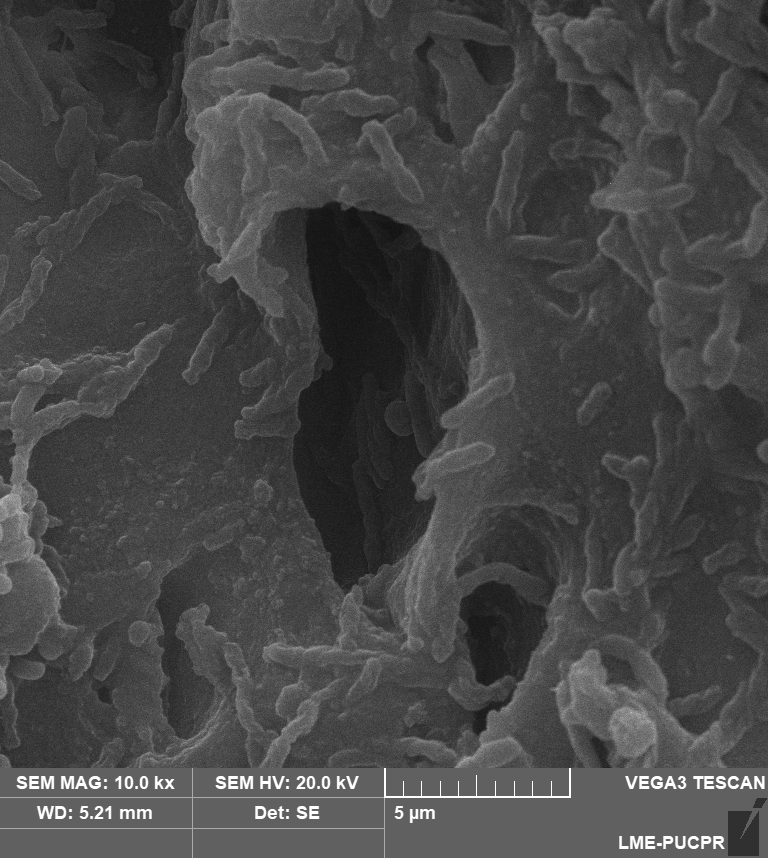

Supplement: S6 Data — (ZIP) [file pone.0337062.s006.zip › SEM/21Jan21/am1/am1_crr_10xka.tif]

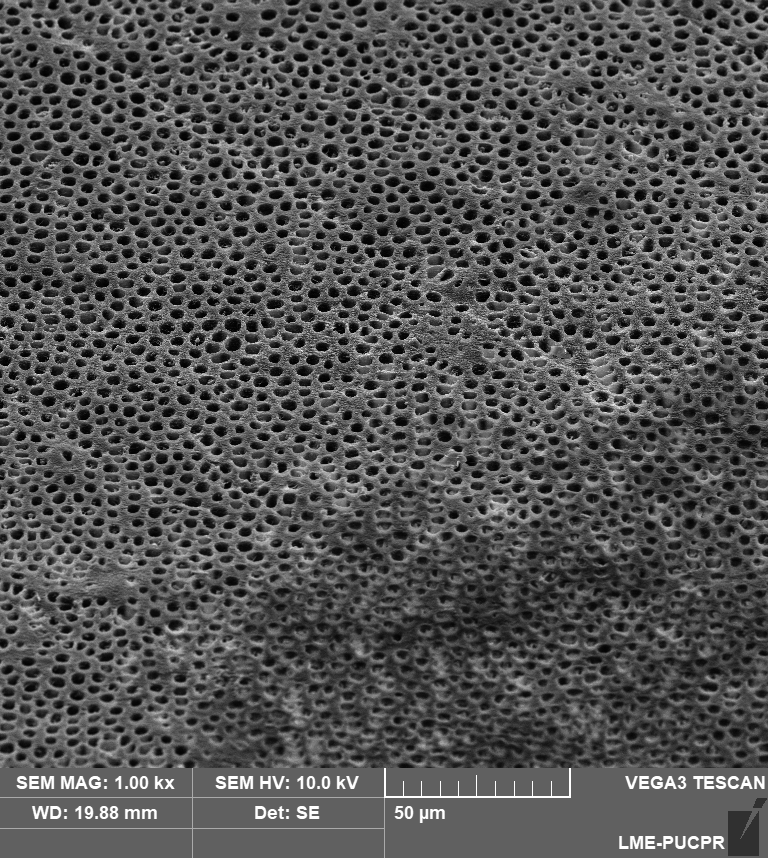

Supplement: S6 Data — (ZIP) [file pone.0337062.s006.zip › SEM/24Set21/higido_x1k tilt 55.tif]

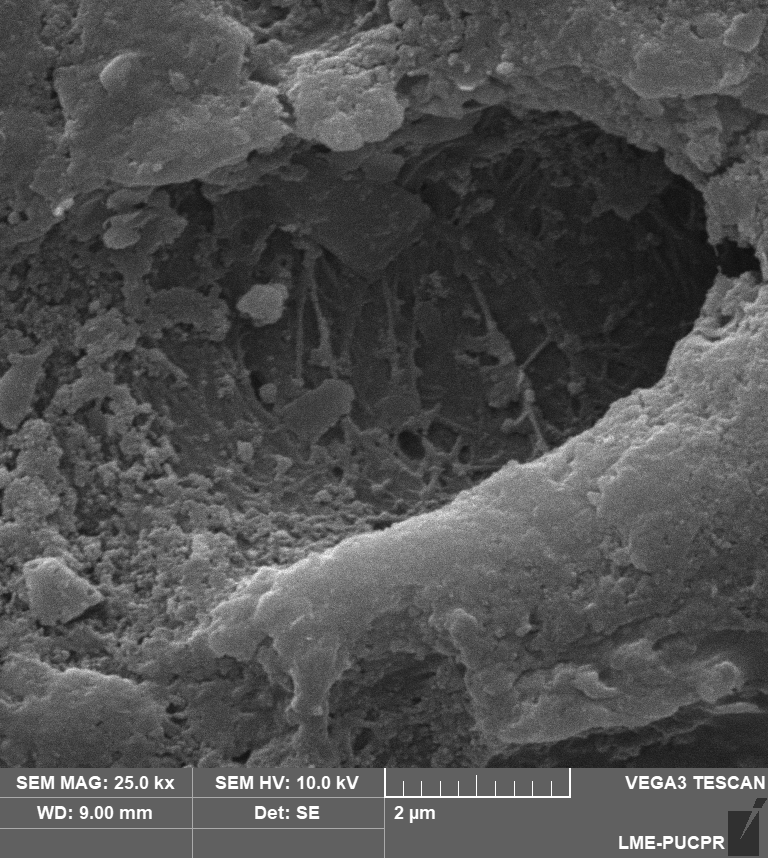

Supplement: S6 Data — (ZIP) [file pone.0337062.s006.zip › SEM/24Set21/crr_x25kc.tif]

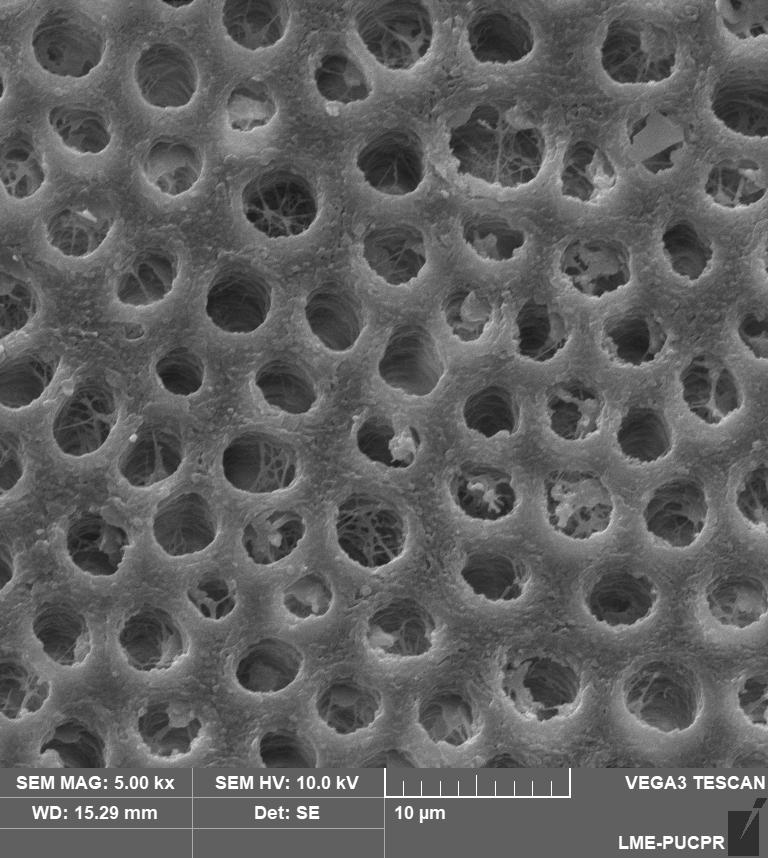

Supplement: S6 Data — (ZIP) [file pone.0337062.s006.zip › SEM/24Set21/higido_x5k.tif]

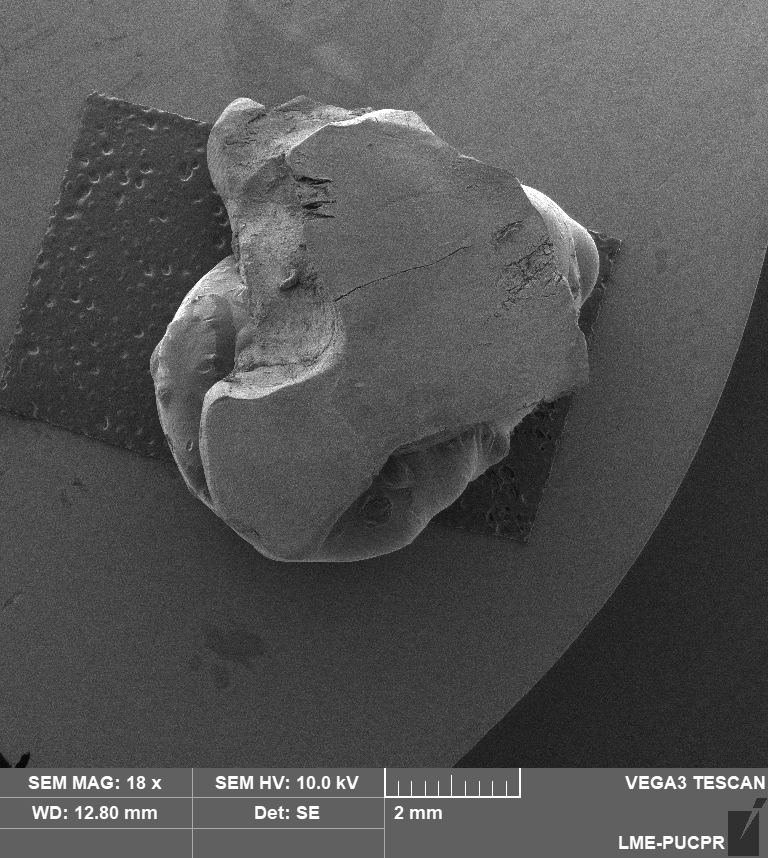

Supplement: S6 Data — (ZIP) [file pone.0337062.s006.zip › SEM/24Set21/crr_x18.tif]

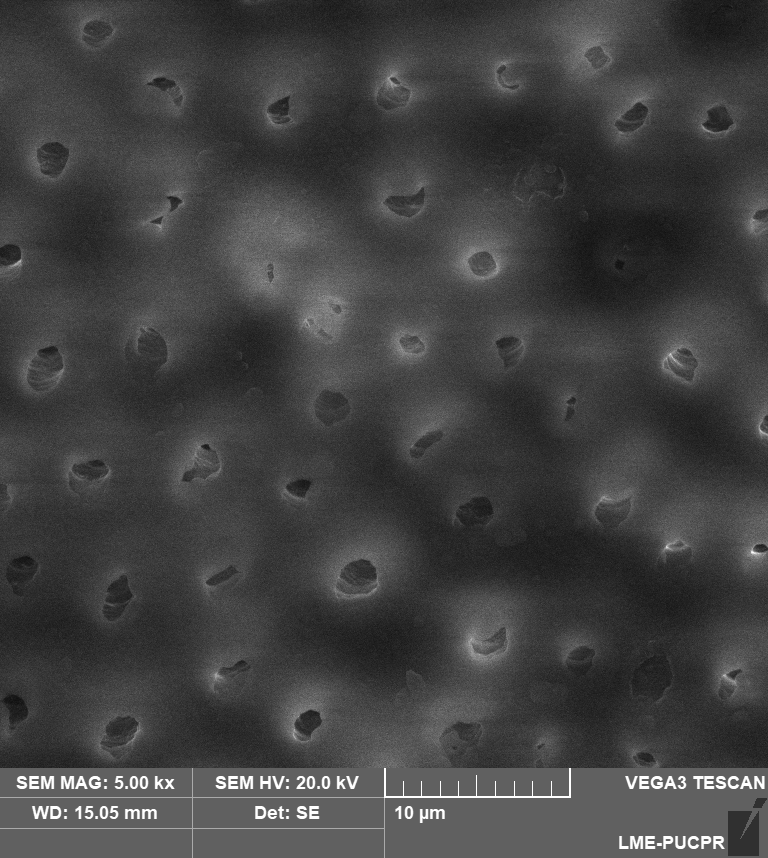

Supplement: S6 Data — (ZIP) [file pone.0337062.s006.zip › SEM/16Abr21/Am higido 1_x5k.tif]
