# Supplementary material for: Microstructural and chemical characterization of radiation-induced carious dentin of teeth submitted to ionizing radiation as a head and neck cancer therapy
Source: PLoS One. 2025 Dec 12;20(12):e0337062. doi: 10.1371/journal.pone.0337062 (PMC12700452; doi:10.1371/journal.pone.0337062)
Supplement: S3 Data — (ZIP) [file pone.0337062.s003.zip › BrunaOdo/crr_mesmo/Theta = 10.0000 ()_Report.htm]

Match! message


## 

# Match! Phase Analysis Report

## Paulo Soares

## Sample: Theta = 10.0000 ()

|  |
| --- |
| ***Sample Data*** |
| File name | crr\_mesmo.RAW |
| File path | C:/xddat/BrunaOdo/crr\_mesmo |
| Data collected | Sep 17, 2021 15:29:10 |
| Data range | 15.000º - 55.000º |
 Number of points | 2001 || Step size | 0.020 |
| Rietveld refinement converged | No |
| Alpha2 subtracted | No |
| Background subtr. | Yes |
| Data smoothed | Yes |
| Radiation | X-rays |
| Wavelength | 1.540600 Å |

## Matched Phases

|  |  |  |  |
| --- | --- | --- | --- |
| ***Index*** | ***Amount (%)*** | ***Name*** | ***Formula sum*** |
| A |  | Calcium Hydrogen Phosphate | Ca H2 P2 O7 |
| B |  | Calcium Phosphate Hydroxide Apatite-(CaOH), syn | ( Ca )10 ( P O4 )6 ( O H )2 |
|  | *6.1* | *Unidentified peak area* |  |

|  |
| --- |
| ***A: Calcium Hydrogen Phosphate*** |
| Formula sum | Ca H2 P2 O7 |
||  |  |
| --- | --- |
| Entry number | 00-051-0200 |
| Total number of peaks | 115 |
 Space group | C2/c |
 Crystal system | monoclinic || Unit cell | a= 7.3294 Å b= 8.1300 Å c= 9.7665 Å β= 101.239 º |
| I/Ic | 2.15 |
| Calc. density | 2.514 g/cm³ |
| Reference | Schneider, M., Trommer, J., Wilde, L., Inst. f. Angewandte Chemie, Berlin, Germany., "", ICDD Grant-in-Aid , (1999) |

  

|  |
| --- |
| ***B: Calcium Phosphate Hydroxide Apatite-(CaOH), syn*** |
| Formula sum | ( Ca )10 ( P O4 )6 ( O H )2 |
||  |  |
| --- | --- |
| Entry number | 00-055-0592 |
| Total number of peaks | 41 |
 Space group | P63/m |
 Crystal system | hexagonal || Unit cell | a= 9.4189 Å c= 6.8827 Å |
| Calc. density | 3.155 g/cm³ |
| Reference | Tas, A., "", Powder Diffr. **16**, 102 (2001) |

## Selection Criteria

### Elements:

|  |  |
| --- | --- |
| ***Elements that must NOT be present:*** | All elements not mentioned above |

## Rietveld Refinement using FullProf

|  |
| --- |
| Calculation was not run or did not converge. |

## Crystallite Size Estimation using Scherrer Formula

|  |
| --- |
| Calculation was not run. |

## Integrated Profile Areas

### Based on calculated profile

|  |  |  |
| --- | --- | --- |
| ***Profile area*** | ***Counts*** | ***Amount*** |
| Overall diffraction profile | 125569 | 100.00% |
| Background radiation | 5186 | 4.13% |
| Diffraction peaks | 120384 | 95.87% |
| Peak area belonging to selected phases | 117926 | 93.91% |
| *Peak area of phase A (Calcium Hydrogen Phosphate)* | *48611* | *38.71%* |
| *Peak area of phase B (Calcium Phosphate Hydroxide Apatite-(CaOH), syn)* | *64114* | *51.06%* |
| Unidentified peak area | 7644 | 6.09% |

## Diffraction Pattern Graphics

  
  
PDF Database Copyright International Centre for Diffraction Data (ICDD)
Match! Copyright © 2003-2017 CRYSTAL IMPACT, Bonn, Germany
