# Supplementary material for: Microstructural and chemical characterization of radiation-induced carious dentin of teeth submitted to ionizing radiation as a head and neck cancer therapy
Source: PLoS One. 2025 Dec 12;20(12):e0337062. doi: 10.1371/journal.pone.0337062 (PMC12700452; doi:10.1371/journal.pone.0337062)
Supplement: S3 Data — (ZIP) [file pone.0337062.s003.zip › BrunaOdo/crr_mesmo/Theta = 10.0000 ()_Report.pdf]

Match! Phase Analysis Report

Paulo Soares

Sample: Theta = 10.0000 ()

Sample Data

File name	crr_mesmo.RAW
File path	C:/xddat/BrunaOdo/crr_mesmo
Data collected	Sep 17, 2021 15:29:10
Data range	15.000° - 55.000°
Number of points	2001
Step size	0.020
Rietveld refinement converged	No
Alpha2 subtracted	No
Background subtr.	Yes
Data smoothed	Yes
Radiation	X-rays
Wavelength	1.540600 Å

Matched Phases

Index	*Amount (%)*	*Name*	*Formula sum*
A		Calcium Hydrogen Phosphate	Ca H2 P2 O7
B		Calcium Phosphate Hydroxide Apatite-(CaOH), syn	(Ca)10 (P O4)6 (O H)2
	6.1	*Unidentified peak area*	

A: Calcium Hydrogen Phosphate

Formula sum	Ca H2 P2 O7
Entry number	00-051-0200
Total number of peaks	115
Space group	C2/c
Crystal system	monoclinic
Unit cell	a= 7.3294 Å b= 8.1300 Å c= 9.7665 Å β= 101.239 °
I/Ic	2.15
Calc. density	2.514 g/cm³
Reference	Schneider, M., Trommer, J., Wilde, L., Inst. f. Angewandte Chemie, Berlin, Germany., "", ICDD Grant-in-Aid , (1999)

B: Calcium Phosphate Hydroxide Apatite-(CaOH), syn

Formula sum	(Ca)10 (P O4)6 (O H)2
Entry number	00-055-0592
Total number of peaks	41
Space group	P63/m
Crystal system	hexagonal
Unit cell	a= 9.4189 Å c= 6.8827 Å
Calc. density	3.155 g/cm³
Reference	Tas, A., "", Powder Diffr. **16**, 102 (2001)

Selection Criteria

Elements:

Elements that must NOT be present:	All elements not mentioned above

Rietveld Refinement using FullProf

Calculation was not run or did not converge.

Crystallite Size Estimation using Scherrer Formula

Calculation was not run.

Integrated Profile Areas

Based on calculated profile

Profile area	*Counts*	*Amount*
Overall diffraction profile	125569	100.00%
Background radiation	5186	4.13%
Diffraction peaks	120384	95.87%
Peak area belonging to selected phases	117926	93.91%
Peak area of phase A (Calcium Hydrogen Phosphate)	*48611*	*38.71%*
Peak area of phase B (Calcium Phosphate Hydroxide Apatite-(CaOH), syn)	*64114*	*51.06%*
Unidentified peak area	7644	6.09%

Diffraction Pattern Graphics

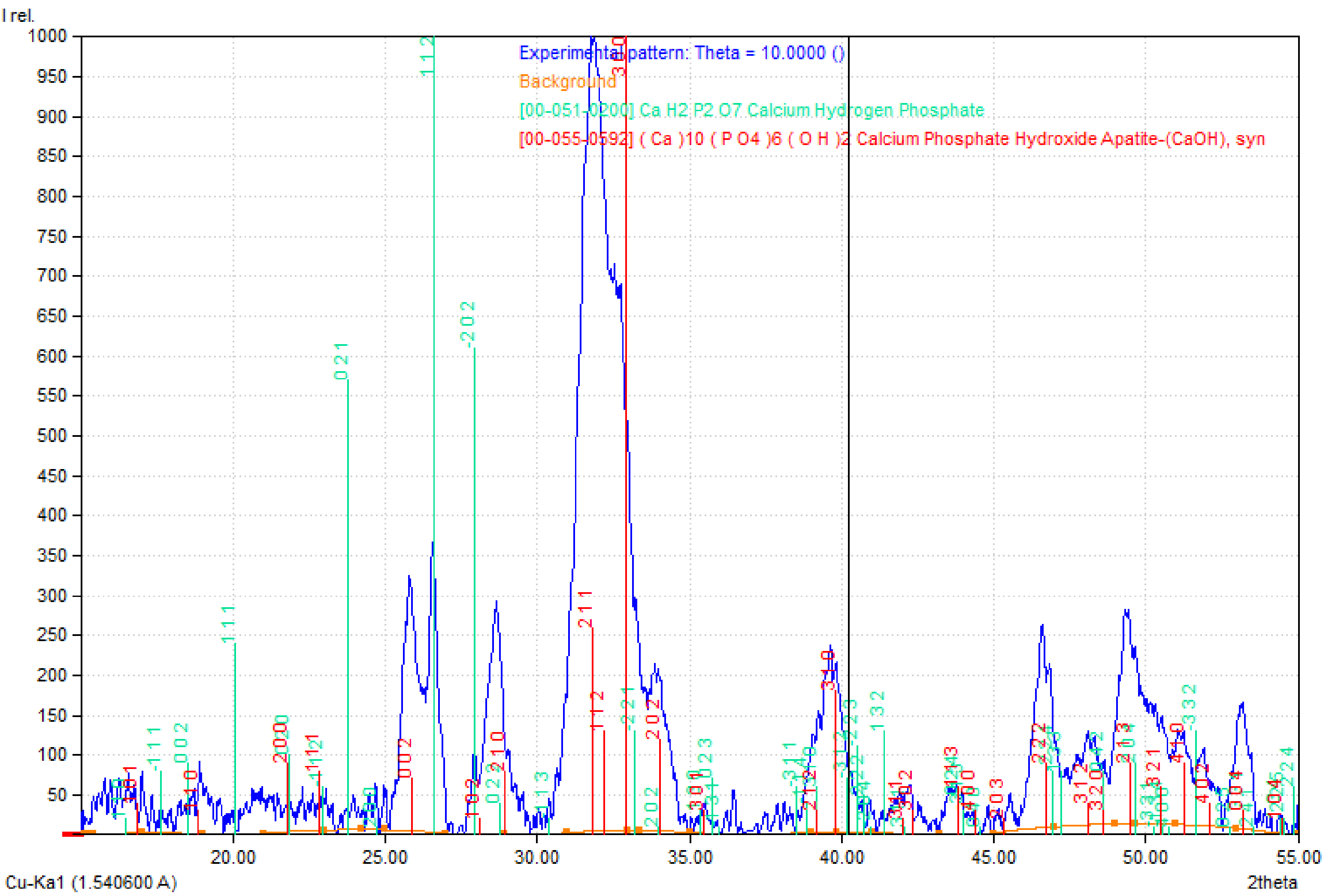

PDF Database Copyright International Centre for Diffraction Data (ICDD)
Match! Copyright © 2003-2017 CRYSTAL IMPACT, Bonn, Germany
