# Supplementary material for: Microstructural and chemical characterization of radiation-induced carious dentin of teeth submitted to ionizing radiation as a head and neck cancer therapy
Source: PLoS One. 2025 Dec 12;20(12):e0337062. doi: 10.1371/journal.pone.0337062 (PMC12700452; doi:10.1371/journal.pone.0337062)
Supplement: S3 Data — (ZIP) [file pone.0337062.s003.zip › BrunaOdo/dentina_irradiada/Theta = 10.0000 ()_Report.htm]

Match! message


## 

# Match! Phase Analysis Report

## Paulo Soares

## Sample: Theta = 10.0000 ()

|  |
| --- |
| ***Sample Data*** |
| File name | dentina\_irradiada.RAW |
| File path | C:/xddat/BrunaOdo/dentina\_irradiada |
| Data collected | Sep 17, 2021 17:20:21 |
| Data range | 15.000º - 55.000º |
 Number of points | 2001 || Step size | 0.020 |
| Rietveld refinement converged | No |
| Alpha2 subtracted | No |
| Background subtr. | Yes |
| Data smoothed | Yes |
| Radiation | X-rays |
| Wavelength | 1.540600 Å |

## Matched Phases

|  |  |  |  |
| --- | --- | --- | --- |
| ***Index*** | ***Amount (%)*** | ***Name*** | ***Formula sum*** |
| A |  | Calcium Phosphate Hydroxide Apatite-(CaOH), syn | Ca5 ( P O4 )3 ( O H ) |
|  | *1.7* | *Unidentified peak area* |  |

|  |
| --- |
| ***A: Calcium Phosphate Hydroxide Apatite-(CaOH), syn*** |
| Formula sum | Ca5 ( P O4 )3 ( O H ) |
||  |  |
| --- | --- |
| Entry number | 01-079-5683 |
| Total number of peaks | 136 |
 Space group | P63/m |
 Crystal system | hexagonal || Unit cell | a= 9.4190 Å c= 6.8812 Å |
| I/Ic | 1.15 |
| Calc. density | 3.155 g/cm³ |
| Reference | Get`man, E.I., Loboda, S.N., Tkachenko, T.V., Yablochkova, N.V., Chebyshev, K.A., "", Zh. Neorg. Khim. **55**, 344 (2010) |

## Rietveld Refinement using FullProf

|  |
| --- |
| Calculation was not run or did not converge. |

## Crystallite Size Estimation using Scherrer Formula

|  |
| --- |
| Calculation was not run. |

## Integrated Profile Areas

### Based on calculated profile

|  |  |  |
| --- | --- | --- |
| ***Profile area*** | ***Counts*** | ***Amount*** |
| Overall diffraction profile | 116120 | 100.00% |
| Background radiation | 9577 | 8.25% |
| Diffraction peaks | 106543 | 91.75% |
| Peak area belonging to selected phases | 114177 | 98.33% |
| *Peak area of phase A (Calcium Phosphate Hydroxide Apatite-(CaOH), syn)* | *112608* | *96.98%* |
| Unidentified peak area | 1943 | 1.67% |

## Diffraction Pattern Graphics

  
  
PDF Database Copyright International Centre for Diffraction Data (ICDD)
Match! Copyright © 2003-2017 CRYSTAL IMPACT, Bonn, Germany
