# Supplementary material for: Microstructural and chemical characterization of radiation-induced carious dentin of teeth submitted to ionizing radiation as a head and neck cancer therapy
Source: PLoS One. 2025 Dec 12;20(12):e0337062. doi: 10.1371/journal.pone.0337062 (PMC12700452; doi:10.1371/journal.pone.0337062)
Supplement: S3 Data — (ZIP) [file pone.0337062.s003.zip › BrunaOdo/dentina_irradiada/Theta = 10.0000 ()_Report.pdf]

Match! Phase Analysis Report

Paulo Soares

Sample: Theta = 10.0000 ()

Sample Data	
File name	dentina_irradiada.RAW
File path	C:/xddat/BrunaOdo/dentina_irradiada
Data collected	Sep 17, 2021 17:20:21
Data range	15.000° - 55.000°
Number of points	2001
Step size	0.020
Rietveld refinement converged	No
Alpha2 subtracted	No
Background subtr.	Yes
Data smoothed	Yes
Radiation	X-rays
Wavelength	1.540600 Å

Matched Phases

Index	*Amount (%)*	*Name*	*Formula sum*
A		Calcium Phosphate Hydroxide Apatite-(CaOH), syn	Ca5 (P O4)3 (O H)
	1.7	*Unidentified peak area*	

A: Calcium Phosphate Hydroxide Apatite-(CaOH), syn	
Formula sum	Ca5 (P O4)3 (O H)
Entry number	01-079-5683
Total number of peaks	136
Space group	P63/m
Crystal system	hexagonal
Unit cell	a= 9.4190 Å c= 6.8812 Å
I/Ic	1.15
Calc. density	3.155 g/cm³
Reference	Get`man, E.I., Loboda, S.N., Tkachenko, T.V., Yablochkova, N.V., Chebyshev, K.A., "", Zh. Neorg. Khim. **55**, 344 (2010)

Rietveld Refinement using FullProf

Calculation was not run or did not converge.

Crystallite Size Estimation using Scherrer Formula

Calculation was not run.

Integrated Profile Areas

Based on calculated profile

Profile area	*Counts*	*Amount*
Overall diffraction profile	116120	100.00%
Background radiation	9577	8.25%
Diffraction peaks	106543	91.75%
Peak area belonging to selected phases	114177	98.33%
Peak area of phase A (Calcium Phosphate Hydroxide Apatite-(CaOH), syn)	*112608*	*96.98%*
Unidentified peak area	1943	1.67%

Diffraction Pattern Graphics

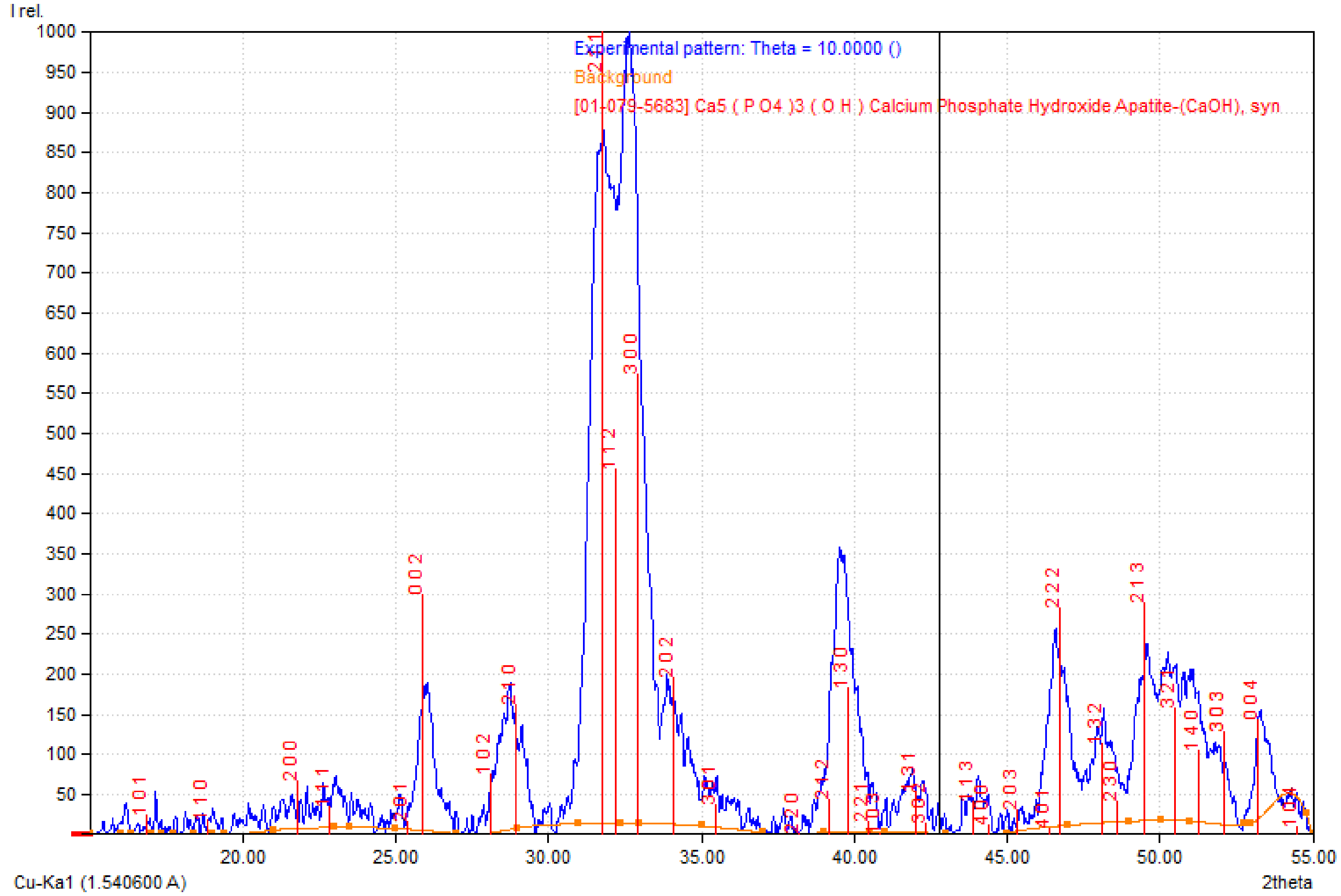

PDF Database Copyright International Centre for Diffraction Data (ICDD)
Match! Copyright © 2003-2017 CRYSTAL IMPACT, Bonn, Germany
