# Supplementary material for: Microstructural and chemical characterization of radiation-induced carious dentin of teeth submitted to ionizing radiation as a head and neck cancer therapy
Source: PLoS One. 2025 Dec 12;20(12):e0337062. doi: 10.1371/journal.pone.0337062 (PMC12700452; doi:10.1371/journal.pone.0337062)
Supplement: S3 Data — (ZIP) [file pone.0337062.s003.zip › BrunaOdo/higido-ds/Theta = 10.0000 ()_Report.htm]

Match! message


## 

# Match! Phase Analysis Report

## Paulo Soares

## Sample: Theta = 10.0000 ()

|  |
| --- |
| ***Sample Data*** |
| File name | higido-ds.RAW |
| File path | C:/xddat/BrunaOdo/higido-ds |
| Data collected | Sep 17, 2021 16:30:14 |
| Data range | 15.000º - 55.000º |
 Number of points | 2001 || Step size | 0.020 |
| Rietveld refinement converged | No |
| Alpha2 subtracted | No |
| Background subtr. | Yes |
| Data smoothed | Yes |
| Radiation | X-rays |
| Wavelength | 1.540600 Å |

## Matched Phases

|  |  |  |  |
| --- | --- | --- | --- |
| ***Index*** | ***Amount (%)*** | ***Name*** | ***Formula sum*** |
| A |  | Calcium Phosphate Hydroxide Hydroxylapatite, syn | Ca5 ( P O4 )3 O H |
|  | *0.5* | *Unidentified peak area* |  |

|  |
| --- |
| ***A: Calcium Phosphate Hydroxide Hydroxylapatite, syn*** |
| Formula sum | Ca5 ( P O4 )3 O H |
||  |  |
| --- | --- |
| Entry number | 01-076-0694 |
| Total number of peaks | 199 |
 Space group | P21/b |
 Crystal system | monoclinic || Unit cell | a= 9.4214 Å b= 18.8428 Å c= 6.8814 Å γ= 120.000 º |
| I/Ic | 0.52 |
| Calc. density | 3.154 g/cm³ |
| Reference | Elliot, J.C., Mackie, P.E., Young, R.A., "", Science **180**, 1055 (1973) |

## Selection Criteria

### Elements:

|  |  |
| --- | --- |
| ***Elements that must NOT be present:*** | All elements not mentioned above |

## Rietveld Refinement using FullProf

|  |
| --- |
| Calculation was not run or did not converge. |

## Crystallite Size Estimation using Scherrer Formula

|  |
| --- |
| Calculation was not run. |

## Integrated Profile Areas

### Based on calculated profile

|  |  |  |
| --- | --- | --- |
| ***Profile area*** | ***Counts*** | ***Amount*** |
| Overall diffraction profile | 195338 | 100.00% |
| Background radiation | 13349 | 6.83% |
| Diffraction peaks | 181988 | 93.17% |
| Peak area belonging to selected phases | 194451 | 99.55% |
| *Peak area of phase A (Calcium Phosphate Hydroxide Hydroxylapatite, syn)* | *193917* | *99.27%* |
| Unidentified peak area | 886 | 0.45% |

## Diffraction Pattern Graphics

  
  
PDF Database Copyright International Centre for Diffraction Data (ICDD)
Match! Copyright © 2003-2017 CRYSTAL IMPACT, Bonn, Germany
