# Supplementary material for: Microstructural and chemical characterization of radiation-induced carious dentin of teeth submitted to ionizing radiation as a head and neck cancer therapy
Source: PLoS One. 2025 Dec 12;20(12):e0337062. doi: 10.1371/journal.pone.0337062 (PMC12700452; doi:10.1371/journal.pone.0337062)
Supplement: S3 Data — (ZIP) [file pone.0337062.s003.zip › BrunaOdo/higido-ds/Theta = 10.0000 ()_Report.pdf]

Match! Phase Analysis Report

Paulo Soares

Sample: Theta = 10.0000 ()

Sample Data	
File name	higido-ds.RAW
File path	C:/xddat/BrunaOdo/higido-ds
Data collected	Sep 17, 2021 16:30:14
Data range	15.000° - 55.000°
Number of points	2001
Step size	0.020
Rietveld refinement converged	No
Alpha2 subtracted	No
Background subtr.	Yes
Data smoothed	Yes
Radiation	X-rays
Wavelength	1.540600 Å

Matched Phases

Index	***Amount (%)***	***Name***	***Formula sum***
A		Calcium Phosphate Hydroxide Hydroxylapatite, syn	Ca5 (P O4)3 O H
	0.5	*Unidentified peak area*	

A: Calcium Phosphate Hydroxide Hydroxylapatite, syn	
Formula sum	Ca5 (P O4)3 O H
Entry number	01-076-0694
Total number of peaks	199
Space group	P21/b
Crystal system	monoclinic
Unit cell	a= 9.4214 Å b= 18.8428 Å c= 6.8814 Å γ= 120.000 °
I/Ic	0.52
Calc. density	3.154 g/cm³
Reference	Elliot, J.C., Mackie, P.E., Young, R.A., "", Science **180**, 1055 (1973)

Selection Criteria

Elements:

Elements that must NOT be present:	All elements not mentioned above

Rietveld Refinement using FullProf

Calculation was not run or did not converge.

Crystallite Size Estimation using Scherrer Formula

Calculation was not run.

Integrated Profile Areas

Based on calculated profile

Profile area	***Counts***	***Amount***
Overall diffraction profile	195338	100.00%
Background radiation	13349	6.83%
Diffraction peaks	181988	93.17%
Peak area belonging to selected phases	194451	99.55%
Peak area of phase A (Calcium Phosphate Hydroxide Hydroxylapatite, syn)	*193917*	*99.27%*
Unidentified peak area	886	0.45%

Diffraction Pattern Graphics

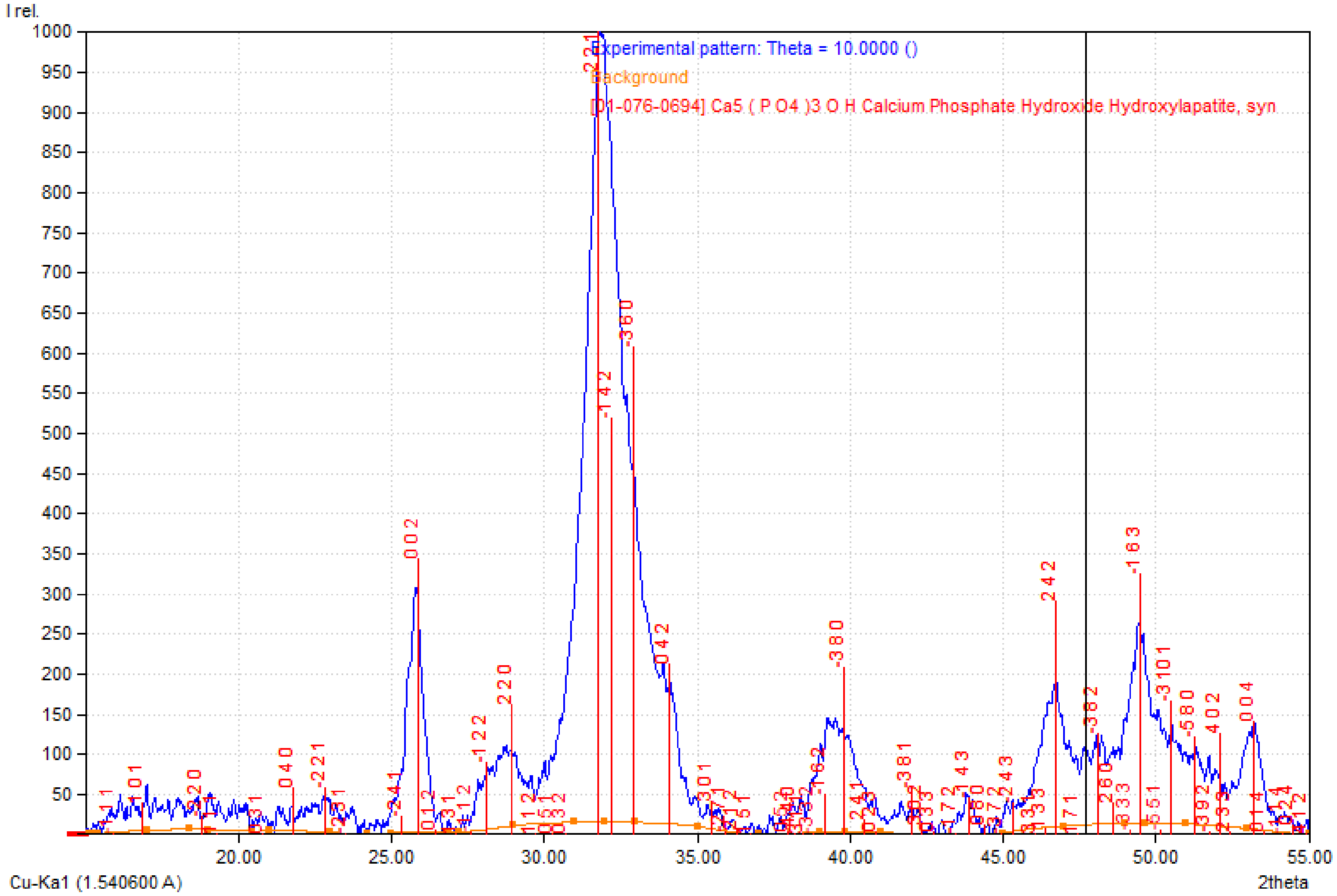

PDF Database Copyright International Centre for Diffraction Data (ICDD)
Match! Copyright © 2003-2017 CRYSTAL IMPACT, Bonn, Germany
