# Supplementary figures and images for: Microstructural and chemical characterization of radiation-induced carious dentin of teeth submitted to ionizing radiation as a head and neck cancer therapy (part 2 of 2)
Source: PLoS One. 2025 Dec 12;20(12):e0337062. doi: 10.1371/journal.pone.0337062 (PMC12700452; doi:10.1371/journal.pone.0337062)

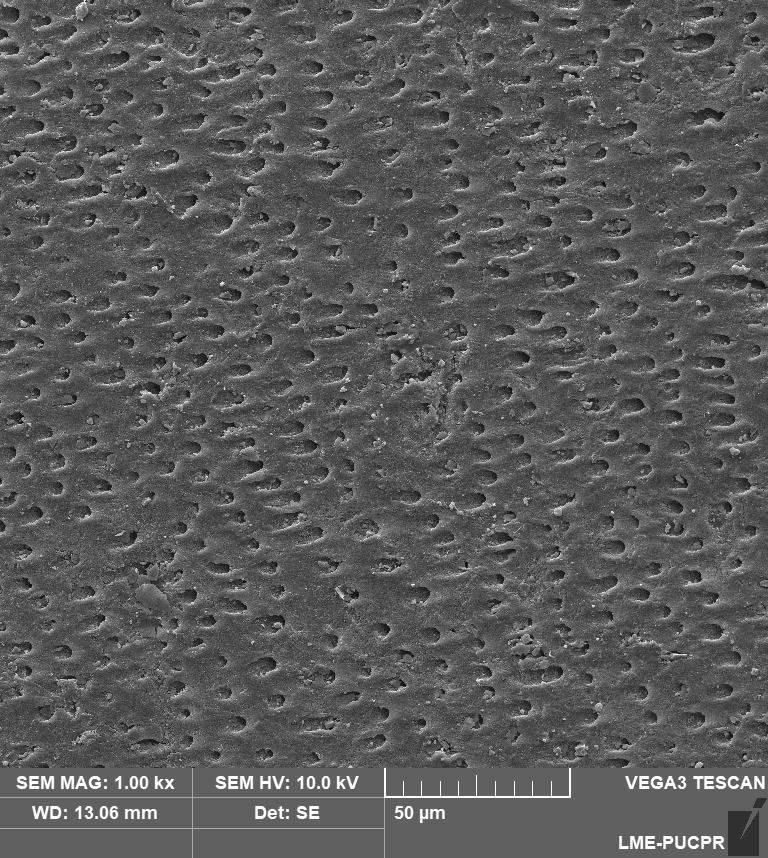

Supplement: S6 Data — (ZIP) [file pone.0337062.s006.zip › SEM/24Set21/crr_x1k.tif]

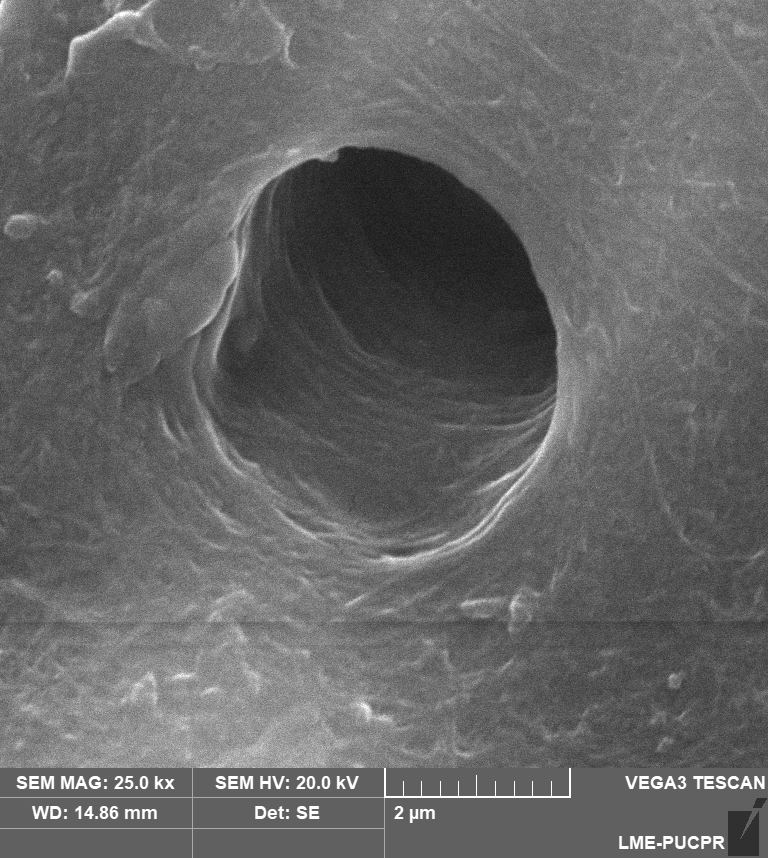

Supplement: S6 Data — (ZIP) [file pone.0337062.s006.zip › SEM/21Jan21/am2/am2_higido_25kx.tif]

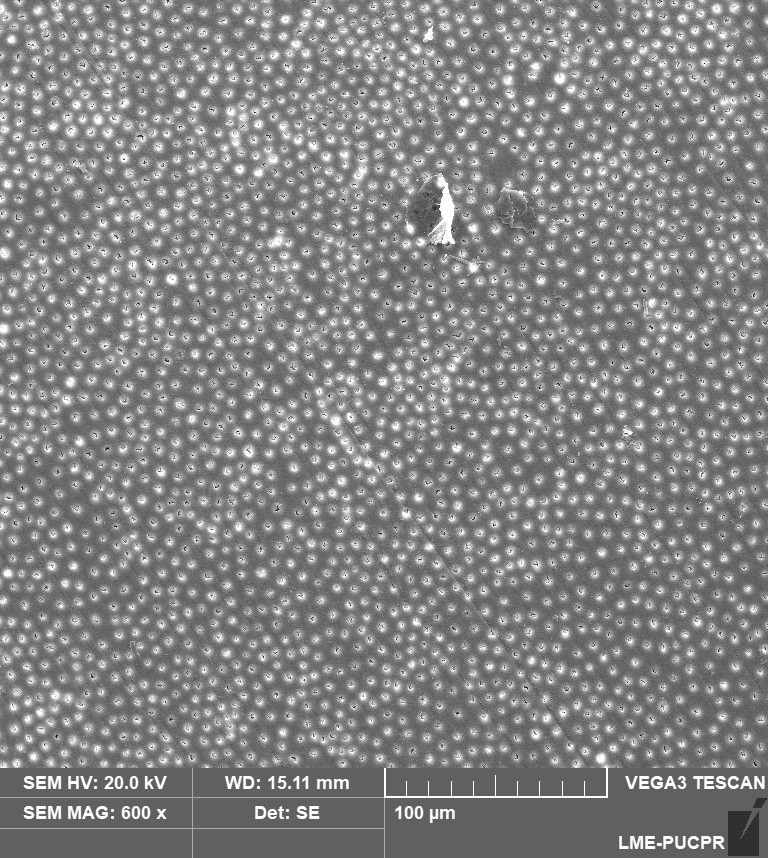

Supplement: S6 Data — (ZIP) [file pone.0337062.s006.zip › SEM/08Nov19/Dente_higido_600x_A.tif]

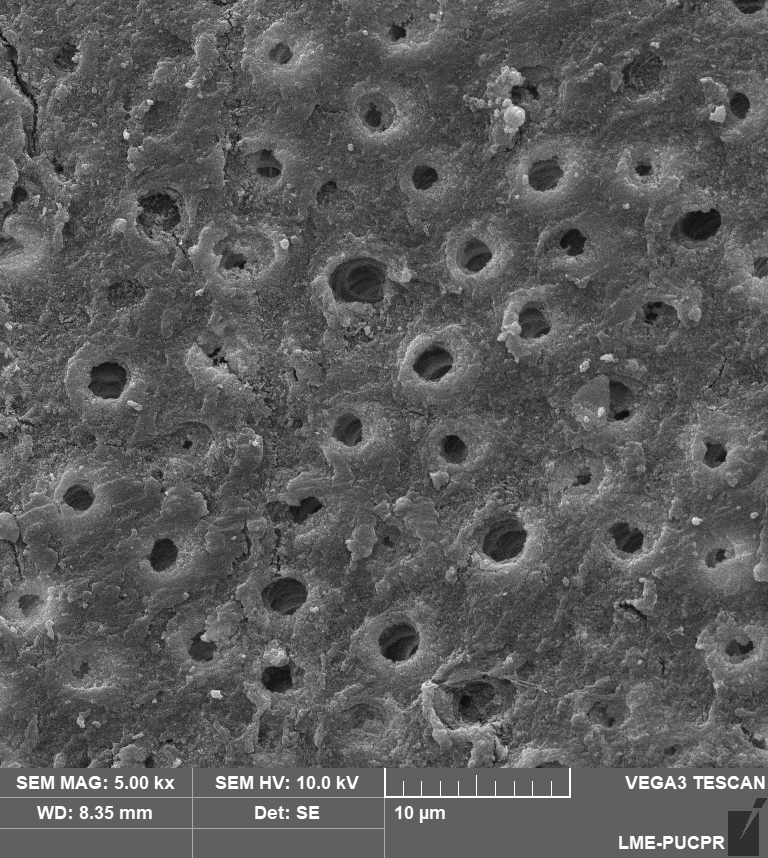

Supplement: S6 Data — (ZIP) [file pone.0337062.s006.zip › SEM/24Set21/irradiado2_x5k.tif]

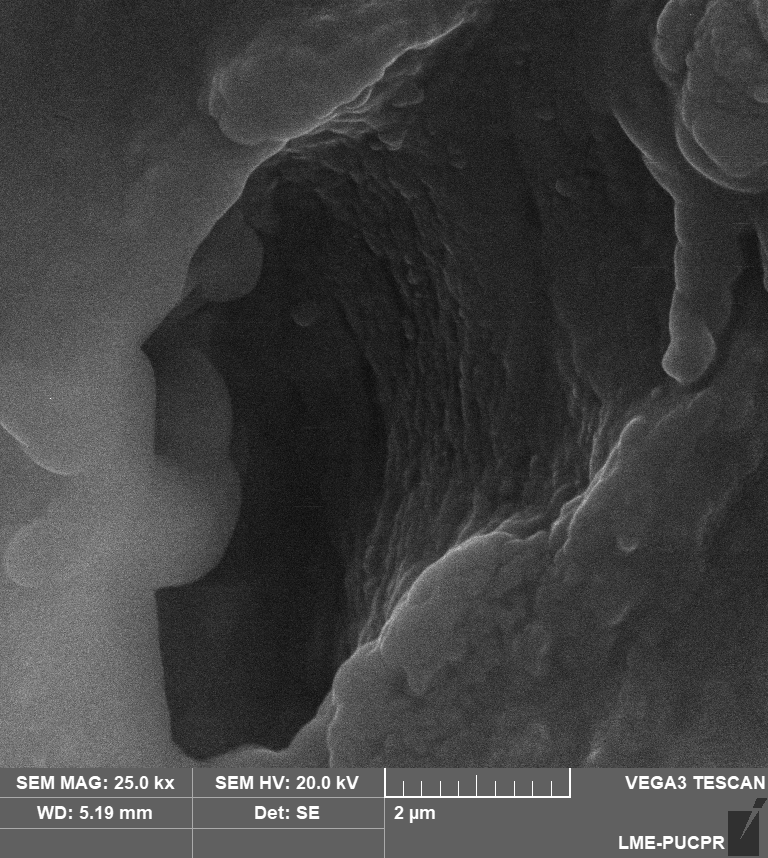

Supplement: S6 Data — (ZIP) [file pone.0337062.s006.zip › SEM/21Jan21/am1/am1_crr_25kx.tif]

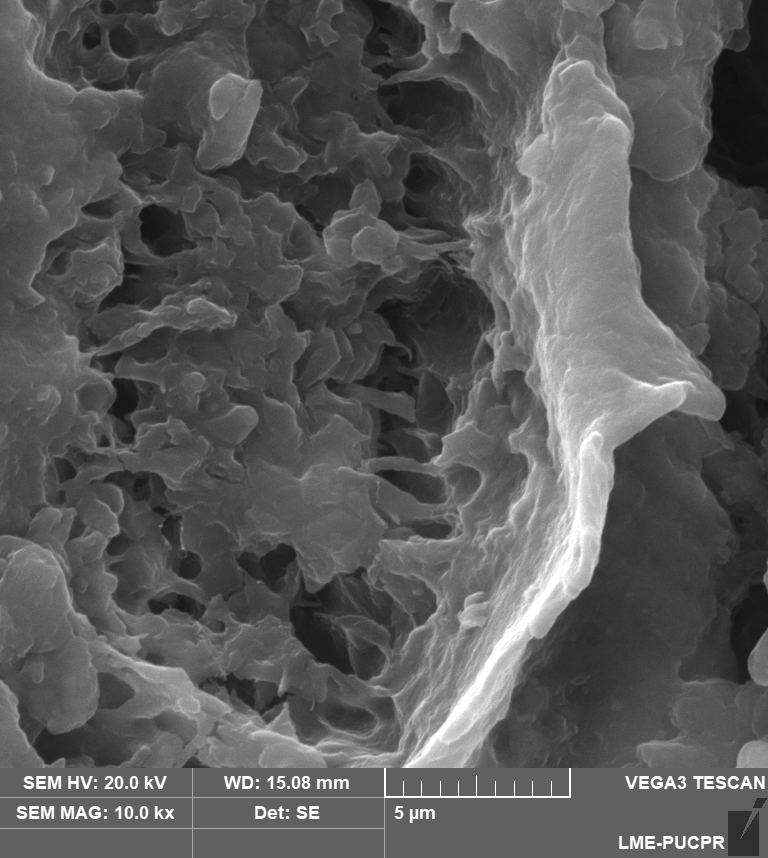

Supplement: S6 Data — (ZIP) [file pone.0337062.s006.zip › SEM/08Nov19/CRR_10Kx.tif]

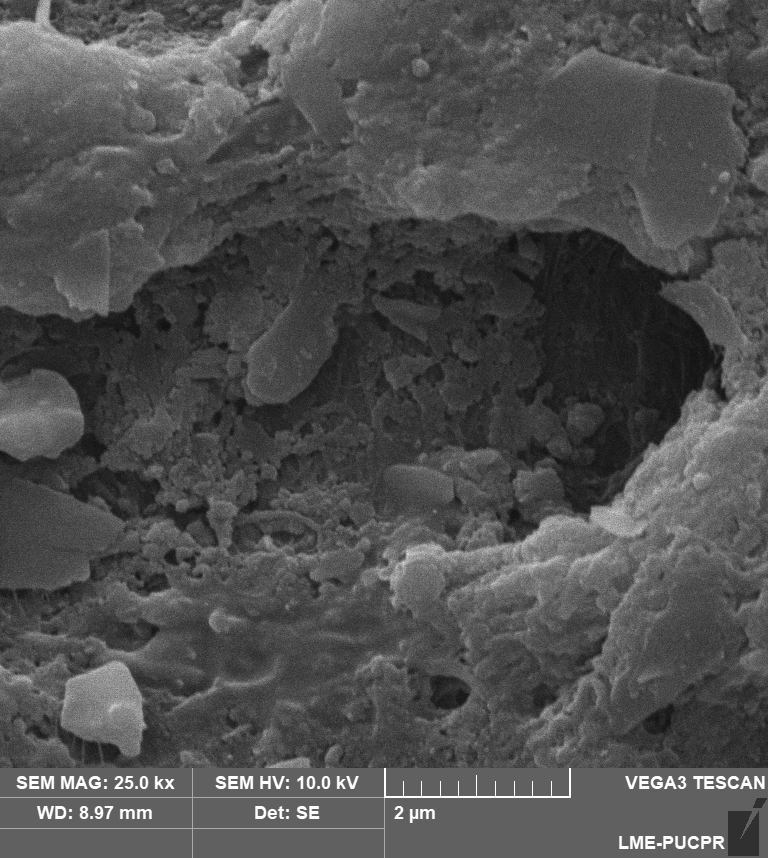

Supplement: S6 Data — (ZIP) [file pone.0337062.s006.zip › SEM/24Set21/crr_x25ka.tif]

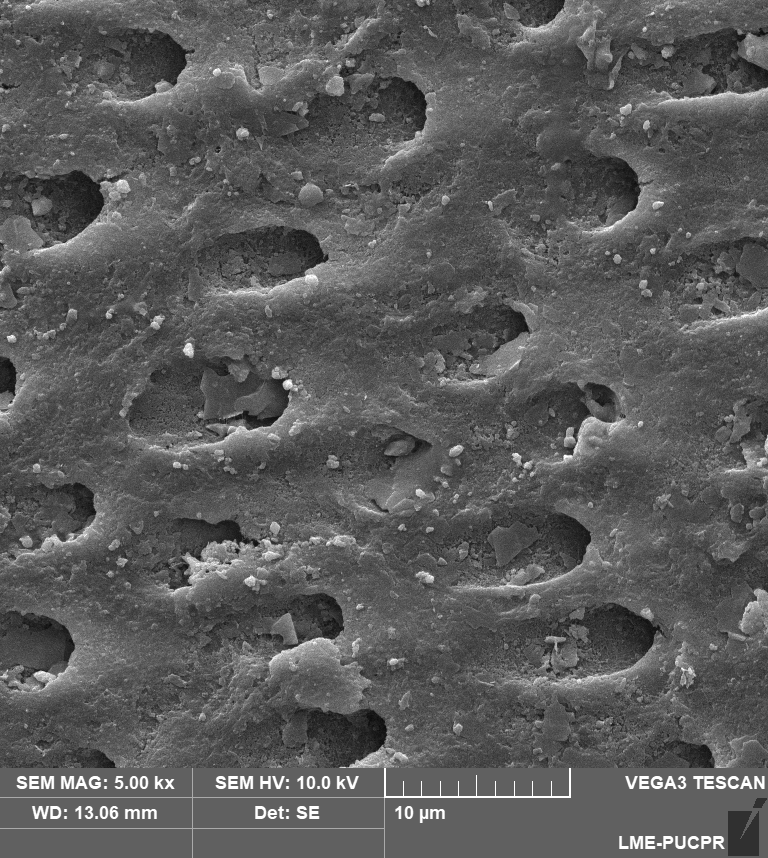

Supplement: S6 Data — (ZIP) [file pone.0337062.s006.zip › SEM/24Set21/crr_x5k.tif]

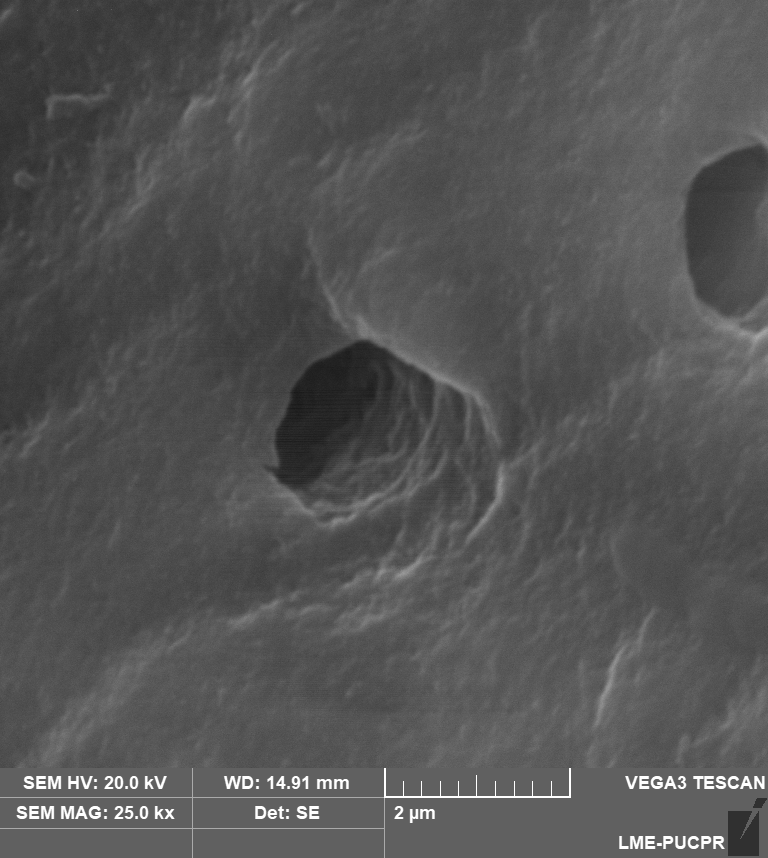

Supplement: S6 Data — (ZIP) [file pone.0337062.s006.zip › SEM/08Nov19/CRR2_25Kx.tif]

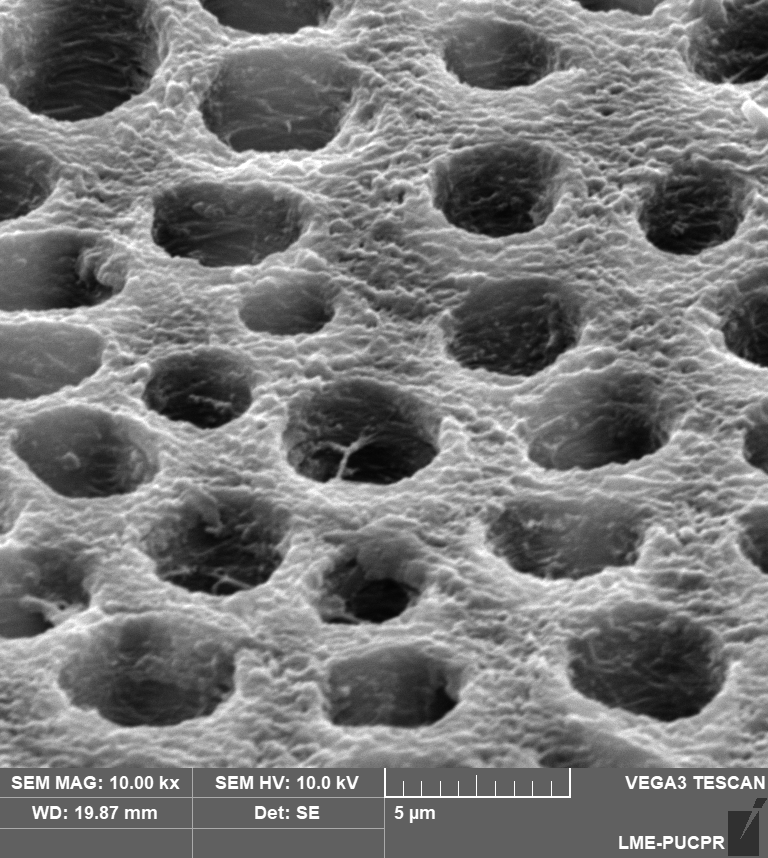

Supplement: S6 Data — (ZIP) [file pone.0337062.s006.zip › SEM/24Set21/higido_x10k tilt 55.tif]

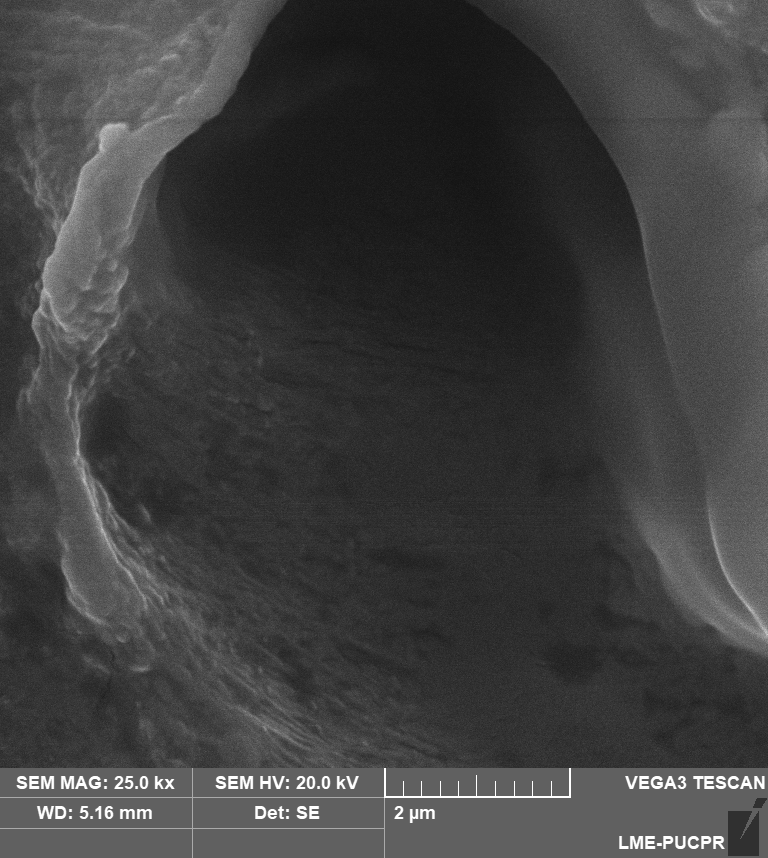

Supplement: S6 Data — (ZIP) [file pone.0337062.s006.zip › SEM/21Jan21/am2/am2_crr_25kx.tif]

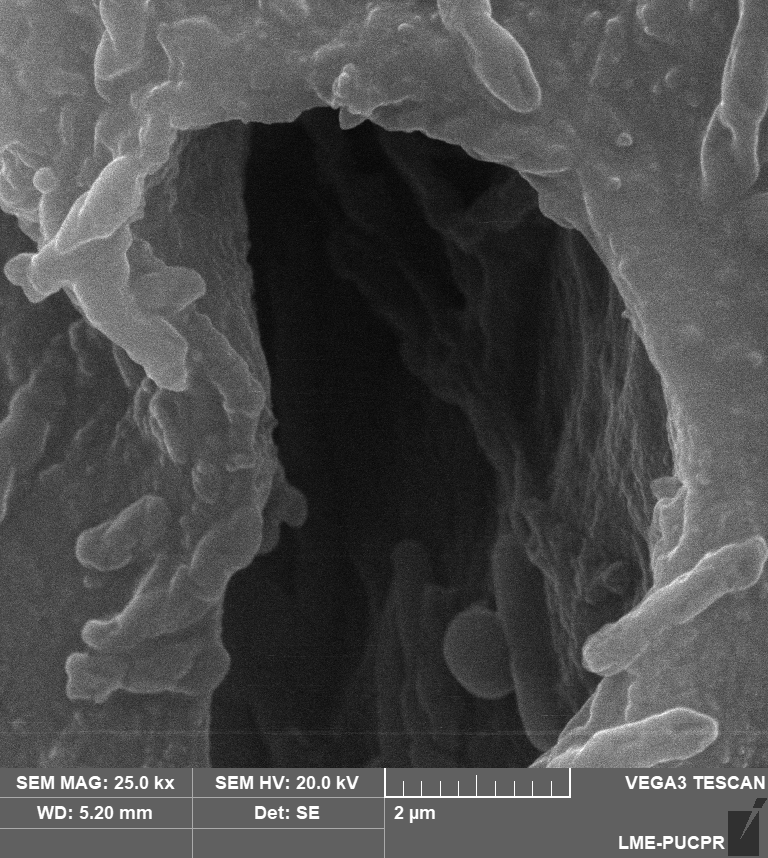

Supplement: S6 Data — (ZIP) [file pone.0337062.s006.zip › SEM/21Jan21/am1/am1_crr_25xka.tif]

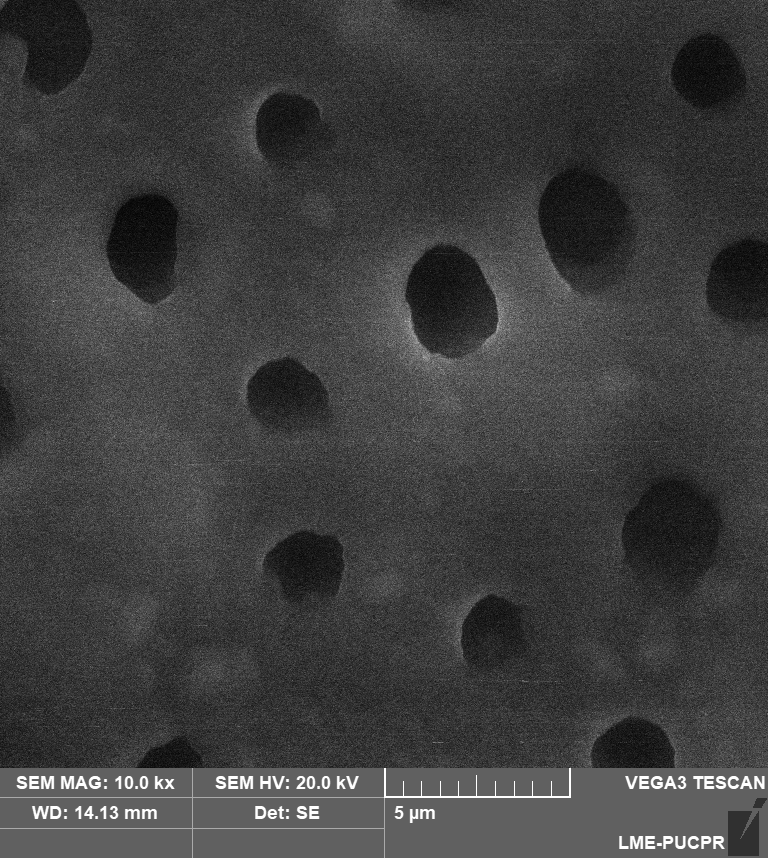

Supplement: S6 Data — (ZIP) [file pone.0337062.s006.zip › SEM/16Abr21/Am higido 3_x10k.tif]

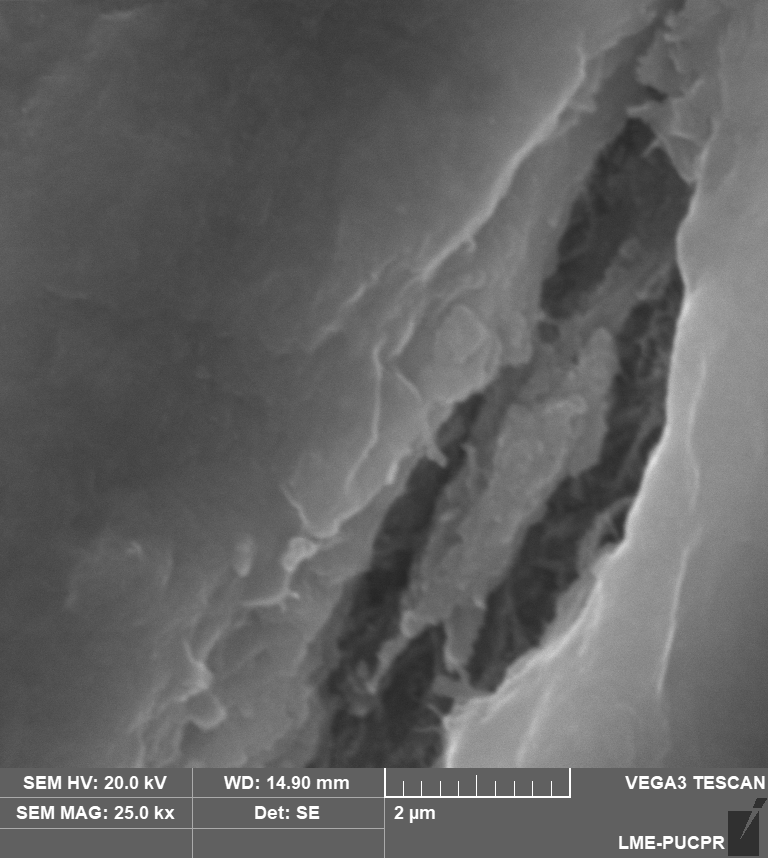

Supplement: S6 Data — (ZIP) [file pone.0337062.s006.zip › SEM/08Nov19/CRR_25Kx_B.tif]

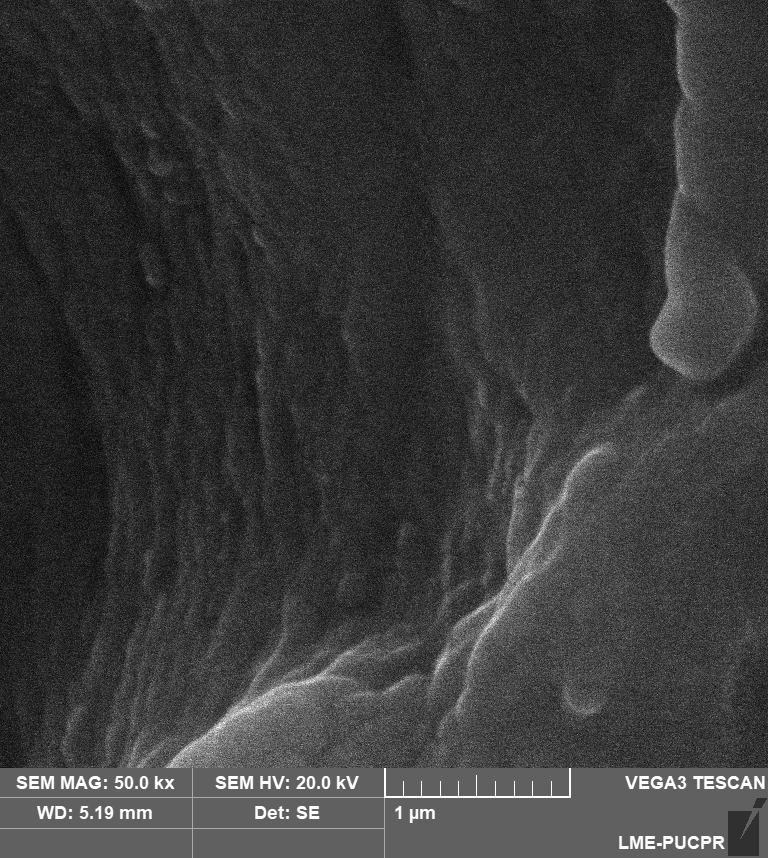

Supplement: S6 Data — (ZIP) [file pone.0337062.s006.zip › SEM/21Jan21/am1/am1_crr_50kx.tif]

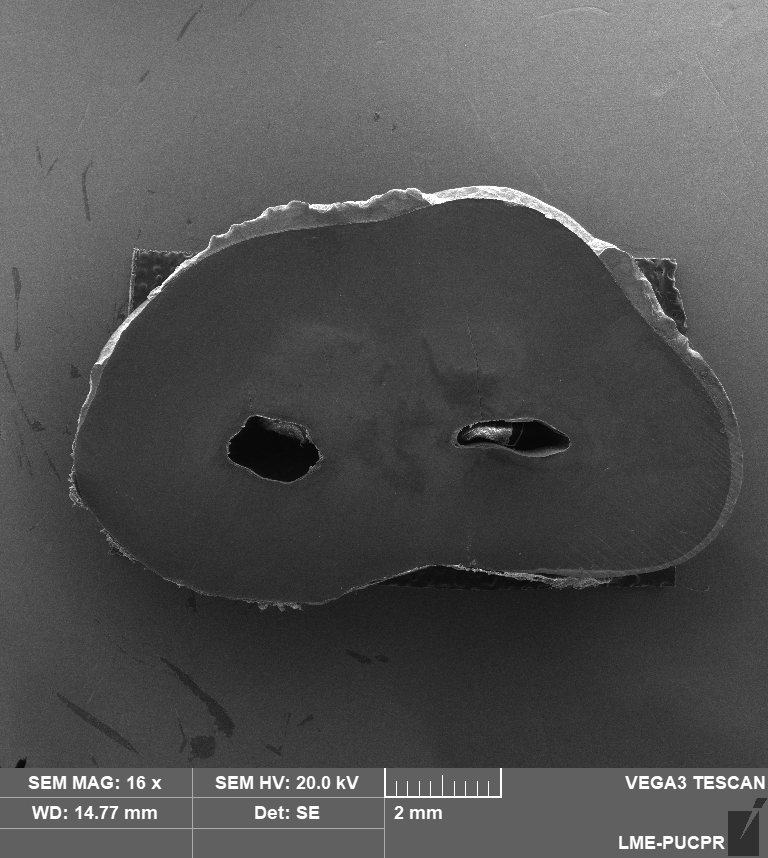

Supplement: S6 Data — (ZIP) [file pone.0337062.s006.zip › SEM/24Set21/higido_x16.tif]

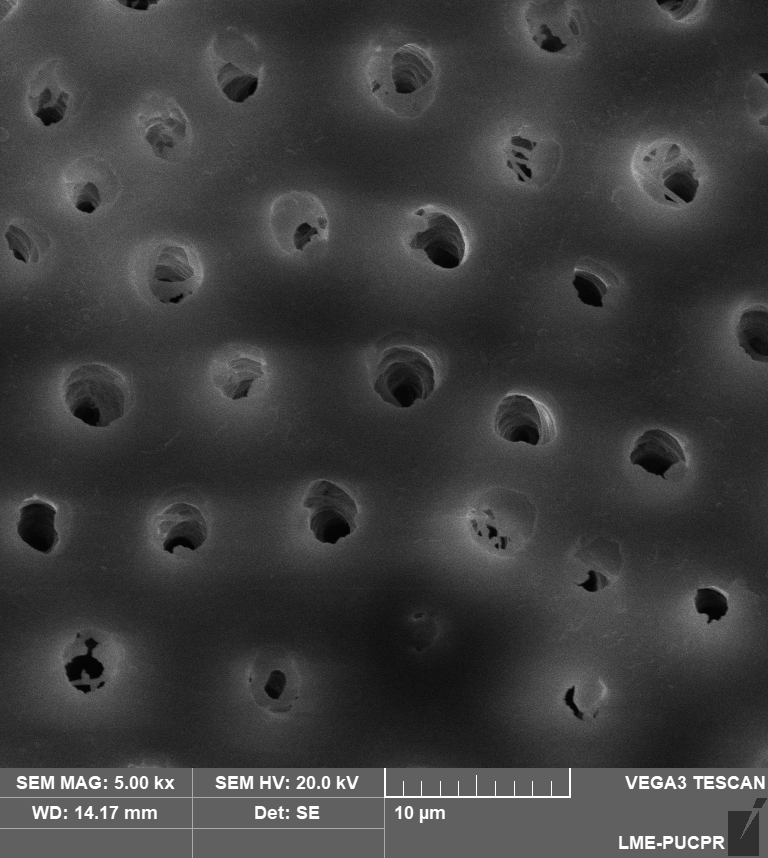

Supplement: S6 Data — (ZIP) [file pone.0337062.s006.zip › SEM/16Abr21/Am higido 2_x5ka.tif]

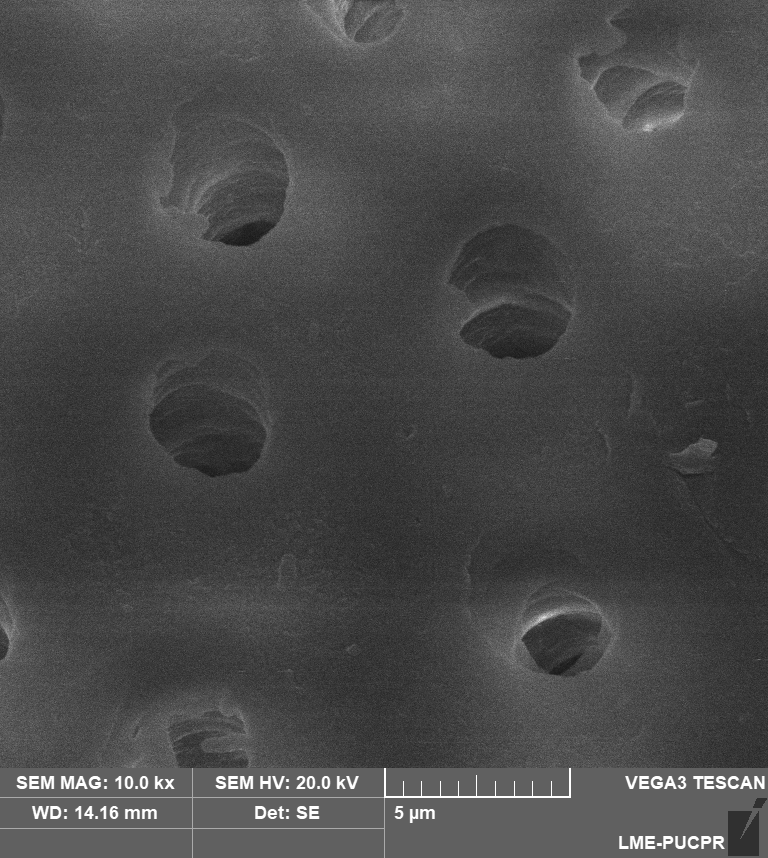

Supplement: S6 Data — (ZIP) [file pone.0337062.s006.zip › SEM/16Abr21/Am higido 2_x10k.tif]

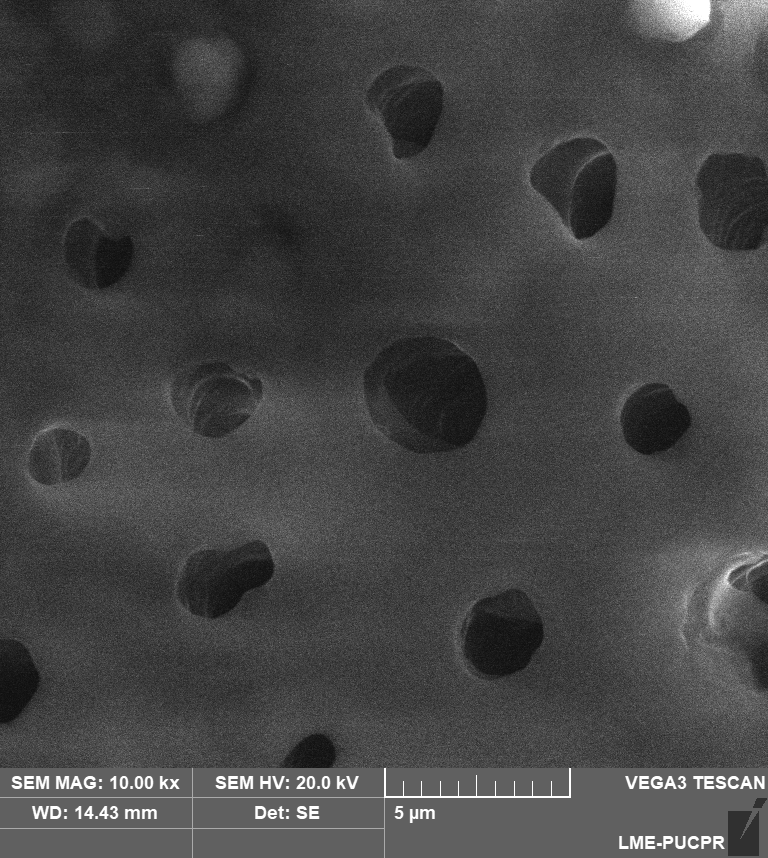

Supplement: S6 Data — (ZIP) [file pone.0337062.s006.zip › SEM/16Abr21/Am higido 3_x10ka.tif]

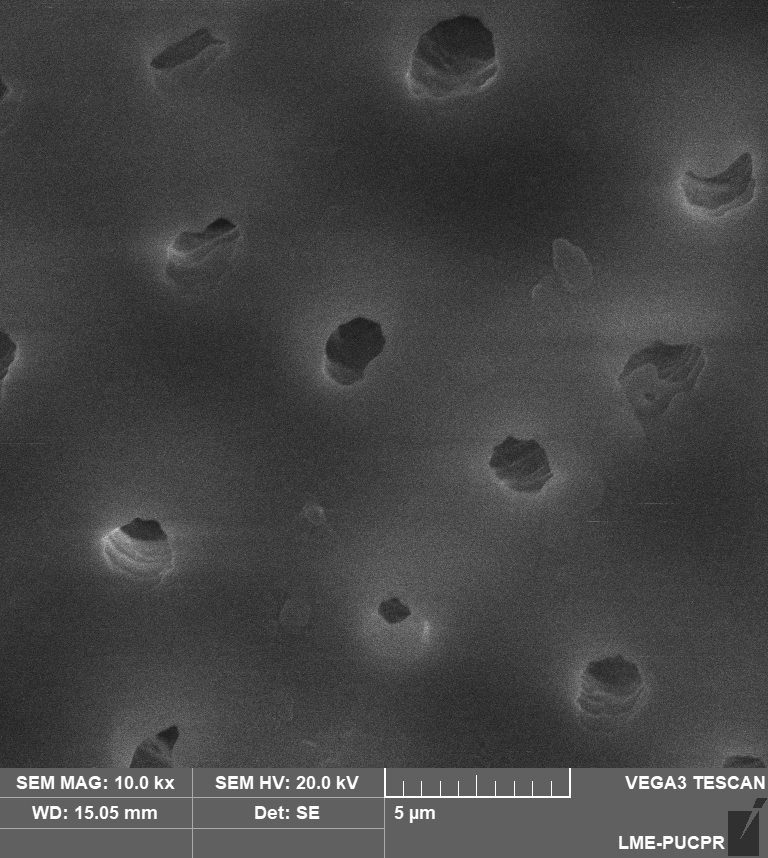

Supplement: S6 Data — (ZIP) [file pone.0337062.s006.zip › SEM/16Abr21/Am higido 1_x10k.tif]

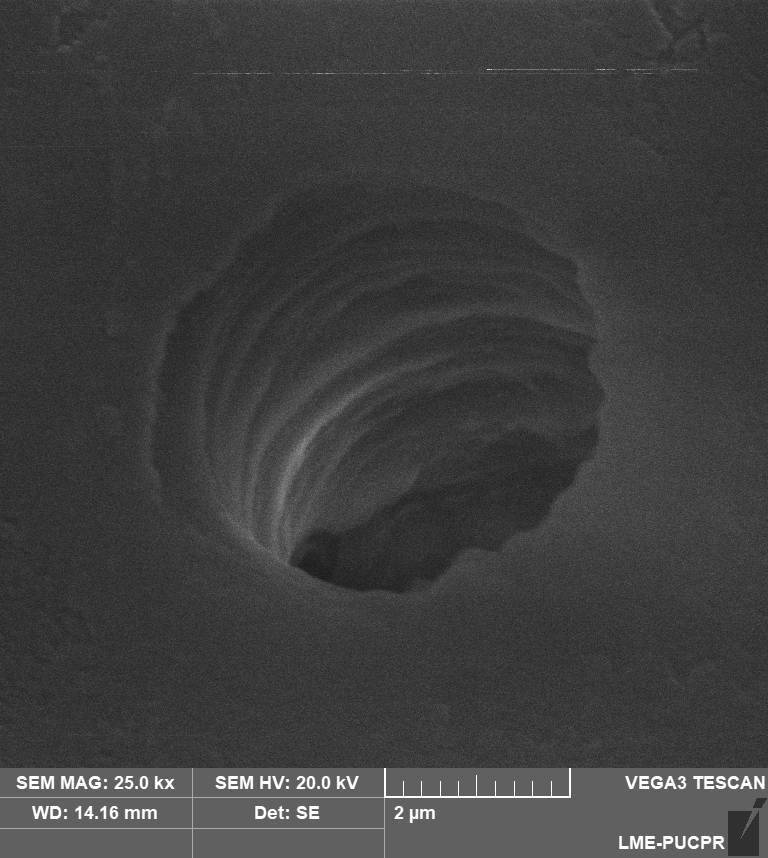

Supplement: S6 Data — (ZIP) [file pone.0337062.s006.zip › SEM/16Abr21/Am higido 2_x25k.tif]

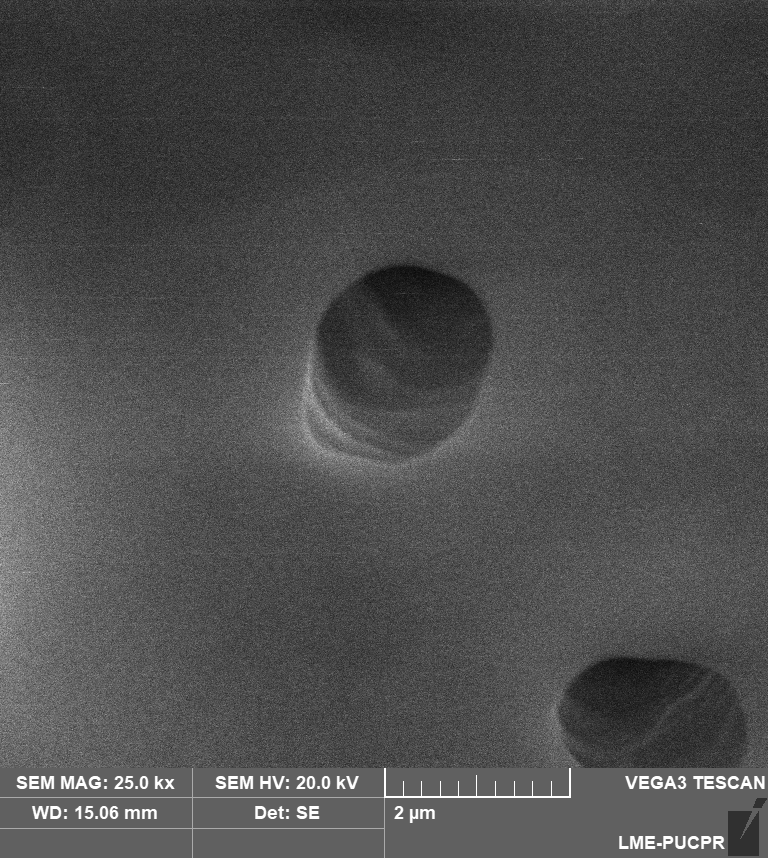

Supplement: S6 Data — (ZIP) [file pone.0337062.s006.zip › SEM/16Abr21/Am higido 1_x25ka.tif]

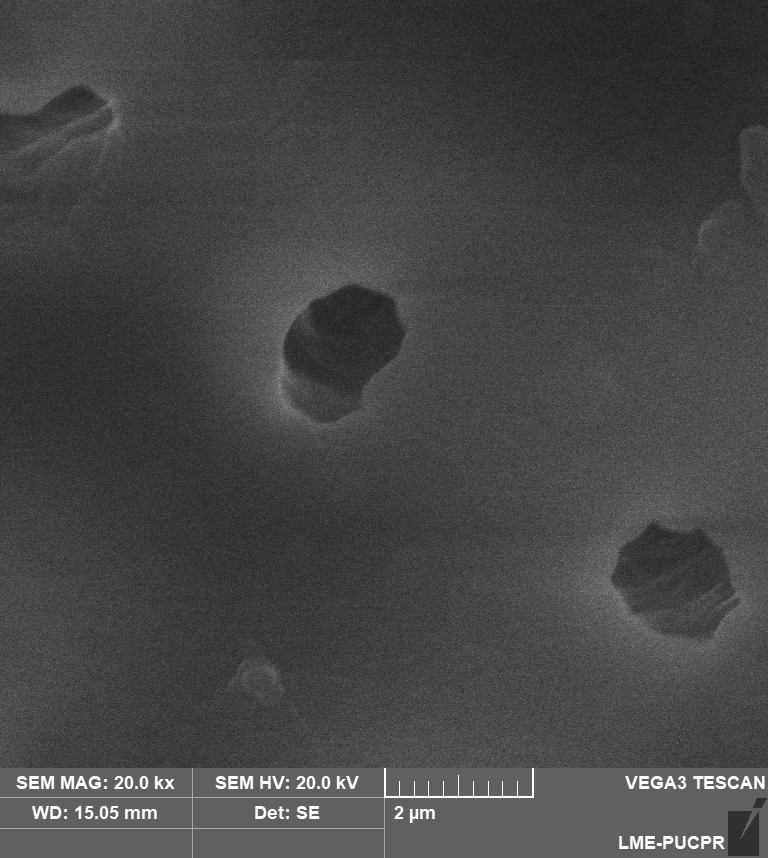

Supplement: S6 Data — (ZIP) [file pone.0337062.s006.zip › SEM/16Abr21/Am higido 1_x20k.tif]

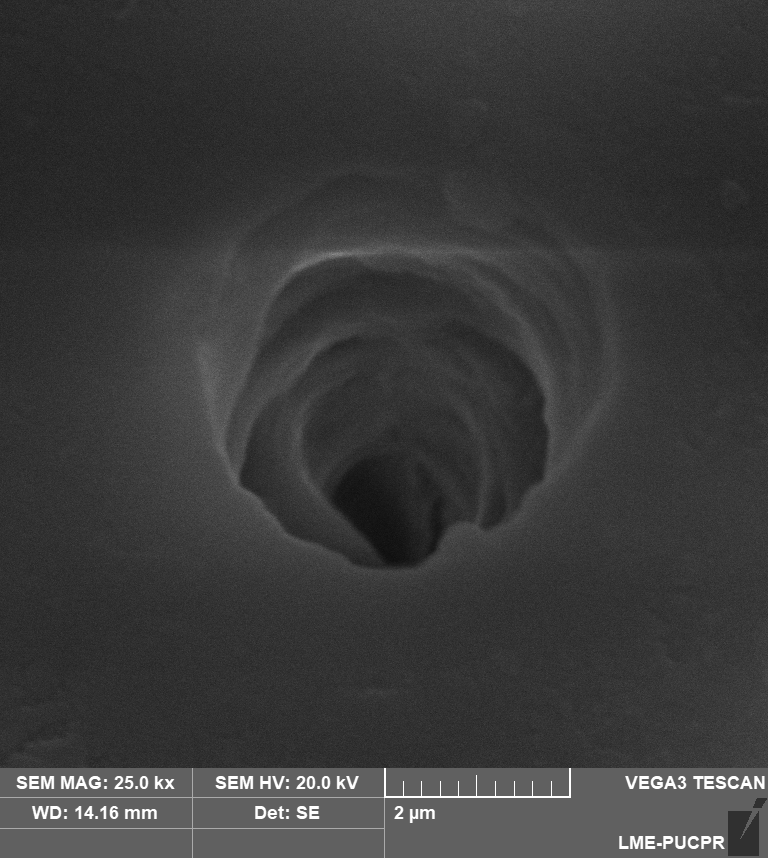

Supplement: S6 Data — (ZIP) [file pone.0337062.s006.zip › SEM/16Abr21/Am higido 2_x25ka.tif]

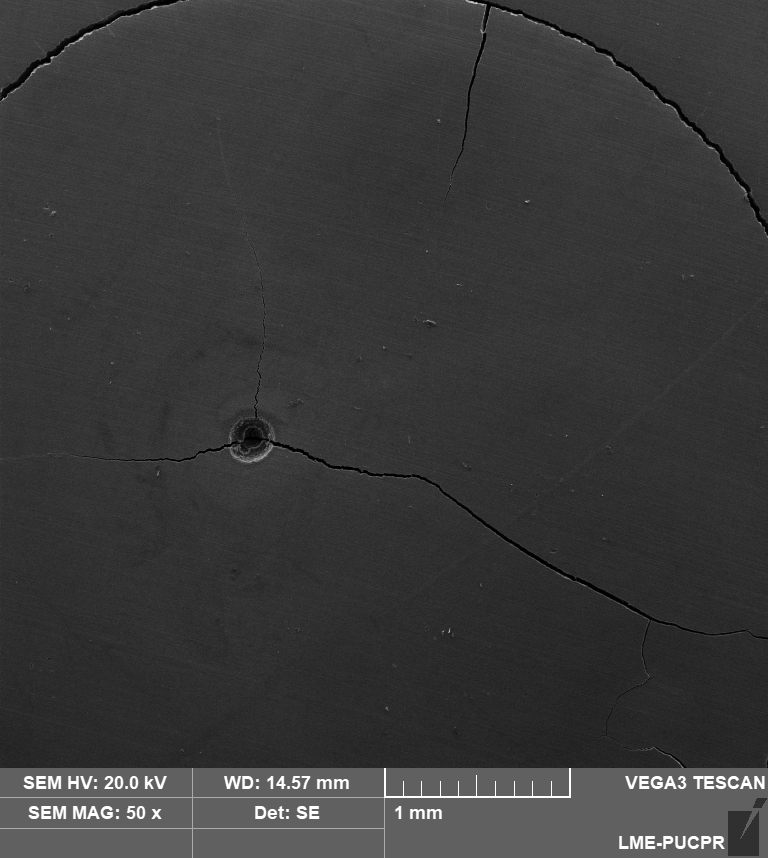

Supplement: S6 Data — (ZIP) [file pone.0337062.s006.zip › SEM/08Nov19/Dente_higido2_50x.tif]
